# Innovation and Biodiversity: Patent Trends in Therapeutic and Cosmetic Use of Scientifically Relevant Pantanal Plants

**DOI:** 10.3390/plants15172632

**Published:** 2026-08-28

**Authors:** Danielle Teixeira Freire, Douglas Dourado, Thalisson Amorim de Souza, Éverton do Nascimento Alencar

**Affiliations:** 1 Laboratory of Micro and Nanostructured Systems, College of Pharmaceutical Sciences, Food and Nutrition, Federal University of Mato Grosso do Sul (UFMS), Campo Grande 79070-900, MS, Brazil; danielletfreire@gmail.com; 2 Department of Pharmacy, Federal University of Rio Grande do Norte (UFRN), Natal 59078-970, RN, Brazil; 3 Cibles Thérapeutiques et Conception de Médicaments (CiTCoM), Unité Mixte de Recherche (UMR) 8038, Centre National de la Recherche Scientifique CNRS, Faculté de Pharmacie de Paris, Université Paris Cité, 75006 Paris, France; thalisson.amorim-de-souza@u-pariscite.fr

**Keywords:** natural products, medicinal plants, intellectual property, Brazilian wetlands, patent review

## Abstract

Pantanal is the largest wetland on the American continent. This biome contains several plant species of interest for the development of medicines and cosmetics. Hence, this work aimed to review technologies described in patents concerning highly published plants from the Pantanal and their pharmacological and cosmetic applications. This research surveyed species in the Brazilian Pantanal and identified those with relevant scientific data. Patent searches for the selected species were conducted covering the past 10 years. Overall, 1859 plant and fungal species from the Pantanal were identified. *Anacardium occidentale* L., *Annona montana* Macfad., *Annona muricata* L., *Annona squamosa* L., and *Calotropis procera* (Aiton) W.T. Aiton presented the highest number of scientific publications. The patent search yielded 58 patents, mostly filed by private companies and in South Korea. Additionally, extracts were the primary products examined. The main pharmacological activities exploited were antitumoral, anti-inflammatory, and antidiabetic. For cosmetic compositions, the main activities were anti-aging, anti-acne, hair loss, and skin whitening. Despite strong scientific evidence for the included plant species, their claimed activities, and filings from Countries with a strong history of technology development, most studies focused on extracts. Therefore, the development of formulations to deliver these plant extracts is encouraged to improve technology transfer.

## 1. Introduction

**Figure 1:**
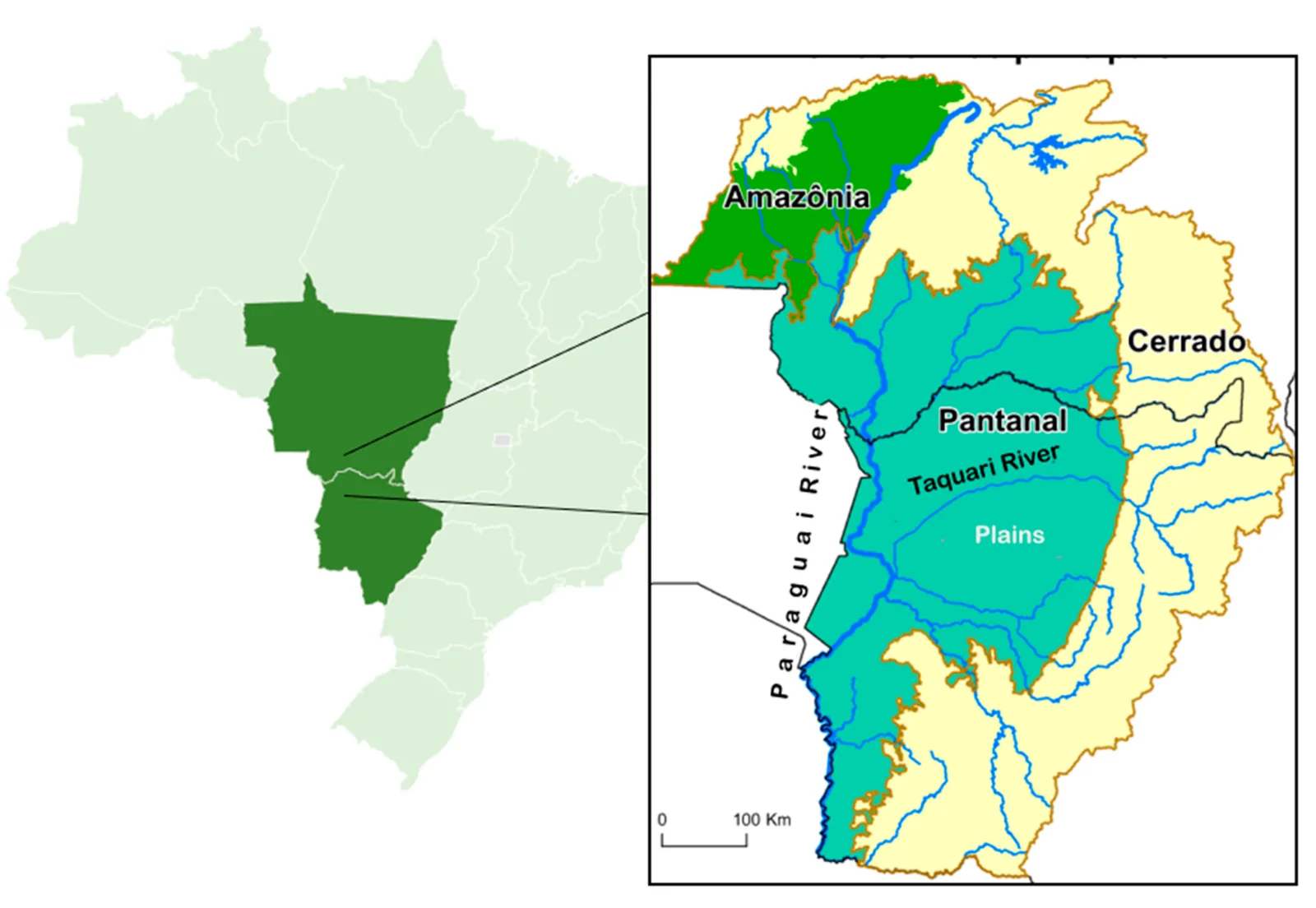
Pantanal biome and its location in Brazil.

Pantanal is a wetland located in the Brazilian states of Mato Grosso (MT) and Mato Grosso do Sul (MS). With an area of 140,000 km^2^, 35.36% in MT and 64.64% in MS, it is the largest wetland in America. Figure 1 presents this biome and its location in Brazil [1]. Given its extension and the importance of this biome in local folk medicine, the Pantanal is a valuable source of potential compounds of technological interest, as its rich biodiversity is underexploited for the discovery of new pharmaceutical and cosmetic ingredients [2].

These products offer faster alternatives for the treatment of several conditions and cosmetic applications, as the development and design of new synthetic drugs are onerous and slow processes [3]. From this perspective, natural products, mainly plants, are attractive to the pharmaceutical field, and their widespread use and ethnopharmacological studies support their investigation and use, guiding the triage of new molecular candidates for these products [4].

Due to their diverse characteristics and unique biofunctions, natural products represent an important source for new pharmaceutical and cosmetic products [5]. The development of plant-based products offers an efficient, lower-cost alternative compared to synthetic active pharmaceutical ingredients (APIs). Similarly, the use of natural products in cosmetic applications has gained traction over time, with the rise of the “green wave” and sustainable cosmetic products, which has increased demand for extracts and other natural products [6].

In Brazil, Law No. 13,123/2015 regulates access to genetic heritage and associated traditional knowledge, as well as benefit-sharing requirements when applicable, and the National System for the Management of Genetic Heritage and Associated Traditional Knowledge (SisGen) manages these requirements. These requirements are particularly relevant to research and technological development involving Brazilian biodiversity [7].

Whilst scientific trends are gaining strength through the publication of articles on plants found in the Pantanal, other documents, such as patents, receive insufficient attention. Patents are a reliable source of information on the state of the art in product production. This type of information is not always available in other scientific documents, such as articles [8].

Despite the relevance of these documents, there is a gap among scientific publications, patent filings, and the development of market-ready technologies. This disconnect underscores the strategic importance of patent landscaping as a tool for identifying technological trends, evaluating innovation potential, and bridging the divide between research and industrial application [9].

The analysis of patents through bibliometric methods is a powerful tool for identifying market trends, monitoring research and development activities, and forecasting emerging technologies. It also enables the identification of gaps in product development and the proposal of strategies to address them, thereby offering a comprehensive overview of a given field of knowledge [10].

Patent landscape analysis is a strategy to reduce uncertainty and support decision-making. These studies also provide guidance on future trends, analyze the market, and list the knowledge explored by companies and universities, thereby helping developers and stakeholders to make more informed decisions [11]. In this scenario, providing this information can prevent duplicate work and reduce costs for works already published.

Despite growing evidence of the potential of Pantanal plant species, little attention has been given to how scientific knowledge is translated into protected technologies. By mapping the most studied species occurring in the Pantanal and assessing their pharmacological potential, it is possible to address a global health challenge for the pharmaceutical and cosmetic industries by identifying innovation trends, technological gaps, and opportunities for drug discovery. In this sense, mapping these technologies can extend information beyond regional biodiversity and contribute to the global pharmacology and natural product-derived information landscape.

Considering this perspective, this study aimed to conduct a technological prospecting of Pantanal plants, focusing on their medicinal and cosmetic applications.

## 2. Materials and Methods

### 2.1. Survey for the Pantanal Species

A search was conducted in the Brazilian Flora and Funga database (https://reflora.jbrj.gov.br/consulta/, accessed on 1 September 2025) to identify the species found in the Pantanal biome [12]. The species were filtered by geographic range, retaining those with any occurrences in Brazil, including all endemics and all origins (native, cultivated, or naturalized). The central-west region and any state within the Pantanal’s phytogeographic domain were chosen. In this sense, only the Brazilian Pantanal was used in this search, as the selected database lacks data on adjacent Countries.

Subsequently, a PubMed search was conducted to identify species with relevant scientific data on the plants. This approach was used to select the most studied species found in the Pantanal. Although this strategy may introduce bias and does not represent the full ecological potential of the Pantanal; this approach was selected as it provides a measurable indicator, ensuring that selection does not depend on subjective criteria. Furthermore, more studied species tend to have a larger volume of experimental data, allowing for more robust analyses and more consistent comparisons.

The search was conducted using the advanced search builder, with each plant’s scientific name and known synonyms as keywords. The search was restricted to the title and abstract fields. To ensure that only pharmacologically relevant species were retrieved, the keywords “bioactivity”, OR “medicinal”, OR “biological activity”, OR “phytochemistry” were included. In addition, the keyword “review” and the descriptor “not” were used in the “publication type” field. The publication years were limited to 2015–2025. The full search prompt model and an example with one of the species are provided in the Supplementary Materials (Prompt S1 and Prompt S2).

### 2.2. Patent Search and Data Treatment

The five species with the most scientific publications on PubMed were selected for screening in the World Intellectual Property Organization (WIPO) and the European Patent Office (Espacenet) databases. The search strategy was carried out using the following combination:

- “*Annona* AND *montana*” OR “Araticum” OR “Mountain AND soursop”;
- “*Annona* AND *muricata*” OR “Graviola” OR “Soursop”;
- “*Annona* AND *squamosa*” OR “Sweetsop” OR “Pinha”;
- “*Calotropis* AND *procera*” OR “Apple AND of AND Sodom” OR “Algodão AND de AND Seda”;
- “*Anacardium* AND *occidentale*” OR “Cashew” OR “Caju”;

AND

- The International Patent Classification (IPC) codes A61K (Preparations for medical, dental, or toiletry use) or A61Q (Specific use of cosmetics or similar toiletries).

The use of WIPO and Espacenet can be justified as these databases hold patent documents from several countries. For instance, Espacenet is a patent search engine that holds 140 million patents submitted to more than 90 patent offices worldwide, including Brazil [11].

The IPC codes A61K and A61Q were chosen to prioritize documents related to pharmaceutical and cosmetic formulations. The approach used in this study sought to preserve methodological consistency and support a focused qualitative interpretation of the study’s object, minimizing technological heterogeneity and ensuring a more coherent assessment within the exploratory scope of this review [13].

Given the nature of this study, as an exploratory and qualitative review rather than a scoping or systematic review, the screening and data extraction were conducted by a single reviewer. This approach was considered appropriate due to the study’s objectives, which focused on mapping technologies and identifying trends.

The patent search was conducted in the WIPO and EPO databases, using the title and abstract fields, considering patents published from 2015 to 2025 in any language. This criterion was adopted to ensure that the recovered documents were recent, so that the patents returned in the search could be analyzed for their potential to advance to higher maturity levels. Duplicates were assessed using publication, application, and priority numbers. When multiple publication documents referred to the same invention, they were treated as a single record, and the analysis retained the granted publication as the representative document. Patent records with distinct priority numbers were considered separate inventions. Patents published before 2015 and patents not granted were excluded during screening or eligibility assessment. The restriction to granted patents was adopted to provide a more consolidated set of patent documents for this exploratory analysis. Patent grant was not interpreted as evidence of scientific efficacy, technological maturity, or commercial readiness. We acknowledge that this criterion may underrepresent more recent technologies with pending applications and therefore represents a limitation of the present study. Patents were analyzed according to the inclusion criteria: those that contained the species of interest and targeted pharmacological or cosmetic activities. The search was conducted in September 2025 and adhered to the PRISMA guidelines for search and screening to improve reproducibility [14].

The following information was extracted from the eligible patents: year of publication, country, type of applicant, technologies, and biological activities. To ensure consistent country assignment, we used the applicant’s country as the primary criterion, as it best reflects the invention’s institutional origin. The information was gathered to generate graphs using Excel^®^ (Microsoft 365, Microsoft, Redmond, WA, USA) and GraphPad Prism version 8.0.0 for Windows (GraphPad Software, Boston, MA, USA).

## 3. Results

Three primary sections, each representing a distinct stage of the technological prospection process, are used to present the results. The recognized species of the Pantanal biome were first surveyed and screened based on their documentation on PubMed, defined as the number of publications retrieved for each species (Section 3.1). Subsequently, filing patterns, applicant characteristics, and technological categories were used to characterize the patent documents obtained (Section 3.2). A brief phytochemical summary on the included species was provided (Section 3.3). Lastly, the pharmacological and cosmetic claims in the patents were thoroughly examined (Section 3.4 and Section 3.5), with a focus on the compounds’ and extracts’ biological activities and potential uses.

### 3.1. Survey of the Species

The search for all Pantanal species in the Midwest region of Brazil yielded 1859 species (both plants and fungi). After screening the PubMed database, 303 plant species were identified in 3636 papers published over the past 10 years. Table S1 in the Supplementary Material lists all species identified in the PubMed search, along with widely known synonyms (Table S2).

**Figure 2:**
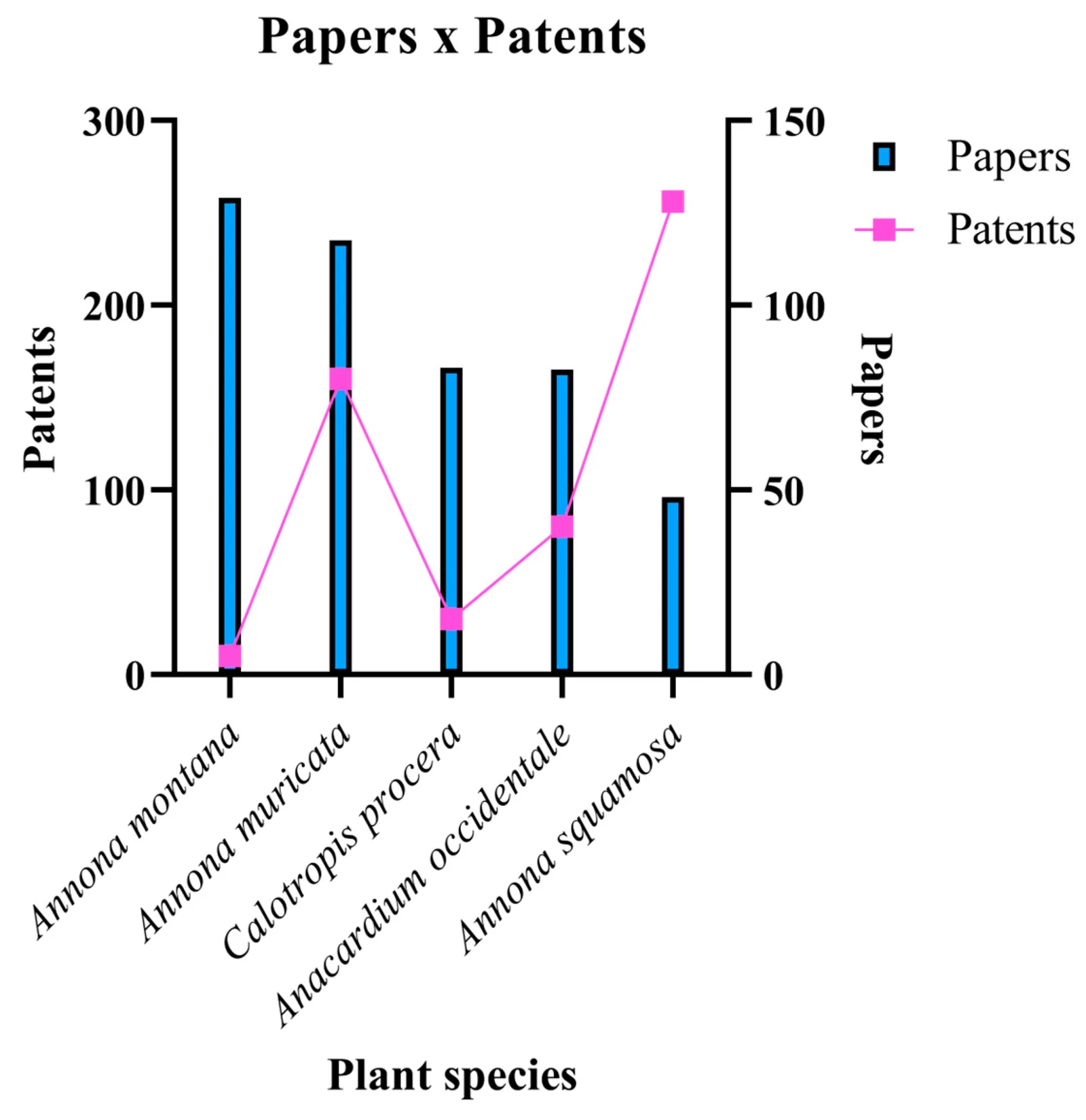
Number of scientific articles (blue bars) and patents (pink line) published in the databases. Letter “x” means “versus”.

The five species with the highest number of published articles were: *Anacardium occidentale* L., *Annona montana* Macfad., *Annona muricata* L., *Annona squamosa* L., and *Calotropis procera* (Aiton) W.T. Aiton. In Figure 2, the number of publications, including papers and patents, available in the databases is shown as a cross-link comparison.

In general, the number of articles did not correspond to the number of patent deposits; this discrepancy may be attributed to the nature of these documents. An experimental scientific publication reports a discovery or its potential validation. A patent document provides legal protection for an invention; the evident disparity between papers and patents highlights the long process researchers face in overcoming the bottleneck between discovery and the economic and legal protection of their inventions.

In this work, the 5 species selected for patent analysis are primarily from the families Anacardiaceae, Apocynaceae, and Annonaceae. The prominence of Annonaceae is supported by the chemical diversity and strong biological activities of its compounds. The focus on these species stems from the presence of unique secondary metabolites, such as annonaceous acetogenins. These compounds are notable for their aliphatic chain structures and mechanisms of action. They exhibit high toxicity and antiproliferative activity against several cancer cell lines. The pursuit of new antitumor drugs makes the prospecting of natural products with such characteristics more promising for scientific purposes. In this scenario, the number of scientific publications aimed at isolating, characterizing, and evaluating the potential of these compounds is increasing.

Although the Annonaceae family stands out for the number of publications, the inclusion of species from the Anacardiaceae and Apocynaceae families is equally justified by their pharmacological relevance. The representative of the Anacardiaceae family, *A. occidentale*, is a plant rich in phenolic lipids, such as cardol, cardanol, and anacardic acids, which have well-documented pharmacological activities, including antimicrobial, antioxidant, and anti-inflammatory effects. Similarly, the representative of the Apocynaceae, *C. procera*, contains calotoxin, calotropagenin, and calotropin, among others, which belong to the cardenolide class of steroidal glycosides, characterized by a steroid nucleus linked to an unsaturated butenolide ring exhibiting anti-inflammatory, antioxidant, and antimicrobial activities [15].

### 3.2. General Description of the Patents

**Figure 3:**
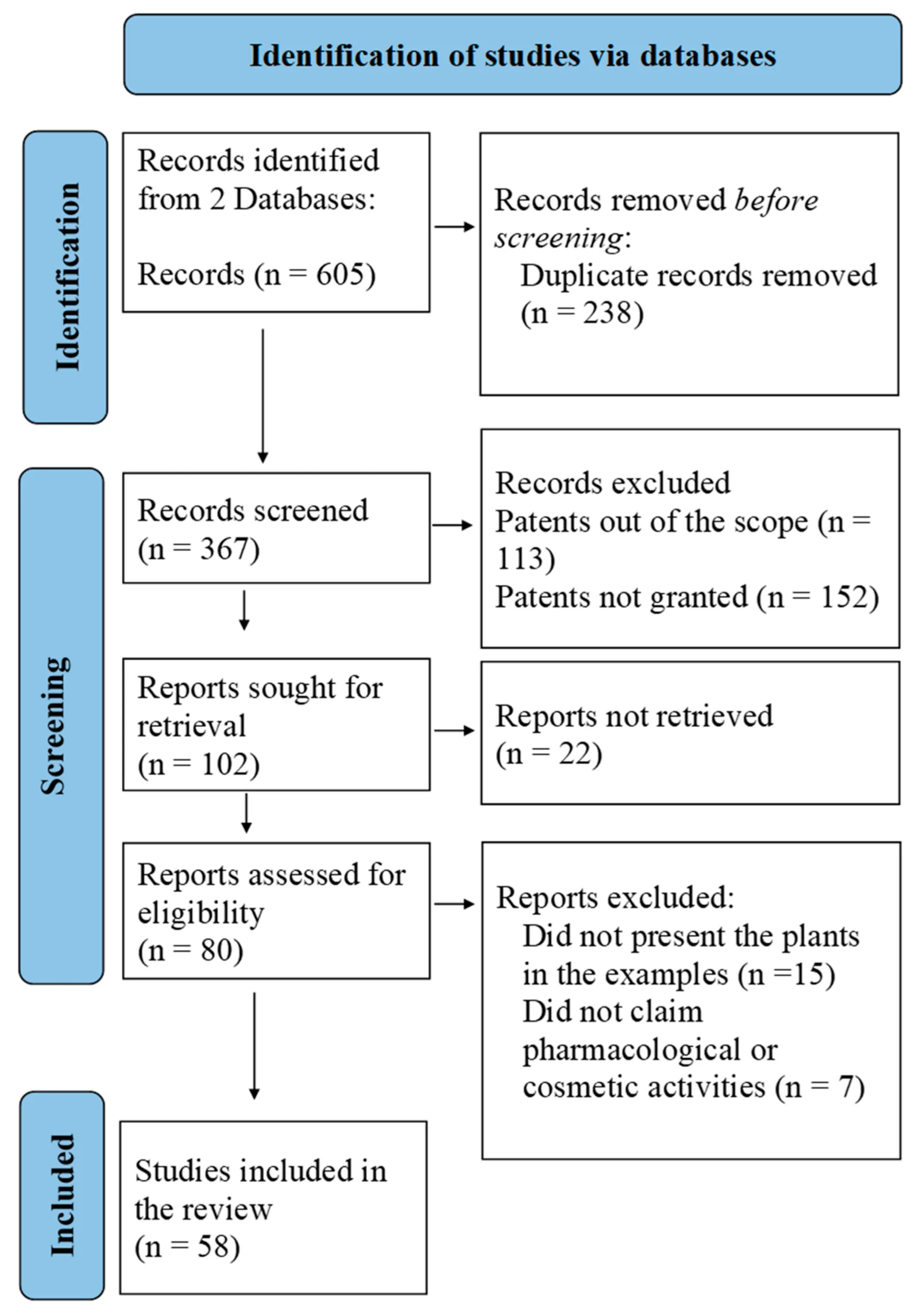
Flowchart of the search on patent databases.

The patent search for these 5 species resulted in 605 documents. Among these, 238 duplicates were excluded. Hence, 367 documents were screened, and the titles and abstracts were read. As a result, 113 were excluded because they did not fall within the study’s scope, and 152 patents were not granted. In this context, 102 patents were sought for retrieval, of which 22 were not retrievable; thus, 80 documents were assessed for eligibility. Finally, 58 patents regarding the selected species were included in this review. The detailed flow diagram of the search is presented in Figure 3. Table S3 of the Supplementary Materials provides the dataset of the patents included in the technological prospection, including the bibliographic and patent classification data extracted from each document.

**Figure 4:**
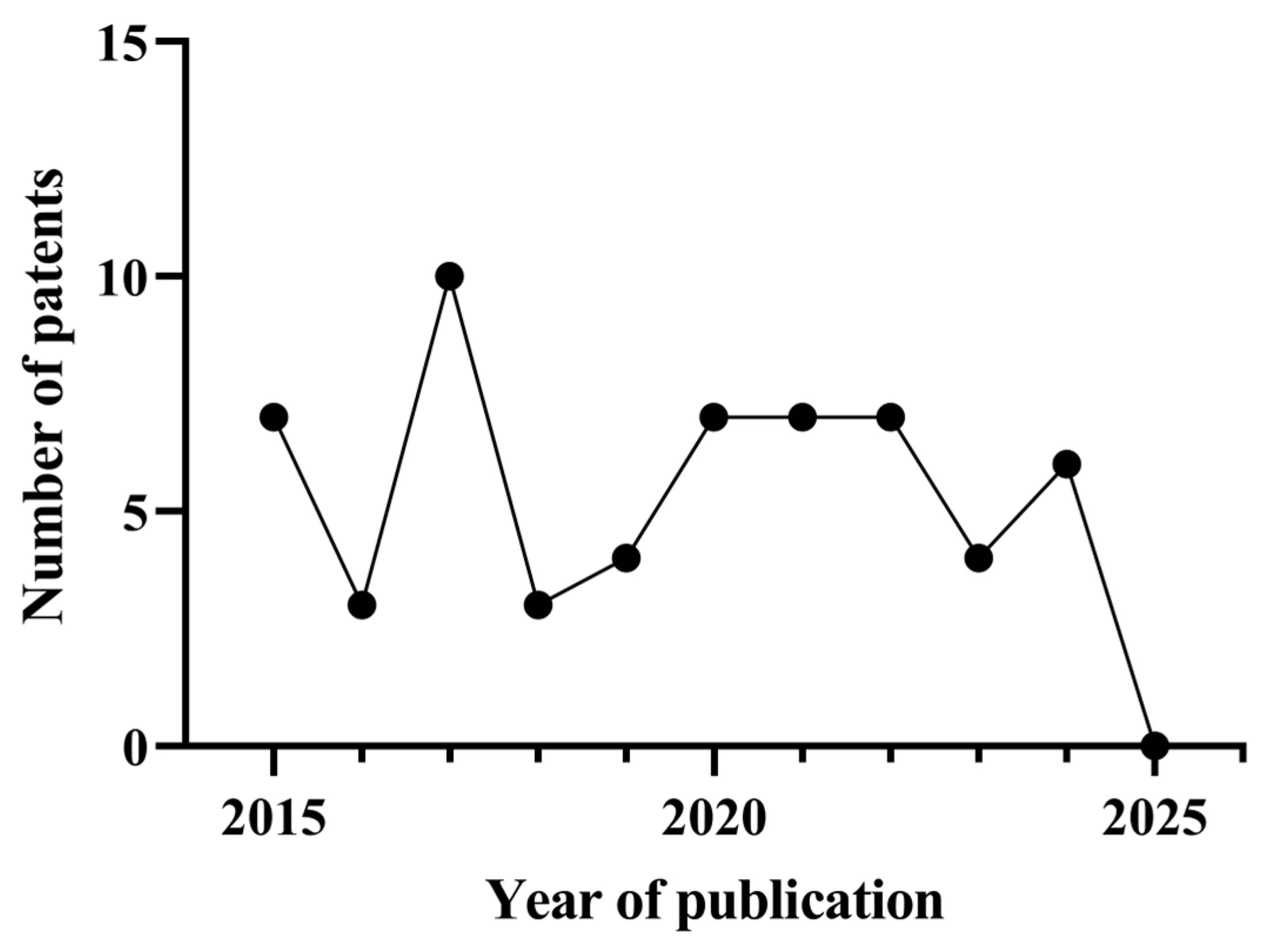
Annual profile of patent publications.

Among the included patents, the first report was “BR102013012924—LCC amin derivatives as acetylcholinesterase inhibitors, processes for obtaining them, pharmaceutical compositions containing them and applications”, which concerned extraction methods for amin derivatives from the nut liquid of *A. occidentale* and their uses. The number of patent applications involving Pantanal plants has increased, with the highest number of publications occurring in 2017 (10 patents), as shown in Figure 4. Following 2015, there was an increase in public policies promoting innovation and biodiversity, creating a more favorable environment for bioprospecting. Moreover, a decline in patent publications is noted after this period. This decline may be due to some key factors, such as funding limitations, regulatory complexities related to genetic resources, and challenges in advancing technological maturity; however, it is only possible to infer the role of these factors in the reduction in technologies after this period.

**Figure 5:**
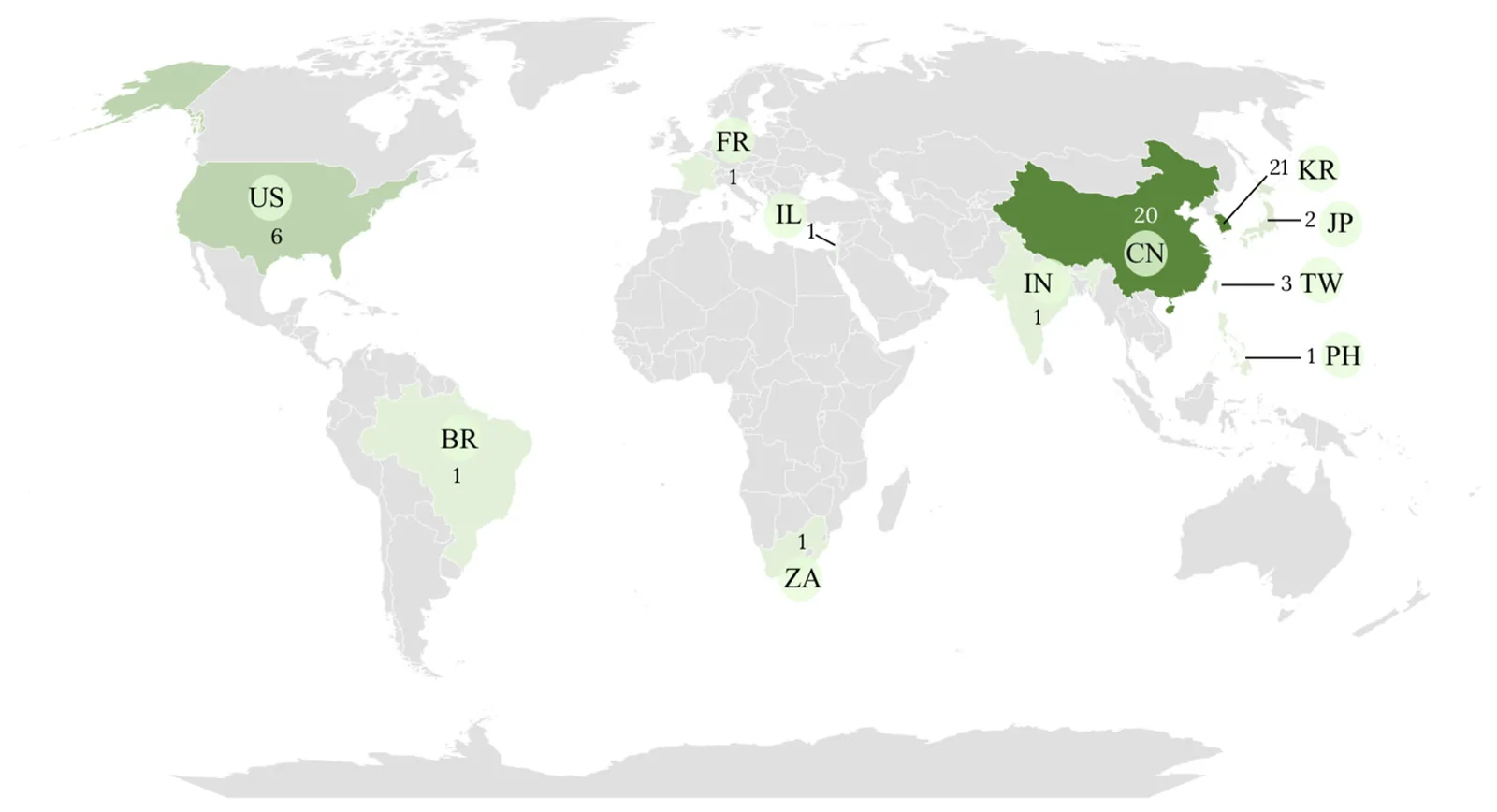
Map of the countries with deposited patents. (Dark green: the highest number of patents; Light green: the least number of patents. US: United States of America; BR: Brazil; FR: France; ZA: South Africa; IL: Israel; IN: India; CN: China; KR: South Korea; JP: Japan; TW: Taiwan; PH: Philippines).

From the documents analyzed, the major holder of inventions is South Korea, with 21 patents granted, followed by China with 20 patents, the United States with 6 patents, Taiwan with 3 patents, Japan with 2 patents, and lastly France, India, Brazil, South Africa, the Philippines, and Israel with 1 patent each, as seen in Figure 5.

This can be explained by the aggressive policies adopted by South Korea and China to promote innovation and protect intellectual property. Government initiatives such as Made in China 2025 and the K-Bio strategy have advanced innovation in these Asian countries, particularly in biotechnology [16,17]. This also accounts for the significant increase in innovations in 2017, following the launch of these programs after 2015. From the selected species described in this work, both China and South Korea have related technologies regarding the application of *A. occidentale*, *A. muricata*, and *A. squamosa*. Among all patents filed by inventors from these countries, only three documents stated the source from which the species were obtained (KR101868507—graviola powder from Yeongcheon, Gyeongbuk; KR101951854—graviola leaves from the Philippines; and US9533019—samples collected during the flowering season from Al-Yamamah territory). This lack of information significantly limits the potential for reproducible biological activity, given the likelihood of phytochemical variation.

Although the described species occur in the Pantanal, none are endemic to Brazil. From the plants described in this review, *A. occidentale* is the only native species; *A. montana* and *C. procera* are naturalized, and *A. muricata* and *A. squamosa* are cultivated species [12]. The fact that these species are not exclusive to Brazil may justify their use in patent applications in other countries, enabling bioprospecting across multiple jurisdictions. In Brazil, research and technological development involving access to Brazilian genetic heritage or associated traditional knowledge must comply with the applicable requirements of Law No. 13,123/2015, including registration in SisGen prior to requesting intellectual property rights, when applicable [7].

**Figure 6:**
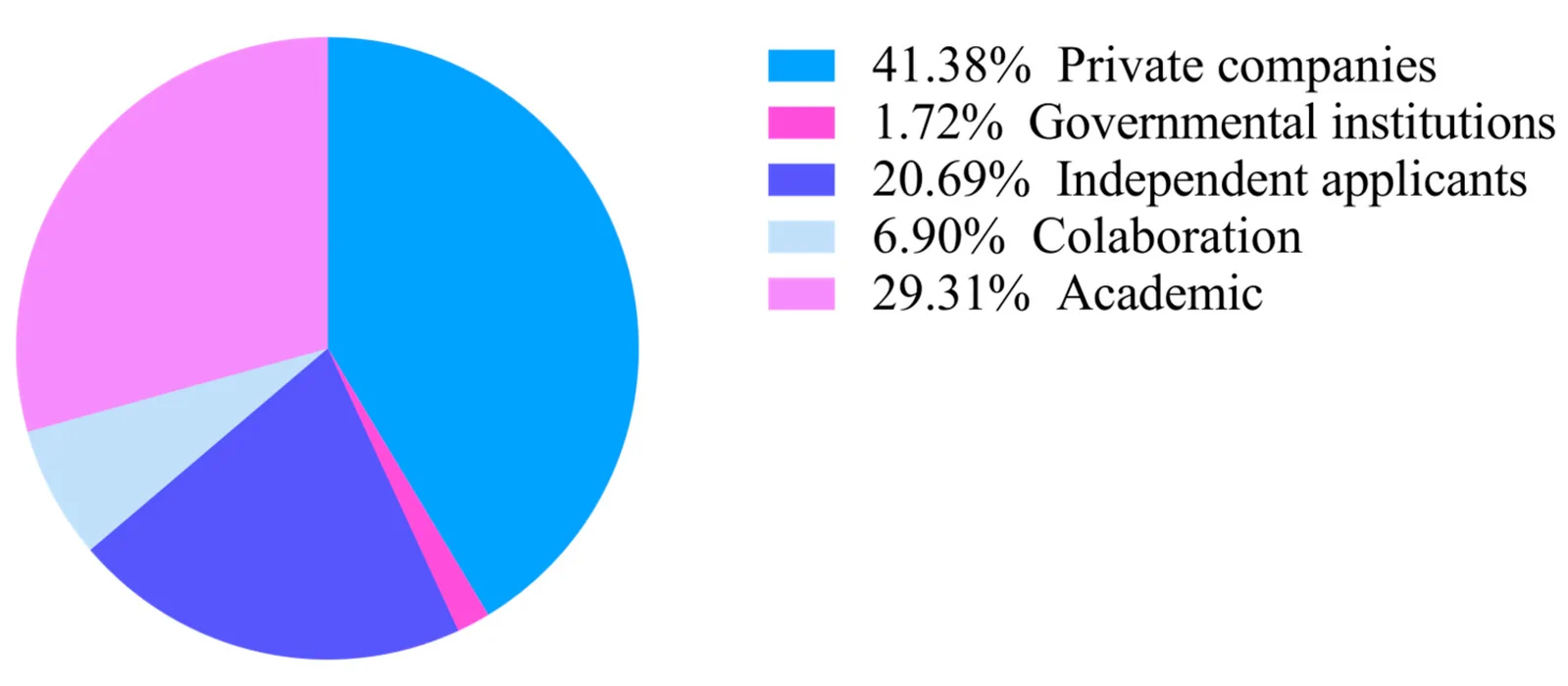
Applicants’ distribution by organization types.

Among the applicants for the described patents, most documents (41.38%) are held by private organizations, as shown in Figure 6. This is a common scenario once the scientific development of patented products aligns with commercial manufacturing [18]. Furthermore, research and development (R&D) in universities face insufficient levels of technology maturity since it focuses mostly on basic research; thus, collaborating with the private sector is an important step to put forward academic R&D. Notwithstanding, the academic initiative represents 29.31% of the patented documents, 20.69% of the documents are held by independent applicants and 6.9% of the documents come from a collaboration between public and private initiatives.

**Figure 7:**
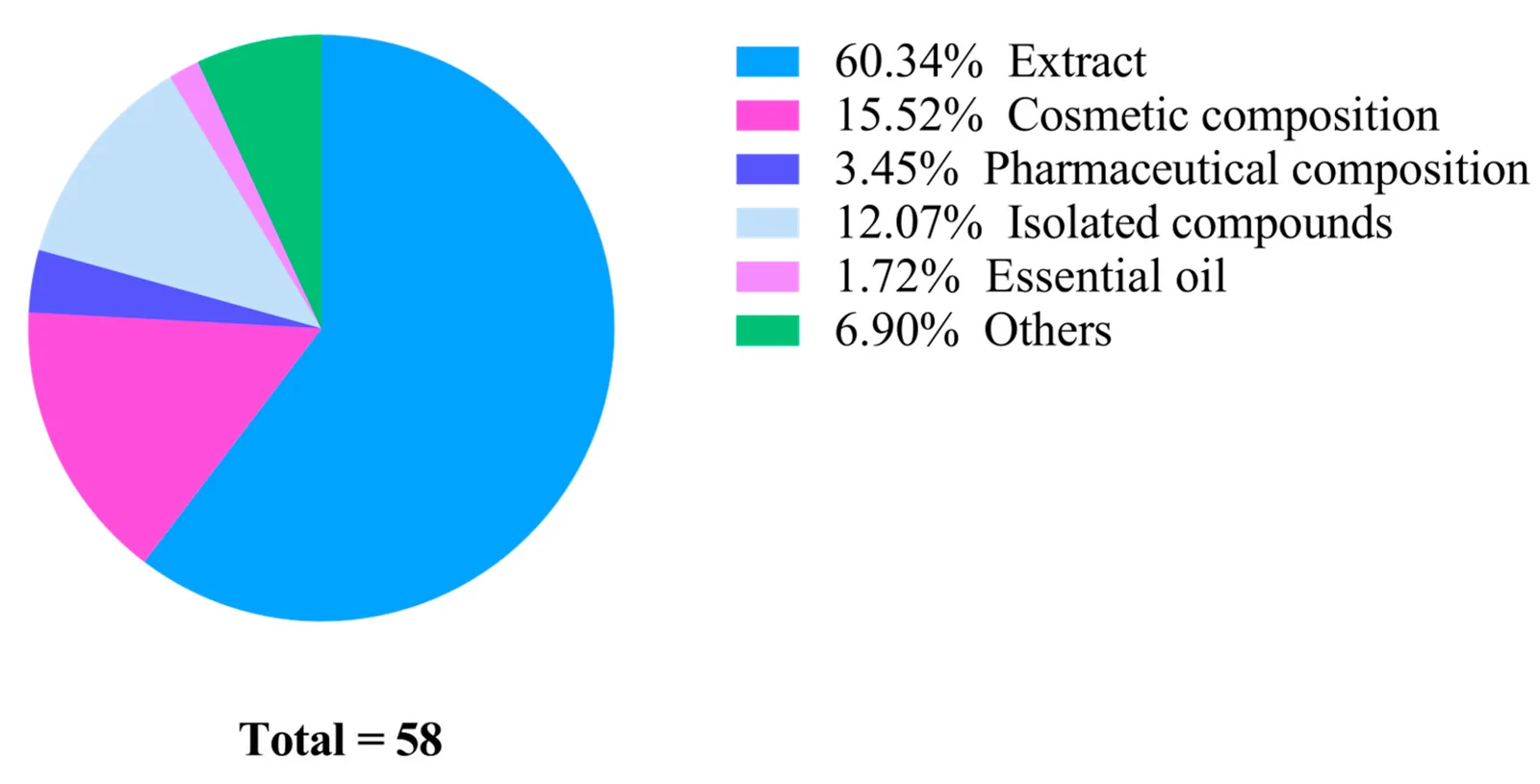
Main claimed technologies concerning species found in the Pantanal.

Regarding the technologies present in these documents, 60.34% related to the methods and uses of extracts of the studied plants, followed by cosmetic compositions with 15.52% of the total of the patents, 12.07% relate to isolated compounds, 3.45% describe pharmaceutical compositions, 1.72% relate to the use of essential oil, and 6.9% are related to other types of formulations, such as beverages. Figure 7 displays the main claimed technologies found in the patents.

The predominance of extracts is common in natural product innovation, as these intermediate products are chosen for their simplicity, broad applicability across multiple biological activities, and less stringent regulatory requirements compared with isolated compounds. Natural products can be patented in different offices, subject to specific criteria. For instance, in Brazil, China, Korea, and Europe, patenting of natural products may concern only the methods and uses of the genetic source; in the United States, patenting of genetic resources is permitted.

In this sense, a technological limitation is evident, as these inventions remain at an early stage of development, thereby limiting the options for complex formulations and administration systems that could enhance stability, bioavailability, and efficacy. On the other hand, the small number of patents for isolated molecules suggests limited investment in R&D and subsequent formulation. This reality highlights the challenge of advancing biodiversity-based products to higher maturity levels.

Within the A61K and A61Q IPCs, subclassifications A61K36 and A61Q19 were the most common in the patents. While A61K36 describes medicinal preparations of undetermined composition materials from algae, lichens, fungi, or plants, A61Q19 refers to cosmetic or hygiene formulations for use on the skin. Other subclassifications, such as A61K8 and A61Q5, also appeared, as they specified product types and uses within the IPC code. It is worth mentioning that other unrelated codes were simultaneously claimed in the patents, likely to expand use or market interest in the fields of chemistry and nutrition, including A23L33 and C07.

Among the patented compositions, cosmetic formulations accounted for 9 patents. The applicability of extracts from plants found in the Pantanal spans cosmetic products, including shampoos (3 patents), nanoemulsions, niosomes, facial masks, gels, conditioning essences, and hair dye compositions (1 patent each). The range of bioactive compounds and biological activities for cosmetic purposes makes this type of formulation prevalent; in addition, the easier regulatory pathway for cosmetic products facilitates its development. The use of delivery systems such as nanoemulsions and niosomes underscores the inventors’ interest in developing formulations that enhance stability, efficacy, and sensory experience of topical products.

On the other hand, pharmaceutical compositions represent only 2 patents: 1 cream and 1 nanoparticle. Creams are well-established pharmaceutical dosage forms that offer advantages over extracts, such as improved sensory properties and a tunable release profile, despite their simple composition. Furthermore, the development of nanoparticles, particularly in formulations, enables the delivery of antitumor agents and demonstrates the potential to develop robust pharmaceutical dosage forms that support sustained bioactive release, thereby reducing side effects associated with the active ingredient. Furthermore, the use of nanoparticles enables site-specific delivery, a desirable characteristic for antitumor agents. Despite this, the limited number of pharmaceutical formulations indicates that the technological readiness level (TRL) of inventions concerning plants found in the Pantanal remains in the initial phases, i.e., discovery and proof-of-concept (TRLs 1–3). The TRL framework was adopted to assess the technological maturity of the patents according to the type of evidence reported on the documents, were TRL 1–2 corresponded to basic research or in vitro data; TRL 3–4 to proof-of-concept and laboratory validation; TRL 5–6 to validation in relevant environments, including animal studies or early human trials; TRL 7–8 to clinical trials or advanced prototypes; and TRL 9 to commercially available products.

### 3.3. Summary of the Phytochemical Composition of the Selected Species

The analyzed patents reported the use of five plant species for a range of biological activities. Plants produce secondary metabolites, including terpenes and phenolic compounds, that contribute to their adaptation to environmental challenges. Some of these substances may also provide benefits to human health. Therefore, characterizing the phytochemical composition of these species is essential for understanding their reported biological activities.

**Figure 8:**
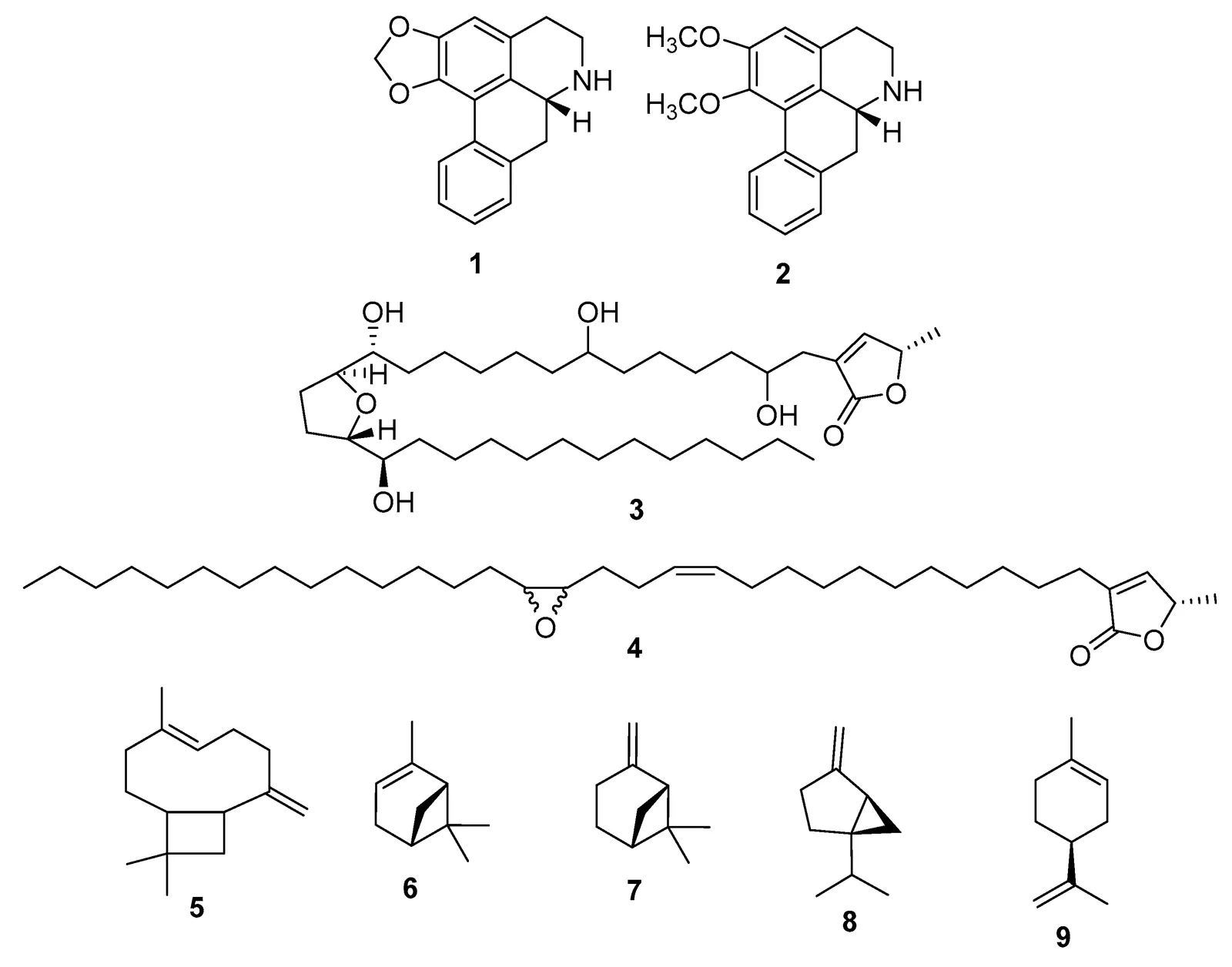
Some representative compounds reported in these three Annonaceae species. All compound numbers are mentioned in the main text with their chemical names.

**Figure 9:**
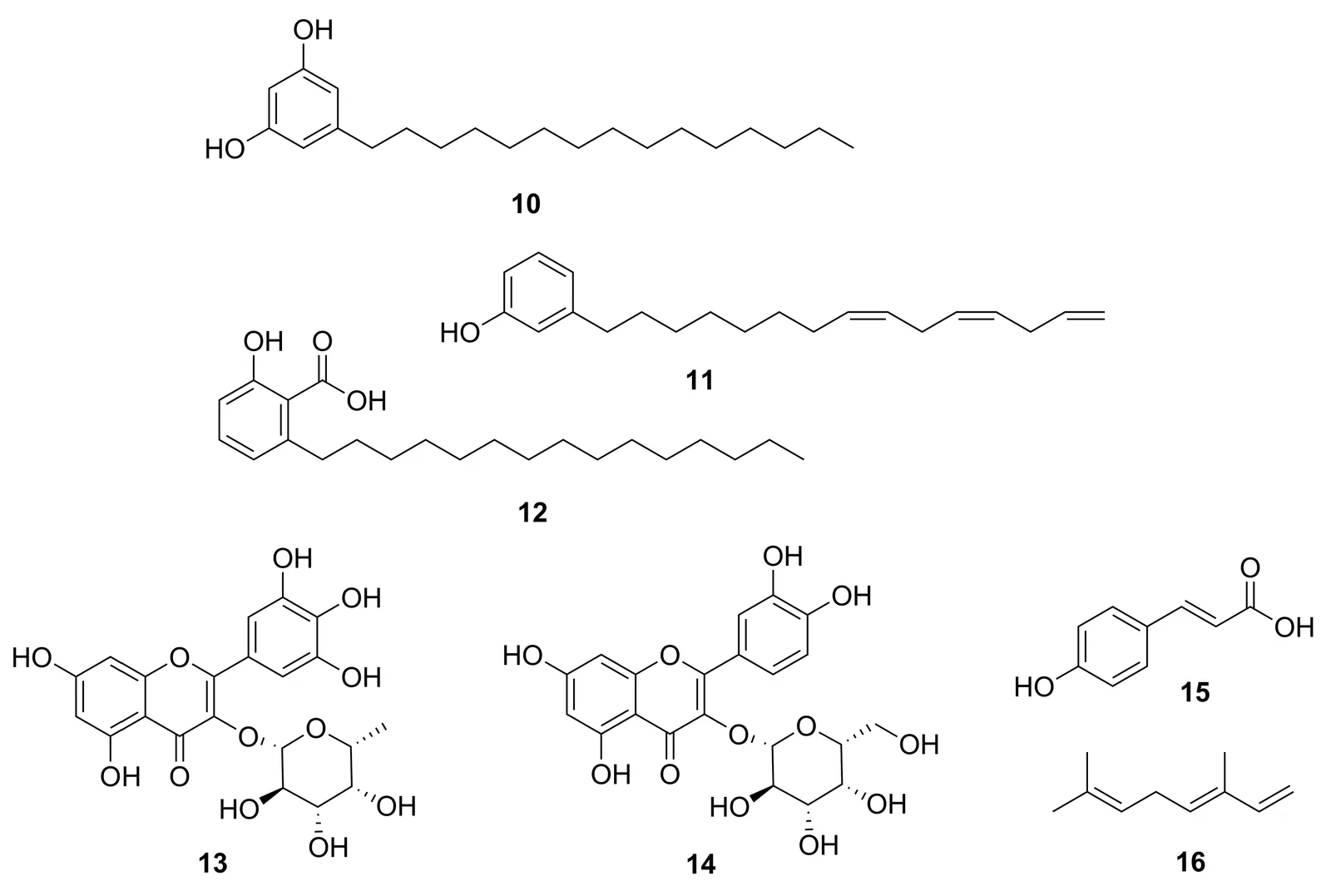
Some representative compounds reported in *Anacardium occidentale.* All compound numbers are mentioned in the main text with their chemical names.

**Figure 10:**
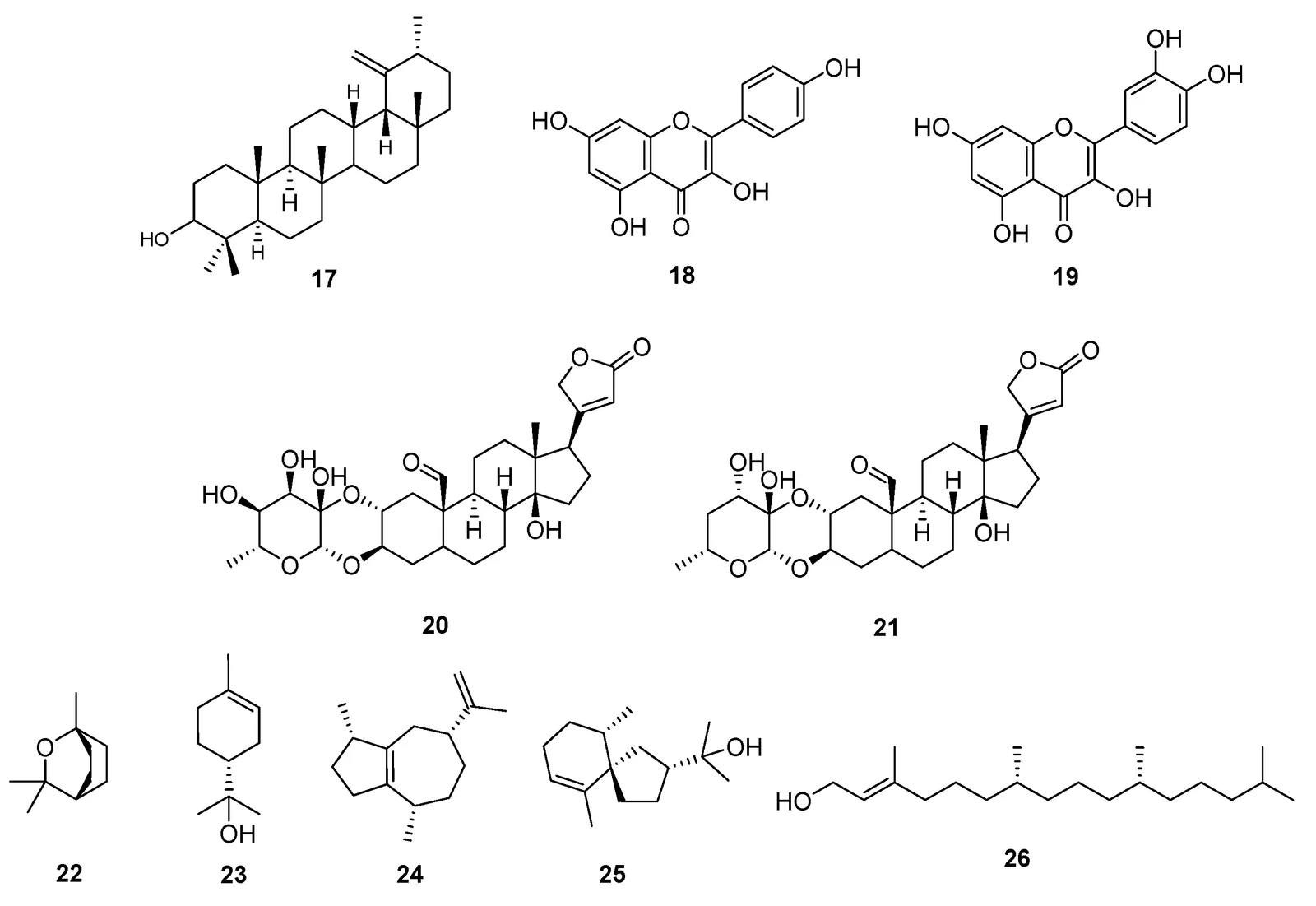
Some representative compounds reported in *Calotropis procera.* All compound numbers are mentioned in the main text with their chemical names.

The increasing use of dereplication techniques, particularly HPLC–MS, has made chemical profiling of plant extracts and other complex samples a key step in species prioritization and in identifying chemical markers for pharmaceutical development [19,20]. In this context, the combination of classical and modern analytical techniques has enabled the identification of numerous potentially bioactive compounds, ranging from essential oil constituents to non-volatile metabolites across different chemical classes. This section summarizes the main phytochemical representatives reported in the literature for the five selected species, thereby supporting the discussion of the technologies described in the patents. Information on the phytochemicals addressed in this section is provided in the Supplementary Material (Table S4). Additional supporting references beyond those shown in the article were also used to help draw chemical structures from Figure 8, Figure 9 and Figure 10, as shown in the Supplementary Material for conciseness.

Annonaceae species contain a wide range of secondary metabolites, including flavonoids, alkaloids, and volatile compounds, such as monoterpenoids, sesquiterpenoids, phenylpropenoids, and small aliphatic molecules, mainly found as constituents of essential oils. These plants have a long history of use as food and medicine, and some species are economically important crops in Asia and the Americas [21]. A recent study based on the analysis of herbarium specimens indicated that aporphine alkaloids and acetogenins are major chemical markers of several Annonaceae genera [22].

Aporphine alkaloids are formed through the cyclization of isoquinoline precursors [23]. Their main structural core comprises four fused six-membered rings. Anonaine (**1**) and nornuciferine (**2**) are frequently described in Annonaceae species. Acetogenins are polyketide-derived metabolites characterized by long aliphatic chains containing one or more tetrahydrofuran rings and a terminal α,β-unsaturated γ-lactone moiety [3], as observed in annoreticuin (**3**) and sabadelin (**4**) [24].

Despite their structural differences and interactions with distinct molecular targets, Annonaceae alkaloids and acetogenins exhibit several shared biological activities, including cytotoxic, anti-inflammatory, and antibacterial effects. Some of these properties may be useful for the development of cosmetic products and active pharmaceutical ingredients. Both classes have been reported in *A. montana*, *A. muricata*, and *A. squamosa* [25].

Essential oils are widely used in the development of nanostructured systems for therapeutic and cosmetic applications. Essential oils obtained from the leaves and fruits of *A. muricata* and *A. squamosa* are rich in sesquiterpenes, particularly β-caryophyllene (**5**) [26]. Compounds belonging to this class have been extensively investigated. Their reported properties include anti-inflammatory activity and potential applications against Streptococcus infections. Analgesic, myorelaxant, and sedative effects have also been described [27,28]. Monoterpenes are also present, with α-pinene (**6**), β-pinene (**7**), sabinene (**8**), and limonene (**9**) frequently reported [26]. In contrast, studies on the essential-oil composition of *A. montana* remain scarce, highlighting the need for further phytochemical investigation of this species. Figure 8 depicts the structure of the compounds described in Annonaceae.

Anacardiaceae includes important sources of edible fruits and condiments, such as mango (*Mangifera indica*), pistachios (*Pistacia* spp.), and pink peppercorns (*Schinus terebinthifolia*). In Brazil, one of the best-known representatives of this family is cashew (*A. occidentale*) [29]. The cashew nut and pulp, corresponding to the true fruit and pseudofruit, respectively, are used in traditional recipes and as raw materials for the food industry.

From a phytochemical perspective, the fruit and pseudofruit have distinct chemical compositions. Cashew nuts are rich in phenolic lipids, particularly cardol (**10**), cardanol (**11**), and anacardic acid (**12**), which are major constituents of cashew nutshell liquid. In contrast, cashew pulp is rich in phenolic compounds, including flavonoids, anthocyanins, catechins and their derivatives, and carotenoid esters. Among these constituents, glycosylated flavonoids such as myricetin-3-*O*-rhamnoside (**13**) and quercetin-3-*O*-galactoside (**14**), as well as phenolic acids such as *p*-coumaric acid (**15**), can be highlighted [30].

Structurally, phenolic lipids are characterized by aromatic rings bearing hydroxyl or carboxylic acid groups and long alkyl or alkenyl side chains. These compounds exhibit anti-inflammatory, antifungal, antiparasitic, and antibacterial activities [31]. In contrast, synthesized from the combination of the phenylpropanoid and polyketide pathways, flavonoids are characterized by a C_15_ carbon skeleton arranged into three rings, consisting of two aromatic rings (A and C) and one pyran ring (B). Their structural diversity arises from differences in the oxidation pattern of these rings, giving rise to six major subclasses. To date, more than 6000 flavonoids have been identified [32]. Although most studies have focused on the chemical composition and applications of cashew fruits, targeted metabolomic analyses indicate that the leaves contain higher concentrations of flavonoids and other phenolic derivatives than the fruit pulp [33]. Cashew gum has also been investigated for several applications in nanostructured systems [34]. Taken together, these findings highlight *A. occidentale* as a versatile source of compounds and materials with potential technological applications.

Essential oils are also found in the aerial parts of *A. occidentale*. (*E*)-β-ocimene (**16**) is a representative volatile organic compound from this group [35]. It plays an important role in ecological interactions and has also been associated with several biological activities [36,37]. Fatty acids are the major constituents of the oil obtained from cashew pulp, with palmitic acid being one of its main components. Figure 9 depicts the structures of representative compounds reported in *A. occidentale*.

In addition to their abundance of bioactive compounds, these species are relevant to the food industry, particularly *A. squamosa*, *A. muricata*, and *A. occidentale*. The processing and utilization of various plant parts, including fruits, may promote the establishment of production chains and bioeconomic cycles that align with sustainable development and contemporary socio-environmental demands.

The Apocynaceae family is known for its chemical diversity, particularly its alkaloid content. However, several other classes of bioactive metabolites have also been reported in this plant group. These include terpene derivatives and steroidal glycosides, particularly pregnane glycosides and cardenolides [38]. The phytochemical profile reported for *C. procera* (Apocynaceae, subfamily Asclepiadoideae) revealed the presence of cardenolides, flavonoids, sterols, oxypregnanes, triterpenoids, glycosides, and other constituents [39].

Considering the rich chemical composition of this species, several representative compounds can be highlighted. Triterpenes, including calotropenol (**17**), are found predominantly in the latex and leaves. Flavonoids such as kaempferol (**18**) and quercetin (**19**), as well as their glycosylated derivatives, have been identified in the stems, leaves, and flowers. These compounds are widely recognized for their antioxidant properties. In addition, calotoxin (**20**) and calotropin (**21**) were among the first cardenolides described in this species [40]. Cardenolides are known for their high toxicity, which may initially appear to limit the use of *C. procera* as a potential raw material. However, other plants rich in this class of metabolites are used in cosmetic applications using extracts enriched in less-toxic constituents, including flavonoids. Examples include *Convallaria majalis* and milkweed species, which have also been explored as sources of fibers and essential oils; some of these uses were even supported by ethnopharmacological surveys [41,42].

From a pharmaceutical perspective, plants in the genus *Digitalis* played an important role in the discovery of cardiotonic agents. Moreover, semisynthetic cardenolide derivatives have exhibited additional biological properties that extend beyond their classical cardiotonic effects, including anti-inflammatory and antimicrobial activities [43]. These findings indicate that, when appropriate approaches are employed, *C. procera* may offer broad potential for pharmaceutical and cosmetic applications.

Concerning the essential oil of *C. procera*, CG-MS analysis revealed the presence of oxygenated monoterpenes and sesquiterpenes, including eucalyptol (**22**), terpineol (**23**), α-guaiene (**24**), hinesol (**25**), as well as phytol (**26**) and other constituents. This essential oil has been evaluated for its antimicrobial activity and allelopathic effects against weed species [44]. Figure 10 depicts the structures of representative compounds reported in *C. procera*.

### 3.4. Pharmacological Activities

**Figure 11:**
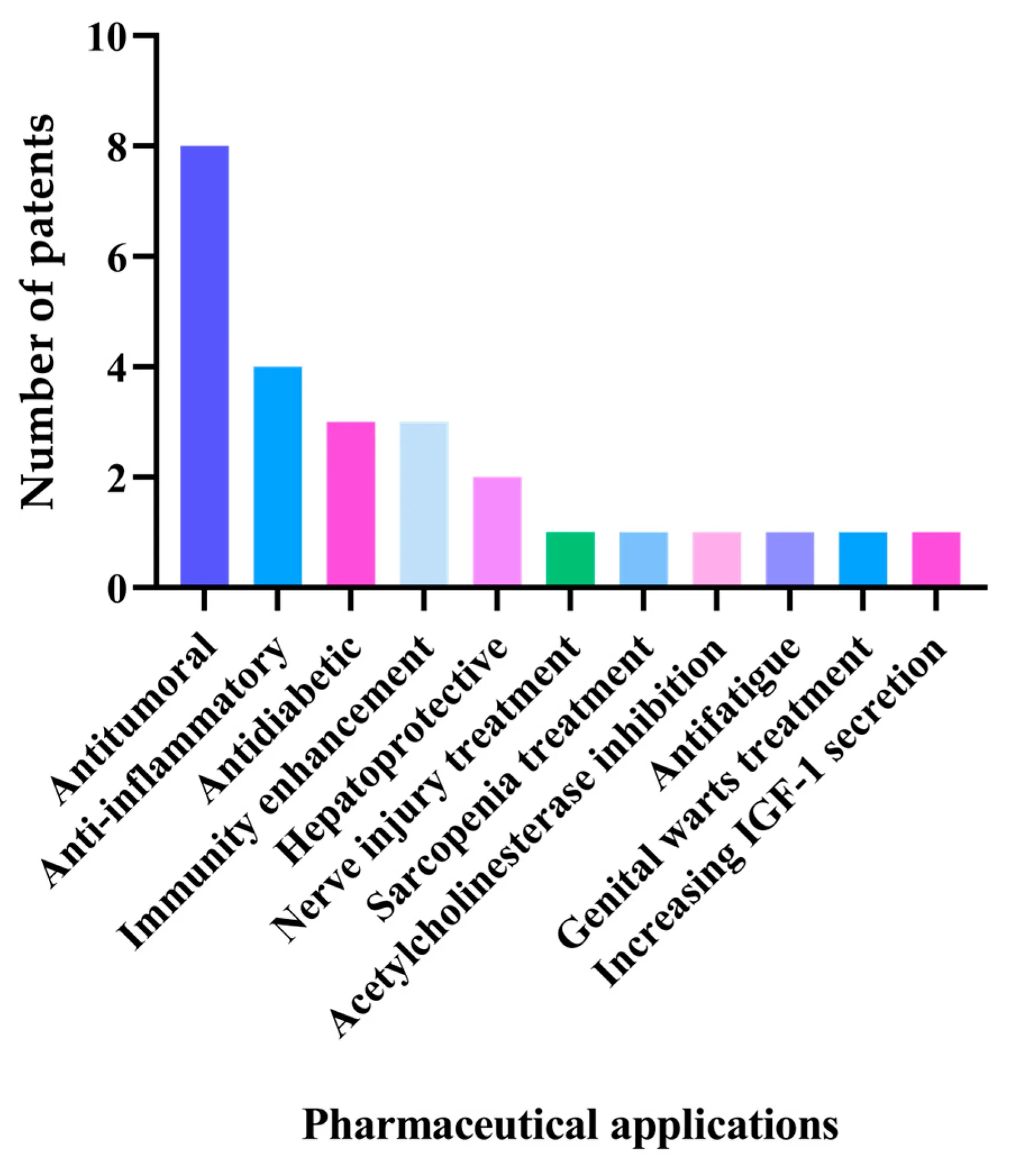
Overview of the pharmacological claims identified in the patents studied.

In this work, 26 patents described pharmacological activities for their inventions. In the next section, the main pharmacological activities claimed by the inventors will be described. The main pharmacological activities reported in the documents were antitumor, anti-inflammatory, and antidiabetic, as shown in Figure 11. In addition, details on the patents described in this section are presented in Table 1. The discussion in Section 3.4 and Section 3.5 also considers the type of evidence presented by the patents and the compositions of their inventions (Table S5).

#### 3.4.1. Antitumoral

Cancer remains a major global health challenge, and natural products have gained attention as sources of bioactive compounds for anticancer applications [45]. In this context, the biodiversity of Pantanal plant species is a valuable resource.

In this work, 8 patents described the use of *A. squamosa* for antitumoral activity. The patents CN109793732, CN106543159, CN106543117, and CN106749124 described the extraction of acetogenic lactones for this use [46,47,48,49]. Annonaceous acetogenins (ACGs) are phytochemicals with a broad range of biological activities, mostly found in plants of the Annonaceae family [50]. These compounds are effective against breast, lung, and liver cancers, as well as other cancers. The patent CN109793732 reported that the compounds Bullatacin (IC_50_ 0.1302 nM), Squamocin (IC_50_ 0.3712 nM), and Isoannonareticin (IC_50_ 0.3703 nM) exhibited cytotoxicity against choriocarcinoma cells (JEG-3) [46].

The patent CN106543159 reported the activity of extracted lactones against MCF-7/ADR (breast cancer), SMMC-7721/T (liver cancer), and A549/T (lung cancer) cell lines [47]. According to the patent data, 60.9% inhibition of liver cancer (HEPG2 cells), comparable to paclitaxel, and a 39% reduction in S180 cells at higher doses. Similarly, CN106543117 tested meta-bis(tetrahydrofuran)-type annona lactones against the same cell lines, yielding 58.7% inhibition of liver cancer and 57.8% in sarcoma [48]. Finally, patent CN106749124 reports *O*-bis(tetrahydrofuran)-type annona lactone compounds that were tested on the same cell lines, with the patent reporting 64.5% inhibition of HEPG2 cells and 62.5% inhibition of sarcoma [49].

The cytotoxicity of these ACGs and their antitumor effects may be linked to their strong inhibitory activity on mitochondrial electron transport, specifically targeting complex I. The antitumor mechanisms of action of acetogenins also include downregulating epidermal growth factor expression across cancer types, modulating metabolism and the cell cycle, inhibiting signaling pathways, the sodium-potassium pump, and the endoplasmic reticulum, while increasing cytosolic calcium and inducing genotoxicity [51].

Furthermore, the patent CN113801000 describes the extraction and use of a diterpenoid in liver cancer cells, which demonstrated an IC_50_ of 382.7 µmol/L [52]. Another enantio-kaurane-type diterpenoid described in the patent CN114671759 was shown to inhibit liver cancer cells in vitro (SMMC-7721) with an IC_50_ of 15.75 μmol/L [53]. These results corroborate the recent scientific literature, which reports cytotoxic activity of the diterpenoids present in *A. squamosa* and other Annonaceae plants [54].

The patent CN106309516 described the use of an essential oil from *A. squamosa* for the treatment of liver cancer [55]. The inventors described the oil as rich in spathulenol, palmitic acid, trimethylbicycloheptane acetate, sesquiterpenes, and steroids. The inventors performed an in vitro assay demonstrating that the essential oil blocked cell division in the G1 phase and induced apoptosis in a dose-dependent manner. CN114558053, on the other hand, conducted an in vitro assay demonstrating toxicity against gastric cancer cells (HGC-27) at an extract concentration of 0.25 mg/mL [56].

Evidence indicates that antitumor-activity technologies are primarily associated with isolated compounds from the Annonaceae family, which account for 5 of the inventions with this claim. This indicates an early stage of development for these inventions and highlights an obstacle encountered in the progress of this type of composition. Although the regulation of anticancer drugs is onerous, bureaucratic, and lengthy, it is a consequence of the need to ensure safety and efficacy. In addition, only 1 patent reported the use of a pharmaceutical dosage form, specifically a nanoparticle, which is a delivery system that enables a reduction in the necessary dose, hence decreasing side effects related to the cytotoxicity of antitumoral compounds [45].

#### 3.4.2. Anti-Inflammatory

Inflammation is the body’s response to injury or infection and is also a key factor in several diseases. Conventional anti-inflammatory agents, such as corticosteroids and nonsteroidal anti-inflammatory drugs (NSAIDs), are commonly used to manage inflammation [57]. However, these medications can cause side effects like gastrointestinal toxicity, cardiovascular risks, renal injuries, and more. Given this situation, natural products are increasingly being researched as potential sources of new bioactive compounds with anti-inflammatory properties and fewer side effects.

In this scenario, 2 plant species were reported in patents for their anti-inflammatory activities: *A. occidentale* and *C. procera*. The document EP3843762 reported that the extract from the *A. occidentale* cashew nut was able to inhibit (COX-1) and (COX-2), as well as (5-LOX) [58]. It could reduce the extracellular release of HMGB1 on murine macrophages. The invention CN112057387 focused on oral wound treatment and demonstrated efficacy in over 80% of volunteers who used the *A. occidentale* cashew peduncle extract [59].

The anti-inflammatory potential of *A. occidentale* is associated with its rich content of phenolic acids, tannins, and flavonoids [60]. The presence of bioactive compounds in this species can promote multiple mechanisms of action, including inhibition of phospholipase A and COX-2, and a decrease in inflammatory cytokines such as IL-6 and TNF-α [61]. In addition, the plant’s antioxidant activity is essential in reducing inflammation, as anacardic acids from cashew nuts reduce oxidative stress by decreasing malondialdehyde (MDA) levels and increasing reduced glutathione (GSH) and catalase (CAT) activity. The increase in antioxidant levels inhibits the inflammatory response to ROS [62].

The patent US9533019 evaluated the anti-inflammatory potential of *C. procera* in a model of ulcerative colitis; the inventors demonstrated that the plant’s aerial part extract reduced inflammation [63]. Another invention, the patent US11400126, described the use of the same plant in combination with Dead Sea extract [64]. This composition demonstrated the plant extract’s anti-inflammatory and anti-irritative potential.

The literature describes that the anti-inflammatory potential of this plant’s aerial parts stems from a latex [65]. The extract of *C. procera* reduces cytokines such as TNF-α, IL-1β, and PGE_2_, inhibits myeloperoxidase activity, and acts as an antioxidant, regulating nitric oxide levels [66].

In terms of stage of development, both inventions describe the use of simple compositions; the extracts described in the patents are generally simple and do not present any innovation in formulation. This indicates that most inventions are in the early stages of development, even though patents have been granted, suggesting that the documents presented are at the proof-of-concept stage.

**Table 1:** Patents relating to pharmacological activities. The full list of descriptions for the IPC codes is available in List S1 in the Supplementary Materials.

Title	Publication Number	Year of Publication	Grant Year	IPC Codes	Plant	Type of Composition	Activity	Ref.
Compositions comprising dead sea extract and an extract of apple of sodom and uses thereof	US11400126 (B2)	2018	2022	A61K35/08; A61K36/24; A61K8/96; A61K8/9789; A61P17/02; A61Q19/02; A61Q19/08	*Calotropis procera* (Aiton) W.T.Aiton	Extract	Anti-inflammatory	[64]
*Calotropis procera* extracts as anti-ulcerative colitis agents	US9533019 (B1)	2017	2017	A61K36/185; A61K36/27	*Calotropis procera* (Aiton) W.T.Aiton	Extract	Anti-inflammatory	[63]
Anti-inflammatory botanical extract	EP3843762 (B1)	2021	2022	A23L33/105; A61K36/22; A61P29/02	*Anacardium occidentale* L.	Extract	Anti-inflammatory	[58]
Gargle for relieving oral wound pain and preparation method thereof	CN112057387 (B)	2020	2022	A61K8/65; A61K8/81; A61K8/9789; A61P1/02; A61P31/02; A61P31/04; A61Q11/00; A61Q17/00	*Anacardium occidentale* L.	Extract	Anti-inflammatory	[59]
A pharmaceutical formulation for treating diabetes mellitus and its associated complications	IN5274/CHE/2013	2016	2021	A61K9/00	*Annona squamosa* L.	Extract	Antidiabetic	[67]
Pharmaceutical composition and health functional food comprising graviola leaf extract	KR102559349 (B1)	2022	2023	A23L33/105; A61K36/18; A61P21/00; A61P3/10	*Annona muricata L.*	Extract	Antidiabetic	[68]
Black sesame-sweetsop health-preserving wine and preparation method thereof	CN104293562 (B)	2015	2016	A61K35/28; A61K35/32; A61K35/34; A61K35/60; A61K36/899; A61P1/14; C12G3/02; C12R1/865	*Annona squamosa* L.	Other	Antidiabetic	[69]
*Annona squamosa* seed extract for treating choriocarcinoma, preparation method therefor and application thereof	CN109793732 (B)	2019	2021	A61K31/365; A61K36/185; A61P35/00; C07D307/58	*Annona squamosa* L.	Pharmaceutical composition	Antitumoral	[46]
Compound with anti-tumor activity as well as preparation method and application thereof	CN113801000 (B)	2021	2023	A61K31/047; A61P35/00; C07C29/76; C07C29/86; C07C35/44	*Annona squamosa* L.	Isolated compound	Antitumoral	[52]
Enantio-kaurane diterpenoid compound with anti-tumor activity as well as preparation method and application of enantio-kaurane diterpenoid compound	CN114671759 (B)	2022	2024	A61K31/22; A61P35/00; C07C67/56; C07C69/16	*Annona squamosa* L.	Isolated compound	Antitumoral	[53]
Epoxy type annonaceous acetogenin compound with antitumor activity and preparation method and application thereof	CN106543159 (B)	2017	2019	A61K31/365; A61P35/00; C07D407/14	*Annona squamosa* L.	Isolated compound	Antitumoral	[47]
*M*-bistetrahydrofuran annonaceous acetogenins compound with anti-tumor activity and preparation method and application thereof	CN106543117 (B)	2017	2019	A61K31/365; A61P35/00; C07D307/58	*Annona squamosa* L.	Isolated compound	Antitumoral	[48]
*O*-bis-tetrahydrofuran type annonaceous acetogenins compound having antitumor activity, preparation method, and application thereof	CN106749124 (B)	2017	2019	A61K31/365; A61P35/00; C07D307/58	*Annona squamosa* L.	Isolated compound	Antitumoral	[49]
Sweetsop peel volatile oil extract with anti-tumor activity as well as preparation method and application thereof	CN106309516 (B)	2017	2020	A61K36/185; A61P35/00; C11B1/10	*Annona squamosa* L.	Essential oil	Antitumoral	[55]
Waste fruit peel extract composition with effect of treating gastric cancer	CN114558053 (B)	2022	2022	A61K36/61; A61P35/00; A61P35/04	*Annona squamosa* L.	Extract	Antitumoral	[56]
Composition for enhancing immunity as well as preparation technology and application thereof	CN113244372 (B)	2021	2023	A61K31/519; A61K36/815; A61K38/16; A61P37/04	*Annona squamosa* L.	Other	Immunity enhancement	[70]
Method for manufacturing graviola extract and pharmaceutical composition for immune enhancement	KR101951854 (B1)	2017	2019	A61K36/185	*Annona muricata* L.	Extract	Immunity enhancement	[71]
Composition containing *Cistanche deserticola* extract as well as preparation method and application of composition	CN118593682 (B)	2024	2024	A23L33/105; A23L33/185; A61K36/64; A61K38/02; A61P37/04	*Anacardium occidentale* L.	Other	Immunity enhancement	[72]
Extract composition with effects of protecting liver and lowering transaminase	CN114504615 (B)	2022	2022	A61K36/77; A61P1/16	*Annona squamosa* L.	Extract	Hepatoprotective	[73]
Composition for treating hepatitis containing an extract of *Cordia lutea* flowers, *Annona muricata* leaves, and *Curcuma longa* roots	US9775871 (B2)	2015	2015	A61K36/30; A61K36/57; A61K36/9066; A61P1/16; A61P31/12; A61P39/06	*Annona muricata* L.	Extract	Hepatoprotective	[74]
Pharmaceutical composition for treating or preventing nerve damage	CN110075274 (B)	2019	2020	A61K31/7024; A61K36/886; A61K36/896; A61K38/18; A61P25/00	*Annona squamosa* L.	Extract	Nerve injury	[75]
Muscle activator	JP7209207 (B2)	2019	2023	A23L33/105; A61K36/428; A61P17/00; A61P21/00; A61P21/06; A61P3/00	*Annona muricata* L.	Extract	Sarcopenia	[76]
Amino derivatives of LCC as acetylcholinesterase inhibitors, processes for their production, pharmaceutical compositions containing them and applications	BR102013012924 (B1)	2015	2023	A61K31/137; A61P25/00; A61P25/16; C07D207/08; C07D211/32; C07D211/40; C07D241/04; C07D269/00; C07D283/00	*Anacardium occidentale* L.	Isolated compound	Acetyl cholinesteraserase inhibitors	[77]
Health maintaining and kidney tonifying Chinese chestnut baijiu and preparation method thereof	CN105733899 (B)	2016	2018	A61K35/644; A61K36/8969; A61P13/12; C12G3/04	*Anacardium occidentale* L.	Other	Antifatigue	[78]
A herbal cream preparation that cauterizes and removes condyloma acuminata on genital warts and the likes	PH1/2013/000108 (B1)	2015	2015	A61K36/22; A61K36/31; A61K36/752; A61K9/06; A61P17/00; A61P17/12; A61P31/00; A61P31/12; A61P31/20	*Anacardium occidentale* L.	Other	Genital warts	[79]
Plant extract pharmaceutical composition for improving secretion of endogenous insulin-like growth factor 1	CN117257891 (B)	2023	2024	A23L19/00; A23L25/00; A23L31/00; A23L33/10; A23L33/105; A23L33/125; A61K31/7016; A61K36/899; A61P1/00; A61P19/10; A61P25/16; A61P25/28; A61P29/00; A61P3/10; A61P35/00; A61P39/00; A61P9/10	*Anacardium occidentale* L.	Extract	Increase secretion of IGF-1	[80]

Ref: Own authorship.

#### 3.4.3. Antidiabetic

Diabetes mellitus is a chronic metabolic disorder characterized by impaired insulin secretion, impaired insulin action, or both [81]. Natural products have been reported to treat diabetes through multiple mechanisms [81].

From this point of view, the document IN5274/CHE/2013 related the use of a composition of multiple herbs, including *A. squamosa*, which inhibited the activity of α-amylase and α-glycosidase, showed an effect on peroxisome proliferator-activated receptor (PPAR) genes, and had antioxidant activity by increasing the levels of antioxidants such as superoxide dismutase (SOD), catalase (CAT), glutathione peroxidase (GPx), and glutathione (GSH) [67]. The patent CN104293562 also demonstrated the plant’s benefits in reducing glucose levels and its antioxidant potential [69].

The patent KR102559349 described the use of *A. muricata* extract, which inhibited the formation of advanced glycation end products (AGEs) and MGO, exhibited anti-inflammatory effects by reducing NO levels, promoted greater glucose tolerance, and reduced diabetes-induced hepatic damage [68].

The antidiabetic effect of *A. muricata* can be attributed to compounds such as flavonoids, which contribute to the plant’s antioxidant activity. The patents described for antidiabetic purposes primarily claim the use of extracts; these compositions, although effective, are not ready-to-use, compared with dosage forms already available for the treatment of diabetes. In contrast, the use of extracts is better supported, as such compositions can target multiple sites due to the presence of multiple bioactive compounds in a single formulation [82].

#### 3.4.4. Hepatoprotective

The liver is a vital organ that performs various functions, including metabolism, secretion, storage, and detoxification of endogenous and exogenous compounds [83]. Hepatic diseases pose a significant health problem, associated with 2 million deaths worldwide and 4% of global mortality [83]. Natural products have emerged as a approach to hepatoprotection; their antioxidant and anti-inflammatory properties help prevent liver cell damage [84].

From this perspective, the patent CN114504615 used an *A. squamosa* extract to promote liver health; the inventors reported reductions in serum ALT and AST levels, as well as decreases in TNF-α and IL-6 after treatment with the *A. squamosa* extract in combination with passion fruit and lichen extracts [73]. Additionally, the document US9775871 described the use of extracts of *Cordia lutea*, *A. muricata*, and *Curcuma longa* [74]. The inventors demonstrated reduced free radical formation and restored GSH levels; clinical trials also demonstrated reduced hepatitis symptoms, increased hepatic enzymes (TGO, TGP, and GGT), and hepatocyte proliferation.

The hepatoprotective potential of both *A. squamosa* and *A. muricata* may be attributed to their antioxidant constituents, including phenolic compounds and flavonoids [85]. The antioxidant potential of these plants is well documented in the literature; this activity protects cell membranes by neutralizing free radicals, thereby protecting liver cells against oxidative stress [84,86].

The documents describing liver-protective activities showed the great potential of Annonaceae plants for this activity. However, the inventions are in the early stages of development of the claimed technologies. Notably, both described the use of extracts. Furthermore, there was no evidence of innovative extraction methods, and the inventors’ assays indicate that the inventions are at the proof-of-concept stage.

#### 3.4.5. Immunity Enhancement

The immune system is a complex network of cells, tissues, and organs that works to prevent infections and diseases [87]. Factors such as diet, physical activity, sleep, and unhealthy lifestyle habits can impair immune function.

In this context, the patent CN113244372 discloses a composition for enhancing immunity using *A. squamosa* fruit extract, which promotes lymphocyte proliferation [70]. The document KR101951854 describes a composition that uses the extract of *A. muricata* leaves for the same purpose; it demonstrates immune activation of macrophages, increased transcription of cytokines TNF-α, IL-1β, and iNOS, and macrophage activation mediated by MAPK signaling [71]. Finally, the inventors of patent CN118593682 described immune enhancement via antibody production and the proliferation of defense cells using a composition comprising *Cistanche deserticola*, rose extract, ginseng extract, and a cashew protein peptide (*A. occidentale*) [72].

Natural products are known for their immune-boosting properties due to their high levels of polyphenols, terpenoids, β-glucans, vitamins, and other bioactive compounds. These substances enhance the immune response by directly increasing the cytotoxic activity of macrophages, natural killer (NK) cells, and neutrophils [87]. Plants such as *A. squamosa* have been reported to enhance immunity, particularly through polyphenols that can boost immune function and protect cells against ROS-mediated damage [88]. In addition, cashew protein peptides have been reported to possess immunomodulatory and antioxidant properties [89].

The use of complex extracts is evidenced in this activity. These extracts support this approach; their synergistic, multi-target action makes this type of composition more advantageous than isolated compounds [90]. The diversity of compounds in these compositions triggers different effects and complements the extracts’ actions on the immune system.

The described patents demonstrate strong potential for developing nutraceuticals and food supplements that target the immune system. However, these inventions are at an early stage of development and technological maturity and require further clinical trials and regulatory approval.

#### 3.4.6. Others

Other pharmacological activities were also reported in the patents analyzed. These include treating nerve damage (CN110075274; *A. squamosa*) and sarcopenia (JP7209207; *A. muricata*), both of which are associated with increased muscle tone after use [75,76]. Despite the findings in these documents, the literature does not describe the use of either plant for the activities claimed. Nerve injury disrupts communication between the brain and peripheral tissues and organs [91]. The treatment of this injury primarily involves medications, particularly neurotrophic agents, antioxidants, and anti-inflammatory agents [91]. Considering this perspective, Annonaceae plants may influence the treatment of nerve damage.

The patent BR102013012924 isolated aminic derivatives from cashew nuts that act as acetylcholinesterase inhibitors with low IC_50_ values [77]. Another patent, CN105733899, described a composition that acted on fatigue, enhanced libido and immunity, and promoted energy balance [78]. The invention PH1/2013/000108 describes the use of cashew pericarp juice, in combination with other ingredients (*Carica papaya*, lemon juice, apple cider vinegar, sodium hydroxide, and talcum powder), for the treatment of condyloma acuminatum [79]. Finally, the document CN117257891 reported the use of cashew nut extract to increase insulin-like growth factor (IGF-1) secretion, indicating a progressive increase in this protein [80].

*A. occidentale* is a plant with numerous pharmacological activities, including neuroprotective potential. Phenolic compounds from cashew nut shell liquid (CNSL) have been studied for their potential to inhibit acetylcholinesterase (AChE), and the cardanol derivatives present in CNSL also exhibit anti-amyloid and antioxidant capacities [92]. This inhibition is one of the mechanisms of action of the treatment of neurodegenerative diseases like Alzheimer’s disease. Conversely, the literature reports no increase in IGF-1 induced by this plant, underscoring the need for further studies to corroborate the activities reported in the patents.

The patents described in this section, mostly extracts, relate to the use of Pantanal plants in the treatment of nerve injury, sarcopenia, and neurodegenerative diseases; as antifatigue agents; for the treatment of genital warts; and for increasing IGF-1 secretion. Although the patents provide assays to support their claims, some activities are not described in the literature, indicating an early level of maturity for these inventions; thus, further studies to provide clinical confirmation of these activities are warranted. Furthermore, beverages and creams were developed in 2 inventions to deliver bioactive compounds, thereby encouraging technology transfer.

### 3.5. Cosmetic Activities

**Figure 12:**
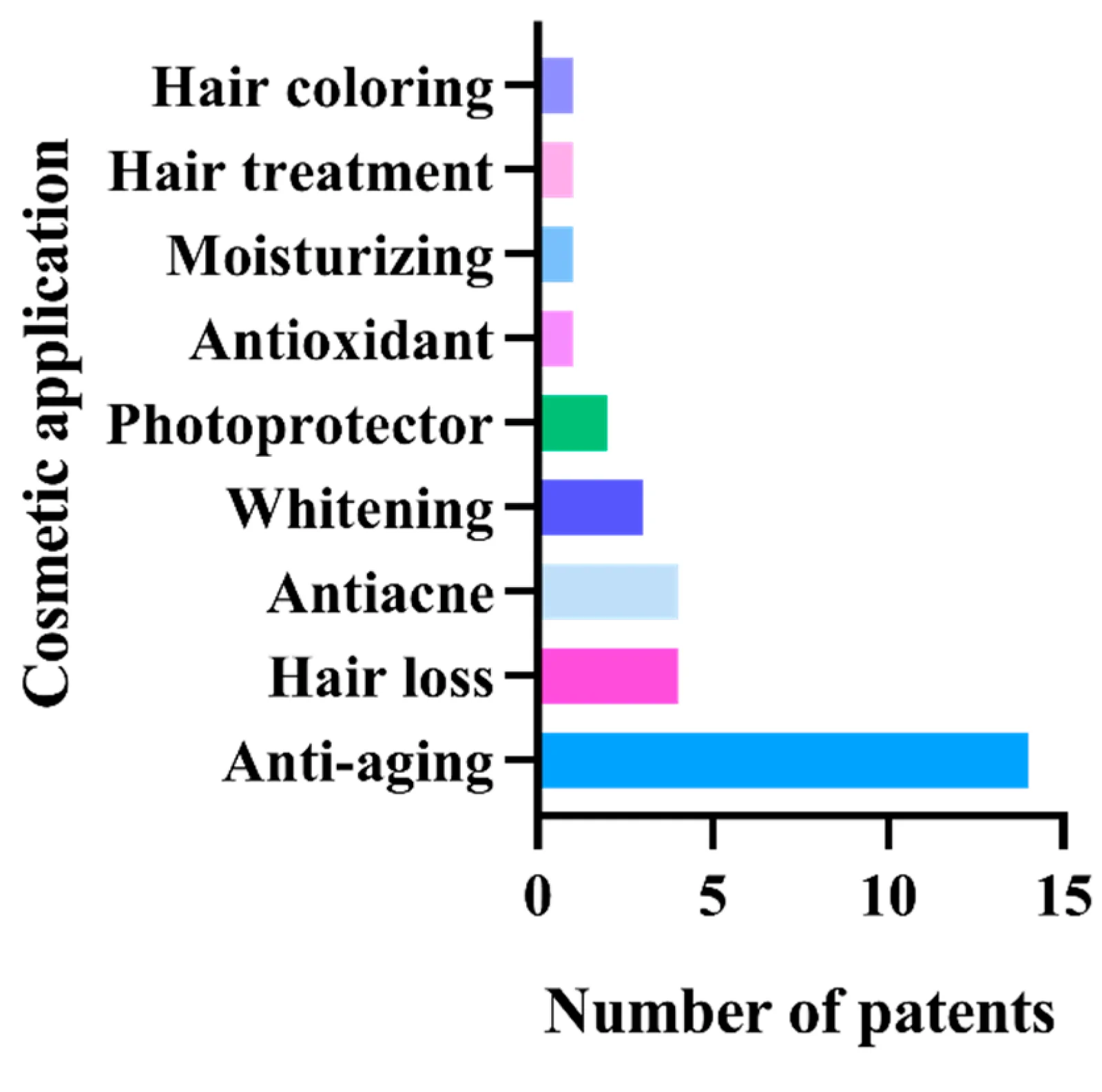
Main cosmetic claims from the patents.

In this work, cosmetic activities were also assessed. The main cosmetic claims included anti-aging, anti-acne, antioxidant, whitening, photoprotective, hair-loss aid, and others, as shown in Figure 12. In the next sections, the highlights of the inventions will be described, and a summary of the inventions is provided in Table 2.

#### 3.5.1. Anti-Aging

Aging is a natural degenerative process that involves a range of molecular mechanisms and cellular systems [93]. This process is characterized by tissue degeneration, telomere shortening, and other health and cosmetic implications [94]. As the use of natural products in cosmetic preparations increases, the compounds present in these products may offer alternatives for cosmetic antiaging treatments.

In this view, 14 patents describe cosmetic compositions with antiaging properties. Among these, the document TWI774339 describes the use of *A. montana* for its anti-inflammatory, antioxidant, whitening, repairing, and anti-wrinkle properties [95]. The patent, although for cosmetic purposes, supported the previously mentioned antioxidant and anti-inflammatory properties of this species. The patents TWI814026 and KR101619709 also highlight the antioxidant effect of *A. squamosa* extract [96,97]. Together, they also evidence this plant’s inhibition of hyaluronidases and metalloproteinases, inhibition of melanin synthesis, enhancement of procollagen production, inhibition of sebum production, and moisturizing effect. Similarly, the invention CN113041200 showed a composition with antioxidant, moisturizing, and whitening effects [98]. A test conducted with volunteers using this composition demonstrated reduced wrinkles and fine lines, improved skin texture, and fewer skin spots. Similarly, the document CN111956536 described a fermented extract of *A. squamosa* that reduces wrinkles, reduces collagen loss, promotes the expression of anti-aging genes, moisturizes, and exhibits anti-inflammatory properties [99].

Regarding the benefits of *A. muricata*, the patent KR102436816 reports the antioxidant potential and the inhibition of melanin synthesis, tyrosinase, and elastase [100]. KR101648148, on the other hand, used an extract that promotes fibroblast proliferation and increases the expression of genes related to collagen production [101]. The patents KR101999885, KR101868507, and KR101774408 reported the anti-wrinkle properties of this plant using plant extracts in volunteers [102,103,104]. Such an effect was attributed to different mechanisms.

Building on the advantages of *A. occidentale*, the invention EP3843557 relates to effects on tyrosinase, which causes whitening; it also activates antioxidant genes, reduces markers of fibrosis and aging, and stimulates skin repair and healing [105]. In a more complex composition, the inventors of KR102726643 describe using a nanoemulsion that is then further transformed into creams or serums [106]. The inventors demonstrated that the composition reduced wrinkles in volunteers. Additionally, a patent describing the use of an *A. occidentale* extract (KR102693360) reported effects on procollagen synthesis, inhibition of inflammatory cytokines, inhibition of hyaluronidase, and increased expression of Hyaluronan Synthase-3 [107]. In a similar context, US10967022 reported that a cashew nut extract inhibited tyrosinase and induced changes in genes related to antioxidant protection, cell renewal and regeneration, wound healing, anti-aging, and immune/inflammatory responses [108].

Skin aging is inevitable; however, by modulating factors such as oxidative stress and chronic inflammation, and by stimulating protein degradation or promoting protein synthesis, including collagen, this process can be slowed [109,110]. Both Annonaceae plants and *A. occidentale* contain compounds with antioxidant and anti-inflammatory activities that may help delay skin aging. As noted above, plants in the genus *Annona* are rich in antioxidant bioactive compounds, including polyphenols, proanthocyanins, and flavonoids [111]. *A. occidentale* also contains compounds with antioxidant properties, such as flavonoids, alkaloids, terpenoids, and phenolic compounds [112]. These compounds may also contribute to its anti-inflammatory properties by inhibiting the production of inflammatory cytokines [60,61]. They may also act by promoting collagen and elastin synthesis [113,114]. Similar to *A. occidentale*, *A. squamosa* has been shown to promote collagen synthesis, supporting wound healing and contributing to its anti-aging properties. Despite the anti-aging potential reported in patents, most studies use simple extracts rather than more complex formulations. This indicates that the development of these cosmetics remains in its initial phase. Nevertheless, the use of nanoemulsions can modify release, increase the active ingredient’s efficacy by increasing the contact surface area in topical formulations, improve stability, and enhance the solubility of various ingredients [115]. Therefore, patents involving this type of delivery system are promising, as they demonstrate greater technological maturity and applicability than extracts alone. However, formulation complexity should not be interpreted as evidence of greater technological maturity, which depends on the level of experimental validation reported for each invention.

#### 3.5.2. Hair Loss

Hair loss is a naturally occurring, age-related physiologic process; however, it is also the main characteristic of a disorder called alopecia [116]. This condition is classified in scarring alopecia, non-scarring alopecia, and structural hair disorders [117]. The most prevalent type of alopecia is non-scarring alopecia, and most cases are androgenetic alopecia or alopecia areata [118].

Natural products are known for their diverse properties. In hair loss treatment, plant extracts can act on multiple pathways through bioactive compounds such as polyphenols, terpenes, and fatty acids [117]. These compounds can stimulate the anagen phase of hair growth and reduce inflammation and oxidative stress, thereby treating the symptoms of alopecia.

Accordingly, this study revealed that patents described the use of *A. muricata* to treat hair loss and alopecia; these inventions involve extracts and their incorporation into shampoos. The document KR102143154 describes a shampoo containing extracts of *A. muricata* and ginseng, with volunteer trials showing reduced hair loss [119]. Similarly, KR102585336 describes a polyherbal shampoo that reduces alopecia in volunteers [120]. Furthermore, KR101540333 reports a shampoo containing bongsam, graviola, and moringa that reduced hair loss in volunteers [121]. Finally, the document KR102080412 describes a shampoo with antifungal properties, strong cleaning efficacy, and reduced hair loss [122].

Despite substantial evidence supporting the benefits of *A. muricata* for hair loss and alopecia, the literature lacks descriptions of this activity. However, moringa and ginseng have been reported to be used in the treatment of alopecia [123,124]. Given the findings, further studies are needed to substantiate this activity and identify the compounds responsible.

Interest in *A. muricata* is evidently growing for use in hair products. This represents a new application for plants from the Pantanal, which are poorly described in the literature to date. The findings demonstrate the efficacy of the claimed shampoos and extracts; however, these studies are based on empirical observations and do not elucidate the molecular mechanisms underlying this activity. These data suggest an early stage of development for these technologies and indicate the need for biochemical validation to identify the primary compounds responsible for this effect. Once the compounds are identified, they can be isolated and subjected to clinical assays to elucidate the mechanisms underlying this activity. This path would enable the development of appropriate and reproducible natural-product cosmetic ingredients derived from this plant. Finally, the compositions described in this section align with the trend towards natural and sustainable cosmetics and the market demand for plant-based hair products.

#### 3.5.3. Whitening

The whitening effect is a popular trend in cosmetics and traditional dermatology treatment. The market for skin whitening products is estimated at $8.8 billion annually and is expected to grow to $15.7 billion by 2030 [125]. Melanin is an important substance that protects the skin against UV damage; however, overproduction of melanin is an aesthetic and dermatological problem. At the core of these issues, tyrosinase is primarily responsible for melanin synthesis [126]. Skin lightening can be achieved through chemical or physical methods that have depigmenting potential, thereby lightening or evening the skin tone [126].

The main chemical whitening mechanism involves tyrosinase inhibition. The majority of market products that claim to whiten skin are tyrosinase inhibitors; they reduce skin pigmentation by inhibiting melanin production [127]. Kojic acid and hydroquinone are the most widely used tyrosinase inhibitors currently on the market, often used at concentrations that categorize them as medicines rather than cosmetics in several countries. These molecules are phenolic compounds, characterized by a benzene ring, that interact with tyrosinase, inhibiting its activity [128].

The use of natural products for this cosmetic purpose has increased over time. The rapid dissemination of information, heightened awareness of the risks associated with synthetic chemicals in cosmetics, and perceived benefits of natural products drive this trend [129].

In this context, the patent TWI817363 describes the use of a fermented extract of *A. muricata* to develop a whitening composition [130]. The inventors demonstrated that the extract exhibits antioxidant properties and a whitening effect superior to that of kojic acid. Another invention (KR102676679) describing the use of *A. muricata* in whitening compositions reports the use of an extract obtained from an induced callus of the plant [131]. This extract promoted keratinocyte and fibroblast proliferation, exhibited excellent antioxidant activity, and inhibited melanin synthesis by inhibiting tyrosinase.

The findings in the patent documents are corroborated by the scientific literature, which demonstrates *A. muricata*’s potential for skin whitening. Joo and colleagues investigated the potential of *A. muricata* extract to inhibit melanogenesis and showed that it inhibited tyrosinase and MITF in a dose-dependent response [132]. The compounds responsible for this activity are yet to be elucidated.

Similarly, the invention CN118512378 describes the use of *A. occidentale* for skin whitening [133]. The inventors used a fermented cashew nut extract in combination with other extracts; this formulation demonstrated strong tyrosinase inhibition, resulting in a whitening effect. The presence of anacardic acid and its derivatives is primarily responsible for this whitening effect. They act by inhibiting tyrosinase, suggesting potential for use in cosmetics [134].

The patents describing skin-whitening methods revealed that species found in the Pantanal are being incorporated into cosmetic formulations through innovative biotechnological approaches, such as fermentation and callus induction. These strategies enhance the stability and bioavailability of bioactive compounds, thereby reducing reliance on synthetic depigmenting agents. A strong inhibition of tyrosinase activity in *A. muricata* and *A. occidentale* reflects their profiles rich in phenolic compounds and anacardic acid, positioning these plants as viable sources for skin-whitening cosmetics. However, most of the inventions described remain at the pre-commercial stage, underscoring the need for compound isolation or extract standardization, safety assessment, and formulation development and scale-up. Strengthening this type of research can solidify the Pantanal’s biodiversity as a sustainable source of innovative cosmetic technologies.

**Table 2:** Patents relating to cosmetic activities. The full list of descriptions for the IPC codes is provided in List S1 in the Supplementary Materials.

Title	Publication Number	Year of Publication	Grant Year	IPC Codes	Plant	Type of Composition	Activity	Ref.
*Annona montana* Macf leaves extract for anti-oxidant, anti-inflammation, whitening, repairing and anti-wrinkle	TWI774339 (B)	2021	2022	A23L33/105; A61K36/185; A61K8/9789; A61P17/00; A61P29/00; A61Q19/00	*Annona montana* Macfad.	Extract	Anti-aging	[95]
Anti-aging composition for skin external application comprising *annona squamosa* placenta culture extracts	KR101619709 (B1)	2015	2016	A61K36/185; A61K8/97; A61Q19/02; A61Q19/08	*Annona squamosa* L.	Extract	Anti-aging	[97]
Cosmetic containing custard apple multiple fermentation liquor and preparation method thereof	CN113041200 (B)	2021	2021	A61K8/9789; A61Q19/00; A61Q19/02; A61Q19/08; C12P1/02; C12P1/04; C12P39/00; C12R1/01; C12R1/125; C12R1/225; C12R1/865	*Annona squamosa* L.	Fermented extract	Anti-aging	[98]
Use of *Annona squamosa* L. Peel extract in preparation of composition for inhibiting matrix metalloproteinase, hyaluronidase, lipoxygenase activity and nitric oxide generation	TWI814026 (B)	2021	2023	A61K36/185; A61K8/9789; A61P43/00; A61Q19/08	*Annona squamosa* L.	Extract	Anti-aging	[96]
Application of *Annona squamosa* leavening in preparation of composition	CN111956536 (B)	2020	2022	A23L19/00; A23L29/00; A61K36/185; A61K8/9789; A61P17/00; A61P29/00; A61P37/04; A61P39/06; A61Q19/00; A61Q19/02; A61Q19/08	*Annona muricata* L.	Extract	Anti-aging	[99]
Cosmetic composition	KR102436816 (B1)	2022	2022	A61K8/9783; A61Q19/00; A61Q19/02; A61Q19/08	*Annona muricata* L.	Extract	Anti-aging	[100]
Cosmetic composition containing fermented graviola for improving anti-wrinkle and method of preparing the same	KR101648148 (B1)	2016	2016	A61K8/97; A61K8/99; A61Q19/08	*Annona muricata* L.	Extract	Anti-aging	[101]
Cosmetic composition containing the extraction mixture	KR101999885 (B1)	2018	2019	A61K8/60; A61K8/97; A61Q19/00	*Annona muricata* L.	Extract	Anti-aging	[102]
Cosmetic Composition for Anti-oxidation Anti-inflammatory and Anti-wrinkling Comprising Enzyme-treated Extracts of *Annona muricata* and Method for Manufacturing the Same	KR101868507 (B1)	2018	2018	A61K8/9789; A61Q19/00; A61Q19/08	*Annona muricata* L.	Extract	Anti-aging	[103]
Cosmetic Composition for Silver Generation for Improving Skin Winkles and maskpack Sheet using the same	KR101774408 (B1)	2017	2017	A61K8/02; A61K8/97; A61Q19/08	*Annona muricata* L.	Extract	Anti-aging	[104]
Botanical Extract for Skin Care	US10967022 (B2)	2020	2021	A61K36/22; A61K8/9789; A61P17/18; A61P29/00	*Anacardium occidentale* L.	Extract	Anti-aging	[108]
Cosmetic composition including as effective ingredient an nanoemulsion of cashew seed oil	KR102726643 (B1)	2023	2024	A61K8/06; A61K8/368; A61K8/92; A61K8/9789; A61Q19/08	*Anacardium occidentale* L.	Cosmetic composition	Anti-aging	[106]
Cosmetic composition for improving skin wrinkle anti-inflammation and moisturizing containing the complex extracts of *Morus Bombycis* Leaf, *Polygonum Fagopyrum* Seed, *Moringa Oleifera* Seed and *Anacardium Occidentale*	KR102693360 (B1)	2024	2024	A61K8/9789; A61Q19/00; A61Q19/08	*Anacardium occidentale* L.	Extract	Anti-aging	[107]
Botanical antioxidants	EP3843557 (B1)	2021	2023	A23L33/105; A61K36/22; A61P39/06	*Anacardium occidentale* L.	Extract	Anti-aging	[105]
2 manufacturing method of hair loss prevention shampoo containing graviola component extracted in two steps	KR102143154 (B1)	2020	2020	A61K8/9783; A61Q5/02; A61Q7/00	*Annona muricata* L.	Extract	Hair loss	[119]
Fermented product of medical herbs for preventing, ameliorating, or protecting against hair loss, and use thereof	KR102585336 (B1)	2023	2023	A61K8/9761; A61K8/9789; A61K8/9794; A61Q7/00	*Annona muricata* L.	Cosmetic composition	Hair loss	[120]
Method for producing natural extract with increased alopecia prevention effect and shampoo for preventing alopecia containing natural extract produced by same method	KR101540333 (B1)	2015	2015	A61K8/02; A61K8/97; A61Q5/02; A61Q7/00	*Annona muricata* L.	Cosmetic composition	Hair loss	[121]
Shampoo comprising the extract of graviola for improving condition of scalp and preventing hair loss	KR102080412 (B1)	2020	2020	A61K8/64; A61K8/92; A61K8/9783; A61Q19/00; A61Q5/02; A61Q7/00	*Annona muricata* L.	Cosmetic composition	Hair loss	[122]
Use of bioactive compounds of *Annona muricata* ferment for preparing a composition of inhibiting melanin production and enhancing anti-oxidation capacity of THP-1 cells	TWI817363 (B)	2022	2023	A23L33/105; A61K31/05; A61K31/192; A61K31/216; A61K31/7004; A61K36/18 A61K8/9789; A61P17/00; A61Q19/00	*Annona muricata* L.	Extract	Whitening	[130]
Composition comprising Graviola callus lysate extract as an active ingredient having skin whitening antioxidant skin condition improvement and hair loss inhibitory effects	KR102676679 (B1)	2024	2024	A61K36/185; A61K8/9789; A61P17/00; A61P17/02; A61P17/14; A61Q19/00; A61Q19/02; A61Q7/00	*Annona muricata* L.	Extract	Whitening	[131]
Whitening composition	CN118512378 (B)	2024	2025	A61K8/9789; A61K8/9794; A61Q19/00; A61Q19/02; A61Q19/08	*Anacardium occidentale* L.	Extract	Whitening	[133]
Cosmetic compositions containing niosomes of graviola extract	KR101844673 (B1)	2018	2018	A61K8/14; A61K8/92; A61K8/97; A61Q19/00	*Annona muricata* L.	Cosmetic composition	Antiacne	[135]
Mask cosmetic composition comprising Graviola antibiotic extract for skin anti-wrinkle and method of preparing the same	KR102475897 (B1)	2017	2022	A61K8/02; A61K8/30; A61K8/97; A61Q19/00; A61Q19/08	*Annona muricata* L.	Cosmetic composition	Antiacne	[136]
Skin vital rising cosmetic composition comprising Graviola antibiotic extract and method of preparing the same	KR102475898 (B1)	2017	2022	A61K8/02; A61K8/30; A61K8/97; A61Q19/00	*Annona muricata* L.	Extract	Antiacne	[137]
Moisturizing, anti-wrinkle, nourishing and skin-care gel and preparation method thereof	CN113368026 (B)	2021	2024	A61K8/31; A61K8/34; A61K8/37; A61K8/72; A61K8/73; A61K8/92; A61K8/9789; A61K8/9794; A61Q19/00	*Anacardium occidentale* L.	Cosmetic composition	Antiacne	[138]
Cosmetic composition for preventing and improving skin damage caused by ultraviolet rays and method for manufacturing the same	KR102679087 (B1)	2024	2024	A61K8/365; A61K8/67; A61K8/73; A61K8/9789; A61Q17/04; A61Q19/00	*Annona muricata* L.	Extract	Photoprotective	[139]
Compounds having excited state intramolecular proton transfer (esipt) character for use in treating and/or preventing sunburn and/or preventing u.v. damage	AU2020234097 (B2)	2020	2023	A61K31/351; A61P17/00; C07D251/14; C07D309/12	*Anacardium occidentale* L.	Isolated compounds	Photoprotective	[140]
Male conditioning essence and preparation method thereof	CN114515261 (B)	2022	2023	A61K8/34; A61K8/35; A61K8/49; A61K8/63; A61K8/64; A61K8/67; A61K8/73; A61K8/92; A61K8/9789; A61Q19/00	*Annona squamosa* L.	Cosmetic composition	Moisturizing	[141]
A composition comprising graviola having inhibitory effect on induction of gray hair and positive effect on induction of black hair	KR102030419 (B1)	2019	2019	A23L33/105; A61K8/9783; A61K8/9789; A61K8/9794; A61Q5/00; A61Q7/00	*Annona muricata* L.	Extract	Hair coloring	[142]
Preparation d’une levure acetobacter et sa mise en evidence	FR3051363 (B3)	2017	2018	A61K8/97; A61Q5/00	*Annona muricata* L.	Other	Hair treatment	[143]
Hair dye and hair dyeing method	JP6049830 (B2)	2015	2016	A61K8/19; A61K8/27; A61K8/34; A61K8/41; A61K8/42; A61K8/44; A61K8/46; A61K8/97; A61Q5/10	*Anacardium occidentale* L.	Cosmetic composition	Hair coloring	[144]
Natural cosmetic composition using aronia	KR101957351 (B1)	2019	2019	A61K8/06; A61K8/97; A61Q19/00; A61Q19/02	*Anacardium occidentale* L.	Extract	Antioxidant	[145]

Ref: Own Authorship.

#### 3.5.4. Anti-Acne

Acne is an inflammatory response that affects the pilosebaceous unit (PSU) [146,147]. It presents a multifactorial physiopathology that includes hyperkeratinization of the PSU duct, which can lead to comedone formation; increased sebum production; presence of inflammatory mediators around the PSU; and hyperproliferation of *Cutibacterium acnes* [148]. Several plants contain compounds that may help treat acne. In this context, the patents in this study claimed the use of different plant compositions for acne-related treatments.

The invention KR101844673 describes the antimicrobial potential of *A. muricata* extract incorporated into niosomes [135]. This formulation showed activity against *Staphylococcus aureus*, *Staphylococcus epidermidis*, *Escherichia coli*, and *C. acnes*. The formulation also exhibited antioxidant activity, inhibited the proliferation and differentiation of sebaceous cells, and reduced inflammatory cytokines. Another invention, KR102475897, describes a facial mask for skin care containing an extract of *A. muricata* that exhibited activity against *C. acnes*, enhanced the proliferation of keratinocytes and fibroblasts, reduced melanin production, inhibited the production of inflammatory cytokines, and showed antioxidant activity [136]. Finally, the patent KR102475898 reported the use of *A. muricata* extract, which also showed activity against *C. acnes* [137].

*A. muricata* is a plant with numerous biological activities; its potential to overcome inflammation makes the products with this active ingredient desirable for the treatment of acne vulgaris [149]. In addition, extracts from *A. muricata* have demonstrated strong antibacterial activity against both Gram-positive and Gram-negative bacteria [150]. However, data on *A. muricata*’s potential to inhibit *C. acnes* are limited to date.

The document CN113368026 described the use of a cashew nut extract, which showed reduced acne, improved acne scar repair, and reduced acne recurrence in volunteers [138].

For *A. occidentale*, findings report antibacterial activity against several bacterial species. The literature describes the potential of this plant to inhibit *C. acnes*, an activity largely attributed to its anacardic acids [151].

Unlike most therapeutic and cosmetic claims in this review, only 1 patent described the use of an extract for acne treatment, and the other 3 compositions relate to more complex formulations. This is the case for niosomes, facial masks, and gels claimed to have an anti-acne effect. This indicates that researchers in acne treatment are focusing on developing delivery systems to overcome the barriers posed by simple extracts when administered topically. A drawback of applying an extract to the skin is that the composition may not reach the pilosebaceous unit, the site of action. This is challenging because the stratum corneum is a strong barrier [152]. Using small vesicles, such as niosomes, reduces size, enabling delivery of active compounds to the target site [153]. In addition, when applied to other vesicles, this strategy enhances bioavailability, prolongs action, and reduces systemic exposure [152]. Finally, the pathways presented in the documents address key aspects of acne treatment: activity against *C. acnes*, anti-inflammatory mechanisms, control of sebum production, and keratinocyte activity. In this scenario, these compositions demonstrate the multi-target potential of Pantanal plants for anti-acne applications.

#### 3.5.5. Photoprotective

Solar radiation causes diverse effects on human cells, influencing individual health. Ultraviolet radiation is a major cause of skin damage; effects such as sunburn, wrinkles, reduced skin elasticity, and DNA damage result from UV exposure [152]. In this context, the use of photoprotective agents is crucial for reducing skin damage and DNA alterations that may lead to skin cancer.

The patent KR102679087 describes the use of an *A. muricata* extract for photoprotection [139]. The inventors conducted an in vitro study to determine the sun protection factor (SPF) of the composition, yielding a value of 27. Recent research findings corroborate the results in this document. This plant’s activity is associated with the presence of phenolic compounds and acetogenins; the SPF values for these compounds have been reported as 18.9 and 26.2, respectively, indicating the plant’s photoprotective potential [154].

The invention AU2020234097 describes the isolation of compounds from *A. occidentale*, including anacardic acid, cardanol, and aldehydes, for the treatment and prevention of sunburn and UV damage [140]. The inventors did not provide a study to assess the compounds’ SPF. Nevertheless, they showed that the isolated compounds exhibit strong UV absorption, suggesting potential applications in sunscreen products. The literature indicates the photoprotective potential of *A. occidentale*; however, scientific reports indicate that the plant extract’s SPF is relatively low (6.12) and is mainly attributable to flavonoids, tannins, phenols, and coumarins [155,156].

SPF is a numerical ratio between the minimal erythemal dose with photoprotection and the minimal erythemal dose without photoprotection [157]. The compositions with photoprotective activity exhibited an SPF of approximately 30. Despite consumers’ search for photoprotective products with SPF > 30, values >15 provide adequate photoprotection when applied correctly [158].

Hence, the active compounds described in the patents exhibit sufficient SPF values; however, the claims for the use of extracts and isolated compounds limit the scale-up of these inventions. Additionally, the need for further studies to substantiate the photoprotective potential of *A. occidentale* and *A. muricata* places these inventions at an early stage of development, hindering technological transfer. On the other hand, natural photoprotective agents offer avenues for researchers and policymakers to develop sustainable, consumer-aligned formulations, fostering optimism about future innovations.

#### 3.5.6. Others

Other cosmetic activities are claimed in the patents. CN114515261 describes the use of a composition containing *A. squamosa* as a moisturizing agent [141]. KR102030419 employs an *A. muricata* extract to prevent gray hair [142]. FR3051363 describes the preparation of a cosmetic containing *A. muricata* [143]. JP6049830 uses an extract of *A. occidentale* to treat gray hair [144]. Finally, KR101957351 reported antioxidant properties of *A. occidentale* [145].

All described cosmetic activities of *Annona* genus plants may be linked to their antioxidant properties, as well documented in the literature [159,160,161]. Despite notable advances in research on *Annona* genus plants, further studies are needed to assess their moisturizing potential and effects on melanin synthesis, particularly their individual potential for dyeing gray hair, which remains poorly documented in the literature. Overall, the lack of validation and standardized tests indicates an early stage of development, underscoring the need for rigorous scientific validation and clinical studies to establish efficacy and safety, which are vital for researchers and regulatory agencies.

## 4. Challenges and the Current Market Scenario

In recent years, several events have threatened the survival of Brazilian biomes, including the Pantanal, which endured months of fires. These fires affected nearly one-third of the Pantanal’s area and resulted in biodiversity loss [162]. This scenario directly affects the scientific and technological aspects by reducing the number of species available for study and compromising the collection and exploitation of new candidates for therapeutic and cosmetic active ingredients. Thus, preservation policies for this biome are crucial for leveraging scientific and technological advances and for bringing new perspectives to the development of therapeutic and cosmetic products.

Furthermore, patenting products is often necessary to bring these innovations to market. However, this process is frequently hindered by bureaucracy. On one hand, most researchers lack the financial resources to invest in these procedures; on the other hand, the inventions are sometimes insufficiently detailed, hindering their clinical translation.

The patents studied in this work primarily relate to extracts. The patentability of natural-product-based inventions depends on the applicable jurisdiction and on whether the claimed subject matter meets the relevant patentability requirements, such as novelty, inventive step, and industrial applicability. Accordingly, inventions involving plant extracts may encompass specific compositions, processes, formulations, or uses that meet these requirements. Nonetheless, only a few formulations were patented with the explored species. This underscores the urgent need for technological advances in the patent development of natural products in proof-of-concept dosage forms, as a key means of facilitating market entry, which is crucial for industry stakeholders and policymakers.

Additionally, using technological formulations rather than extracts may facilitate the scale-up of products derived from biodiversity; however, this step requires considerable time and significant investment. From another perspective, this may represent an opportunity for the market to invest in developing new technologies for standardized phytopharmaceuticals, purified bioactive compounds, nanocarriers, controlled-release formulations, and validated products. This study identified unexplored areas for the pharmaceutical and cosmetic uses of complex formulations.

This scarcity may show a bottleneck existing in translating phytochemical discoveries into usable medicines. In addition, the patent landscape shows that biodiversity-based products involving Pantanal plants are still concentrated in basic technologies with low complexity, especially raw extracts. To advance studies on these plants, it is necessary to move toward more complex formulations; strengthening collaborations between academia and industry can help accelerate technology transfer. A limitation of the present analysis is the inclusion of granted patents only, which may underrepresent emerging technologies associated with recent or still-pending patent applications. Therefore, the findings should be interpreted as a landscape of granted technologies rather than a comprehensive representation of all patent activity involving these species.

In addition, this study discussed the potential of Pantanal plants by analyzing patents. Among the analyzed plants, Annonaceae is highlighted for its potential, including anticancer activity; however, the compound responsible is acetogenins, which show remarkable activity in tumor cells. This class of phytochemicals has limited use because of safety concerns. For instance, some acetogenins may inhibit mitochondrial complex I not only in cancer cells but also in healthy tissues; the toxicity of these molecules may be associated with their potential to inhibit tissue respiration processes [163]. In this scenario, future studies must be conducted with caution to ensure safety for clinical trials; developing delivery systems, optimizing doses, and conducting toxicological evaluations are also crucial steps.

Another safety issue concerns the use of *C. procera*; although this plant shows pharmacological activity, caution is also needed. This is because of toxicity from cardenolides and the plant’s latex constituents. Cardenolides interfere with Na^+^/K^+^-ATPase activity and may induce severe cardiotoxicity when improperly administered, whereas latex components may cause skin irritation, ocular injury, and allergic reactions [164,165]. Therefore, the pharmaceutical and cosmetic use of this species requires careful consideration of the plant part, extraction method, dose, and compositional variability, as these factors may directly influence both biological activity and toxicity. Extract purification, chemical standardization, and rigorous safety assessment are therefore essential to developing reproducible, safe formulations.

To address this issue, the pharmaceutical field has developed many formulations that reduce dosage and prevent adverse effects, such as nanotechnological formulations. The limited number of innovative compositions, such as nanotechnological delivery systems, represents a niche to explore for these plant species. These technological approaches can support the safe use of these plants and help explore their full potential.

Another important point is the lack of specification of the plant part used in some patents. Biological activity and safety may vary depending on the part used to obtain the extract. Leaves, bark, fruits, seeds, and latex can show different phytochemical profiles and thus, different therapeutic or toxic potentials.

## 5. Conclusions

This study mapped the technological landscape of therapeutic and cosmetic innovations based on plants found in Brazil’s Pantanal biome. Of the 58 patents identified, pharmacological and cosmetic applications were equally represented, highlighting the broad potential of Pantanal biodiversity for innovation. Despite this biome richness, most patents were filed by institutions in Asian countries, particularly South Korea and China, reflecting the technological gap and limited participation of Brazilian applicants, with only 1 patent. Overall, although patented technologies highlight the relevance of these natural products, most inventions fail to report experimental effects of most extracts under robust conditions. Nonetheless, the inventions do not describe independently validated efficacy for these products. This lack of experimental data and independent efficacy validation may reflect the large number of plant extracts, which are formulation ingredients but are rarely used on their own because of biopharmaceutical and pharmacological limitations.

However, the predominance of extract-based patents indicates an opportunity to advance formulation design and delivery systems, which are crucial to the clinical translation and market readiness of natural product-based technologies. Moving forward, future research should focus on developing standardized formulations, fostering university–industry collaborations, and establishing strategies for technology transfer and regulatory support. These actions will be key to transforming Pantanal’s biodiversity into tangible biotechnological and socioeconomic value.

## Data Availability

All the contributions of this study are included in the article. Further inquiries can be directed to the corresponding author.

## Supplementary Materials

The following supporting information can be downloaded at: https://www.mdpi.com/article/10.3390/plants15172632/s1, Prompt S1: Full prompt used for PubMed search of relevant species; Prompt S2: Example of the complete PubMed search strategy including species and synonyms; Table S1: Number of publications of each Pantanal plant found on PubMed; Table S2: Pantanal plants (from Flora e Funga do Brasil) with widely known synonyms; Table S3: Full dataset of the selected patents; Table S4: Phytochemicals present in the 5 plants described in the patents, along with the main bioactive compounds associated with their biological activity, according to the literature; Table S5: Level of evidence and active-ingredient composition reported for the patents included in the technological prospection. List S1: List of all International Patent Classification (IPC) codes found in the patents; References S1: Additional supporting references for chemical structures in Figure 8, Figure 9 and Figure 10.
