# Supplementary material for: Innovation and Biodiversity: Patent Trends in Therapeutic and Cosmetic Use of Scientifically Relevant Pantanal Plants
Source: Plants (Basel). 2026 Aug 28;15(17):2632. doi: 10.3390/plants15172632 (PMC13567684; doi:10.3390/plants15172632)
Supplement: Supplementary file 1 [file plants-15-02632-s001.zip › plants-4483674-supplementary.pdf]

Supplementary Material

Prompt S1: Full prompt model used for PubMed search of relevant species

"(("species"[Title/Abstract]) OR ("synonym"[Title/Abstract]) AND ("pharmacological activity"[Title/Abstract] OR "bioactivity"[Title/Abstract] OR "medicinal"[Title/Abstract] OR "biological activity"[Title/Abstract] OR "phytochemistry"[Title/Abstract]) NOT (review[Publication Type]) AND ("2015/01/01"[Date - Publication] : "2025/12/31"[Date - Publication])"

Prompt S2: Example of prompt used for PubMed search of species and synonyms

"(("Anacardium AND occidentale"[Title/Abstract]) OR ("Anacardium AND amilcarianum"[Title/Abstract]) OR ("Anacardium AND curatellaefolium "[Title/Abstract]) OR ("Anacardium AND kuhlmannianum"[Title/Abstract]) OR ("Anacardium AND microcarpum"[Title/Abstract]) OR ("Anacardium AND rondonianum"[Title/Abstract]) OR ("Anacardium AND othonianum"[Title/Abstract]) AND ("pharmacological activity"[Title/Abstract] OR "bioactivity"[Title/Abstract] OR "medicinal"[Title/Abstract] OR "biological activity"[Title/Abstract] OR "phytochemistry"[Title/Abstract]) NOT (review[Publication Type]) AND ("2015/01/01"[Date - Publication] : "2025/12/31"[Date - Publication])"

Table S1. Number of publications of each Pantanal plant found on PubMed

Plant	PubMed records
Hygrophila guianensis	1
Ruellia angustiflora	2
Ruellia ciliatiflora	2
Ruellia simplex	1
Thunbergia alata	2
Echinodorus cordifolius	5
Echinodorus floribundus	20
Echinodorus longiscapus	14
Echinodorus scaber	8
Hydrocleys nymphoides	1
Limnocharis flava	1
Limnocharis laforesti	1
Alternanthera brasiliana	10
Alternanthera philoxeroides	28
Alternanthera pungens	7
Alternanthera sessilis	25
Alternanthera tenella	5
Celosia argentea	44
Dysphania ambrosioides	89
Gomphrena celosioides	13
Hebanthe erianthos	9
Iresine diffusa	3
Pfaffia glomerata	30
Crinum americanum	3

Hippeastrum puniceum	7
Anacardium occidentale	165
Astronium urundeuva	36
Lithraea molleoides	2
Tapirira guianensis	9
Annona coriacea	7
Annona crassiflora	38
Annona emarginata	3
Annona montana	258
Annona muricata	235
Annona squamosa	96
Annona sylvatica	2
Annona tomentosa	2
Ammi visnaga	34
Cyclospermum leptophyllum	5
Asclepias curassavica	21
Aspidosperma australe	2
Aspidosperma quebracho-blanco	8
Aspidosperma triternatum	7
Calotropis procera	166
Mandevilla pohliana	1
Lemna aequinoctialis	26
Lemna minuta	1
Lemna valdiviana	3
Pistia stratiotes	40
Artemisia verlotiorum	4
Conyza bonariensis	13
Bidens pilosa	64
Cosmos sulphureus	7
Ageratum conyzoides	31
Campovassouria cruciata	1
Campuloclinium macrocephalum	1
Chromolaena odorata	43
Gymnocoronis spilanthoides	1
Mikania micrantha	24
Praxelis clematidea	3
Raulinoreitzia crenulata	1
Gamochaeta pensylvanica	1
Dimerostemma aspilioides	2
Eclipta prostrata	59
Sphagneticola trilobata	35
Acanthospermum hispidum	3
Acanthospermum australe	4
Tridax procumbens	32
Enydra fluctuans	2
Emilia fosbergii	1
Flaveria bidentis	1
Porophyllum ruderale	6
Tagetes minuta	17

Centratherum punctatum	2
Cyrtocymura scorpioides	3
Elephantopus mollis	51
Gymnanthemum amygdalinum	92
Basella alba	38
Amphilophium crucigerum	1
Amphilophium paniculatum	1
Cuspidaria convoluta	1
Dolichandra unguis-cati	1
Fridericia chica	27
Fridericia platyphylla	16
Handroanthus impetiginosus	30
Handroanthus serratifolius	4
Jacaranda cuspidifolia	2
Pyrostegia venusta	1
Tabebuia aurea	3
Tabebuia nodosa	1
Cochlospermum regium	3
Capsella bursa-pastoris	10
Ananas comosus	11
Bromelia balansae	1
Rhipsalis baccifera	3
Selenicereus setaceus	1
Trema micranthum	1
Canna indica	2
Caryocar brasiliense	7
Monteverdia ilicifolia	13
Ceratophyllum demersum	1
Combretum lanceolatum	1
Commelina benghalensis	2
Commelina diffusa	3
Commelina erecta	2
Tradescantia pallida	1
Ipomoea alba	1
Ipomoea aquatica	9
Ipomoea batatas	20
Ipomoea carnea	3
Ipomoea carnea	3
Turbina corymbosa	1
Costus arabicus	2
Costus spiralis	2
Ceratosanthes palmata	2
Luffa cylindrica"	7
Momordica charantia	51
Cyperus alternifolius	2
Cyperus brevifolius	1
Cyperus esculentus	2
Cyperus iria	3
Cyperus papyrus	4

Cyperus polystachyos	1
Cyperus rotundus	79
Eleocharis geniculata	1
Rhynchospora nervosa	1
Doliocarpus dentatus	2
Diospyros inconstans	1
Acalypha arvensis	2
Astraea gracilis	1
Astraea lobata	1
Croton bonplandianus	5
Euphorbia prostrata	6
Jatropha curcas	25
Jatropha elliptica	1
Ricinus communis	51
Abrus precatorius	22
Alysicarpus vaginalis	1
Amburana cearensis	8
Arachis hypogaea	14
Cajanus cajan	34
Cassia grandis	4
Cenostigma pluviosum	1
Cenostigma pluviosum	1
Centrosema pubescens	1
Chamaecrista nictitans	1
Chamaecrista rotundifolia	1
Crotalaria juncea	2
Desmodium incanum	2
Desmodium triflorum	1
Enterolobium contortisiliquum	1
Erythrina crista-galli	2
Galactia glaucescens	1
Hymenaea courbaril	4
Hymenaea stigonocarpa	1
Inga vera	1
Machaerium acutifolium	1
Neptunia oleracea	1
Parkinsonia praecox	1
Peltophorum dubium	2
Prosopis nigra	1
Pterocarpus santalinoides	1
Pterodon emarginatus	2
Pterodon pubescens	4
Rhynchosia minima	1
Senna alata	22
Senna occidentalis	23
Senna pendula	1
Stryphnodendron rotundifolium	4
Stylosanthes guianensis	1
Myriophyllum aquaticum	1

Heliotropium elongatum	1
Heliotropium indicum	8
Cantinoa mutabilis	2
Leonotis nepetifolia	6
Marsypianthes chamaedrys	1
Mesosphaerum suaveolens	9
Lindernia crustacea	2
Cuphea carthagenensis	3
Cuphea retrorsicapilla	8
Byrsonima crassifolia	2
Byrsonima intermedia	3
Diplopterys pubipetala	1
Malpighia glabra	2
Ceiba speciosa	3
Guazuma ulmifolia	4
Luehea divaricata	3
Luehea paniculata	1
Sida rhombifolia	1
Ficus crocata	1
Maclura tinctoria	1
Eugenia punicifolia	3
Urena lobata	3
Clidemia hirta	2
Brosimum acutifolium	1
Ficus adhatodifolia	1
Myrcia splendens	3
Syzygium	196
Boerhavia erecta	3
Victoria amazonica	1
Ludwigia longifolia	1
Ludwigia nervosa	1
Ludwigia octovalvis	1
Ludwigia sericea	1
Oxalis corniculata	10
Passiflora edulis	21
Moeroris amara	23
Phytolacca rivinoides	1
Piper cernuum	1
Piper nigrum	83
Piper tuberculatum	2
Angelonia minor	1
Scoparia dulcis	16
Bambusa vulgaris	8
Guadua angustifolia	2
Cynodon dactylon	18
Dactyloctenium aegyptium	5
Eleusine indica	7
Sporobolus indicus	1
Leersia hexandra	1

Oryza sativa	85
Oryza rufipogon	2
Andropogon leucostachyus	1
Axonopus compressus	1
Cenchrus brownii	1
Cenchrus echinatus	1
Cenchrus purpureus	6
Coix lacryma-jobi	25
Digitaria insularis	1
Echinochloa crus-galli	30
Elionurus muticus	2
Hymenachne amplexicaulis	1
Megathyrsus maximus	1
Melinis repens	1
Panicum repens	2
Paspalum vaginatum	1
Saccharum officinarum	5
Sorghum bicolor	22
Asemeia ovata	1
Senega paniculata	2
Polygonum hydropiperoides	1
Pontederia azurea	1
Pontederia crassipes	6
Sarcomphalus mistol	1
Borreria verticillata	2
Chiococca alba	2
Genipa americana	5
Geophila repens	2
Hamelia patens	6
Mitracarpus	3
Palicourea hoffmannseggiana	1
Palicourea tomentosa	2
Psychotria leiocarpa	1
Richardia brasiliensis	3
Zanthoxylum rhoifolium	2
Zanthoxylum riedelianum	2
Casearia sylvestris	12
Allophylus edulis	2
Cardiospermum halicacabum	12
Sapindus saponaria	6
Paullinia pinnata	10
Serjania marginata	2
Sideroxylon obtusifolium	3
Siparuna guianensis	6
Smilax fluminensis	8
Capsicum baccatum	4
Physalis angulata	20
Physalis pubescens	4
Solanum americanum	6

Solanum elaeagnifolium	1
Solanum sisymbriifolium	2
Solanum viarum	1
Sphenoclea zeylanica	1
Talinum fruticosum	6
Talinum paniculatum	7
Typha domingensis	5
Cecropia pachystachya	5
Aloysia virgata	1
Glandularia aristigera	1
Lantana camara	45
Lippia alba	21
Lippia origanoides	33
Anchietea pyrifolia	1
Cissus gongylodes	1
Cissus verticillata	2
Vochysia divergens	12
Qualea grandiflora	2
Bulnesia sarmientoi	2
Hedychium coronarium	13
Plagiochila disticha	1
Bryum	1
Octoblepharum albidum	1
Neckeropsis undulata	1
Araucaria angustifolia	5
Pteridium esculentum	1
Palhinhaea cernua	1
Nephrolepis biserrata	3
Niphidium crassifolium	1
Azolla filiculoides	2
Salvinia auriculata	1
Pityrogramma calomelanos	1
Christella dentata	1

Table S2. Pantanal plants (from Flora e Funga do Brasil) with widely known synonyms.

Plant	Synonym	Reference
Ruellia angustiflora	*Stephanophysum angustiflorum*	Ruellia in Flora e Funga do Brasil. Jardim Botânico do Rio de Janeiro. Available at:<https://floradobrasil.jbrj.gov.br/FB26252>. Access 08 ago. 2026
Echinodorus cordifolius	*Aquarius cordifolius*	Matias, L.Q.; Pestana, M.C.A. Alismataceae in Flora e Funga do Brasil. Jardim Botânico do Rio de Janeiro. Available at:<https://floradobrasil.jbrj.gov.br/FB608965>. Access 08 ago. 2026
Echinodorus floribundus	*Alisma floribundum*<br>*Aquarius floribundus*<br>*Echinodorus grandiflorus*<br>*Echinodorus macrophyllus*<br>*Echinodorus muricatus*<br>*Echinodorus punctatus*	Matias, L.Q.; Pestana, M.C.A. Alismataceae in Flora e Funga do Brasil. Jardim Botânico do Rio de Janeiro. Available at:<https://floradobrasil.jbrj.gov.br/FB39938>. Access 08 ago. 2026
Anacardium occidentale	*Anacardium amilcarianum*<br>*Anacardium curatellaefolium Anacardium kuhlmannianum*<br>*Anacardium microcarpum*<br>*Anacardium othonianum*<br>*Anacardium rondonianum*	Silva-Luz, C.L.; Pirani, J.R.; Pell, S.K.; Mitchell, J.D. Anacardiaceae in Flora e Funga do Brasil. Jardim Botânico do Rio de Janeiro. Available at:<https://floradobrasil.jbrj.gov.br/FB4381>. Access 08 ago. 2026
Eclipta prostrata	*Eclipta alba*	Alves, M.; Remor, D.; Bueno, V.R. Eclipta in Flora e Funga do Brasil. Jardim Botânico do Rio de Janeiro. Available at:<https://floradobrasil.jbrj.gov.br/FB16092>. Access 08 ago. 2026

Table S3. Full dataset of the selected patents. The table reports the final set of 58 patent documents included after application of the eligibility criteria. Patent bibliographic and classification data are reproduced from the raw dataset used for the technological prospection. Blank cells indicate information not available in the source dataset.

Plant	Patent title	Publication number used in manuscript	Raw publication number(s)	Raw publication date	Grant year	Inventor(s)	Applicant(s)	IPC code(s)	CPC code(s)	Application number	Application date	Priority number(s)
Calotropis procera (Aiton) W.T.Aiton	Compositions comprising dead sea extract and an extract of apple of sodom and uses thereof	US11400126 (B2)	US11400126 (B2); US2021393721 (A1)	23/12/2021	2022	PORTUGAL COHEN MEITAL [IL]; MAOR ZEEVI [IL]; ISH-SHALOM ELIRAN [IL]; COHEN DROR [IL]	AHAVA DEAD SEA LABORATORIES LTD [IL]	A61K8/96; A61K8/9789; A61K35/08; A61K36/24; A61P17/02; A61Q19/02; A61Q19/08	A61K8/965; A61K8/9789; A61K9/0014; A61K35/08; A61K36/24; A61P17/02; A61Q19/00; A61Q19/02; A61Q19/08; A61K9/0014	US201815733319	20181226	IL20170256648 20171228; WO2018IL51391 20181226
Calotropis procera (Aiton) W.T.Aiton	Calotropis procera extracts as anti-ulcerative colitis agents	US9533019 (B1)	US9533019 (B1)	03/01/2017	2017	AWAAD AMANI SHAFEEK [SA]; ZAIN GHADA MOHAMED [SA]; EL-MELIGY REHAM M [SA]; ALKANHAL HAYA FAHD [SA]; SESHADRI VIDYA DEVENATHADESIKAN [SA]	UNIV KING SAUD [SA]	A61K36/185; A61K36/27	A61K36/27; A61K2236/33	US201615059261	20160302	US201615059261 20160302
Anacardium occidentale L.	Anti-inflammatory botanical extract	EP3843762 (B1)	EP3843762 (A1); EP3843762 (B1)	07/07/2021	2022	RANA JATINDER [US]; MITCHELL KYLIE [US]	INNOPHOS INC [US]	A23L33/105; A61K36/22; A61P29/02	A23L33/105; A61K9/0014; A61K31/353; A61K36/22; A61P29/02	EP20190746311	20190712	US201862725461P 20180831; WO2019US41639 20190712
Anacardium occidentale L.	Gargle for relieving oral wound pain and preparation method thereof	CN112057387 (B)	CN112057387 (A); CN112057387 (B)	11/12/2020	2022	CHEN ZHEN; WANG HONGYUN	HAINAN HONGYI MEDICAL INSTR CO LTD	A61K8/65; A61K8/81; A61K8/9789; A61P1/02; A61P31/02; A61P31/04; A61Q11/00; A61Q17/00	A61K8/65; A61K8/8147; A61K8/9789; A61P1/02; A61P31/02; A61P31/04; A61Q11/00; A61Q17/...	CN20201159264	20200930	CN202011592 64 20200930
Annona squamosa L.	A pharmaceutical formulation for treating diabetes	IN5274/CHE/2013	-	-	2021	-	-	A61K 9/00	-	-	-	-

	mellitus and its associated complications											
Annona muricata L.	Pharmaceutical composition and health functional food comprising graviola leaf extract	KR102559349 (B1)	KR102559349 (B1); KR20220152476 (A)	16/11/2022	2023	LIM YUN SOOK; KIM SUN YEOU; LEE HAE JEE; SON YI SEUL; KIM JAE SUNG	UNIV INDUSTRY COOPERATION GROUP KYUNG HEE UNIV [KR]; UNIV GACHON IND ACAD COOP...	A23L33/105; A61K36/18; A61P3/10; A61P21/00	A23L33/105; A61K36/185; A61P3/10; A61P21/00; A23V2002/00; A23V2200/316; A23V2200/32...	KR20210059417	20210507	KR20210059417 20210507
Annona squamosa L.	Black sesame-sweetsop health-preserving wine and preparation method thereof	CN104293562 (B)	CN104293562 (A); CN104293562 (B)	21/01/2015	2016	CHEN LILIANG	ANHUI TIANXIAFU JIUYE CO LTD	C12G3/02; A61K35/28; A61K35/32; A61K35/34; A61K35/60; A61K36/899; A61P1/14; C12R1/865	A61K35/28; A61K35/32; A61K35/34; A61K35/60; A61K36/185; A61K36/28; A61K36/62; A61K36/734; A61K36/804; A61K36/82; A61K36/85; A61K36/896; A61K36/899; C12G3/02; A61K35/28; A61K2300/00; A61K35/32; A61K2300/00; A61K35/34; A61K2300/00; A61K35/60; A61K2300/00; A61K36/185; A61K2300/00; A61K36/28; A61K2300/00; A61K36/62; A61K2300/00; A61K36/734; A61K2300/00; A61K36/804; A61K2300/00; A61K36/82; A61K2300/00; A61K36/85;	CN201410457061	20140910	CN201410457061 20140910

									A61K2300/00; A61K36/896; A61K2300/00; A61K36/899; A61K2300/00			
Annona squamosa L.	Annona squamosa seed extract for treating choriocarcinoma, preparation method therefor and application thereof	CN109793732 (B)	WO2020156331 (A1)	06/08/2020	2021	WANG XIANGTAO [CN]; AO HUI [CN]; LI HAOWEN [CN]; WANG YI-AN [CN]; GUO YIFEI [CN]; HAN MEIHUA [CN]	THE INST OF MEDICINAL PLANT DEVELOPMENT [CN]	A61K31/365; A61K36/185; A61P35/00; C07D307/58	A61K31/365; A61K36/185; A61P35/00; C07D307/58	WO2020CN73269	20200120	CN2019109676 7 20190131
Annona squamosa L.	Compound with anti-tumor activity as well as preparation method and application thereof	CN113801000 (B)	CN113801000 (A); CN113801000 (B)	17/12/2021	2023	CHEN YAYUN	TAIZHOU POLYTECHNIC COLLEGE	A61K31/047; A61P35/00; C07C29/76; C07C29/86; C07C35/44	A61P35/00; C07C35/44; Y02A50/30	CN20211164296	20210910	CN2021116429 6 20210910
Annona squamosa L.	Enantio-kaurane diterpenoid compound with anti-tumor activity as well as preparation method and application of enantio-kaurane diterpenoid compound	CN114671759 (B)	CN114671759 (A); CN114671759 (B)	28/06/2022	2024	CHEN YAYUN; WU XINYI; LIN LING; CHEN HUAN	TAIZHOU OCCUPATIONAL TECH COLLEGE	A61K31/22; A61P35/00; C07C67/56; C07C69/16	A61P35/00; C07C69/16	CN202210312614	20220328	CN2022103126 14 20220328
Annona squamosa L.	Epoxy type annonaceous acetogenin compound with antitumor activity and preparation method and application thereof	CN106543159 (B)	CN106543159 (A); CN106543159 (B)	29/03/2017	2019	LI XIANG; MA CHENGYAO; CHEN JIANWEI	UNIV NANJING CHINESE MEDICINE	C07D407/14; A61K31/365; A61P35/00	C07D407/14; A61K36/185	CN201610988136	20161110	CN2016109881 36 20161110
Annona squamosa L.	M-bistetrahydrofuran annonaceous acetogenins compound with	CN106543117 (B)	CN106543117 (A); CN106543117 (B)	29/03/2017	2019	LI XIANG; MA CHENGYAO; CHEN JIANWEI	UNIV NANJING CHINESE MEDICINE	C07D307/58; A61K31/365; A61P35/00	C07D307/58	CN201610988132	20161110	CN2016109881 32 20161110

	anti-tumor activity and preparation method and application thereof											
Annona squamosa L.	O-bis-tetrahydrofuran type annonaceous acetogenins compound having antitumor activity, preparation method, and application thereof	CN106749124 (B)	CN106749124 (A); CN106749124 (B)	31/05/2017	2019	LI XIANG; MA CHENGYAO; CHEN JIANWEI	UNIV NANJING CHINESE MEDICINE	C07D307/58; A61K31/365; A61P35/00	C07D307/58	CN201610988134	20161110	CN2016109881 34 20161110
Annona squamosa L.	Sweetsop peel volatile oil extract with anti-tumor activity as well as preparation method and application thereof	CN106309516 (B)	CN106309516 (A); CN106309516 (B)	11/01/2017	2020	LI XIANG; CHEN YAYUN; CHEN JIANWEI	UNIV NANJING CHINESE MEDICINE	A61K36/185; A61P35/00; C11B1/10	A61K36/185; C11B1/10; A61K2236/37; A61K2236/53	CN201610943888	20161102	CN2016109438 88 20161102
Annona squamosa L.	Waste fruit peel extract composition with effect of treating gastric cancer	CN114558053 (B)	CN114558053 (A); CN114558053 (B)	31/05/2022	2022	WANG HONGBO; LIU YINGZHOU; AN ZHONGHUA; GONG YANLING	HOSPITAL FOR PEOPLE IN JIMO DISTR OF QINGDAO CITY; UNIV QINGDAO SCIENCE & TECHNOLOGY	A61K36/61; A61P35/00; A61P35/04	A61K36/185; A61K36/61; A61P35/00; A61P35/04; A61K2236/19; A61K2236/33 1; A61K2236/33 3; A61K2236/39; A61K2236/51; Y02A40/90; Y02A50/30; A61K36/185; A61K2300/00; A61K36/61; A61K2300/00	CN202210288912	20220322	CN2022102889 12 20220322
Annona squamosa L.	Composition for enhancing immunity as well	CN113244372 (B)	CN113244372 (A);	13/08/2021	2023	YUE YURONG; HAN YAHUI; YI BIN	JIANCHANGBAN G CHINESE HERBAL	A61K31/519; A61K36/815;	A61K31/519; A61K36/074; A61K36/185;	CN202110435276	20210422	CN2021104352 76 20210422

	as preparation technology and application thereof		CN113244372 (B)				MEDICINE CO LTD	A61K38/16; A61P37/04	A61K36/258; A61K36/815; A61K38/164; A61P37/04; Y02A50/30; A61K31/519; A61K2300/00; A61K36/074; A61K2300/00; A61K36/185; A61K2300/00; A61K36/258; A61K2300/00; A61K36/815; A61K2300/00; A61K38/164; A61K2300/00			
Annona muricata L.	Method for manufacturing graviola extract and pharmaceutical composition for immune enhancement	KR101951854 (B1)	KR101951854 (B1); KR20170140661 (A)	21/12/2017	2019	PARK TAE SIK [KR]; SHIM SOON MI [KR]; KIM GOON TAE [KR]	GACHON UNIV OF INDUSTRY-ACADEMIC COOPERATION FOUNDATION [KR]; UNIV SEJONG IND ...	A61K36/185	A23L33/105; A61K36/185; A61P37/00; A23V2002/00; A23V2200/324; A23V2250/21; A23V2300...	KR20160073255	20160613	KR20160073255 20160613
Anacardium occidentale L.	Composition containing Cistanche deserticola extract as well as preparation method and application of composition	CN118593682 (B)	CN118593682 (A); CN118593682 (B)	06/09/2024	2024	TIAN XIUJU; DONG XIAOGUANG	MEIKOU BIOLOGICAL PHARMACEUTICAL GUANGZHOU CO LTD	A23L33/105; A23L33/185; A61K36/64; A61K38/02; A61P37/04	A23L33/105; A23L33/185; A61K36/258; A61K36/64; A61K36/738; A61K38/02; A61P37/04; A23...	CN202410768250	20240614	CN202410768250 20240614
Annona squamosa L.	Extract composition with effects of protecting liver and lowering transaminase	CN114504615 (B)	CN114504615 (A); CN114504615 (B)	17/05/2022	2022	GONG YANLING; GONG YATING; WANG HONGBO; JIN HONG	UNIV QINGDAO SCIENCE & TECHNOLOGY	A61K36/77; A61P1/16	A61K36/185; A61K36/77; A61P1/16; A61K2236/15; A61K2236/17; A61K2236/19; A61K2236/331; A61K2236/333; A61K2236/39; A61K2236/51; A61K2236/53;	CN202210163590	20220222	CN202210163590 20220222

									A61K36/185; A61K2300/00; A61K36/77; A61K2300/00			
Annona muricata L.	Composition for treating hepatitis containing an extract of Cordia lutea flowers, Annona muricata leaves, and Curcuma longa roots	US9775871 (B2)	ES2546402 (T3)	23/09/2015	2015	CORAL JOSE GONZALO CABANILLAS	SABELL CORP [CA]; CORAL JOSE GONZALO CABANILLAS [PE]	A61K36/30; A61K36/57; A61K36/9066; A61P1/16; A61P31/12; A61P39/06	A61K36/30; A61K36/57; A61K36/9066; A61P1/16; A61P31/12; A61P31/14; A61P31/20; A61P39...	ES20080800424T	20081003	US20070977256P 20071003; WO2008CA01764 20081003
Annona squamosa L.	Pharmaceutical composition for treating or preventing nerve damage	CN110075274 (B)	CN110075274 (A); CN110075274 (B)	02/08/2019	2020	WANG TAO; WU HONGFU; YU MAOLIN; YU NA	UNIV GUANGDONG MEDICAL; THE THIRD AFFILIATED HOSPITAL OF GUANGDONG MEDICAL UNIV FOSHAN SHUNDE LONGJIANG HOSPITAL	A61K38/18; A61K31/7024; A61K36/886; A61K36/896; A61P25/00	A61K31/7024; A61K36/185; A61K36/28; A61K36/886; A61K36/896; A61K38/1866; A61P25/00; A61K2236/333; A61K31/7024; A61K2300/00; A61K36/185; A61K2300/00; A61K36/28; A61K2300/00; A61K36/886; A61K2300/00; A61K36/896; A61K2300/00; A61K38/1866; A61K2300/00	CN201910398309	20190514	CN201910398309 20190514
Annona muricata L.	Muscle activator	JP7209207 (B2)	JP2019026640 (A); JP7209207 (B2)	21/02/2019	2023	HAYASHI KENJIRO; LEE DAE-YEONG	NIPPON SHINYAKU CO LTD	A61K8/9789; A61K8/9794; A61K36/185; A61K36/23; A61K36/25; A61K36/282; A61K36/286; A6...	-	JP20180139871	20180726	JP20170146177 20170728
Anacardium occidentale L.	Amino derivatives of LCC as acetylcholinesterase inhibitors,	BR102013012924 (B1)	BR102013012924 (A2); BR102013012924 (B1)	13/01/2015	2023	ROMEIRO LUIZ ANTONIO SOARES; LEMES LAIS FLAVIA NUNES; RAMOS	UNIV BRASILIA FUNDACAO [BR]; UNIAO BRASILIENSE DE EDUCACAO E	A61K31/137; A61P25/00; A61P25/16; C07D207/08; C07D211/32;	-	BR20131012924	20130524	BR20131012924 20130524

	processes for their production, pharmaceutical compositions containing them and applications					GISELLE DE ANDRADE; ...	CULTURA [BR]; UNIV...	C07D211/40; C07D241/04; C0...				
Anacardium occidentale L.	Health maintaining and kidney tonifying Chinese chestnut baijiu and preparation method thereof	CN105733899 (B)	CN105733899 (A); CN105733899 (B)	06/07/2016	2018	PENG HAIYING	CHEN JUNJIE	C12G3/04; A61K35/644; A61K36/8969; A61P13/12	A61K35/36; A61K35/644; A61K36/07; A61K36/074; A61K36/076; A61K36/185; A61K36/22; A61...	CN20161094957	20160219	CN2016109495 7 20160219
Anacardium occidentale L.	A herbal cream preparation that cauterizes and removes condyloma acuminata on genital warts and the likes	PH1/2013/00010 8 (B1)	PH1201300010 8 (A1); PH1201300010 8 (B1)	25/05/2015	2015	DELA CRUZ ROMMEL B [PH]	DELA CRUZ ROMMEL B [PH]	A61K36/22; A61K9/06; A61K36/31; A61K36/752; A61P17/00; A61P17/12; A61P31/00; A61P31/...	-	PH20131000108	20130415	PH2013100010 8 20130415
Anacardium occidentale L.	Plant extract pharmaceutical composition for improving secretion of endogenous insulin-like growth factor 1	CN117257891 (B)	CN117257891 (A); CN117257891 (B)	22/12/2023	2024	LIU YUNYUN; LIU SICUN; WEN XUEJUN; WANG ZHENG; ZHAO DONGLAI; SONG WEIBIN; WANG JIAL...	REENGINEERING REGENERATION HEALTH TECH HANGZHOU CO LTD	A23L19/00; A23L25/00; A23L31/00; A23L33/10; A23L33/105; A23L33/125; A61K31/7016; A61...	A23L19/09; A23L25/30; A23L31/00; A23L33/10; A23L33/105; A23L33/125; A61K31/7016; A61...	CN20231063899	20230113	CN2023106389 9 20230113
Annona montana Macfad.	Annona montana Macf leaves extract for anti-oxidant, anti-inflammation, whitening, repairing and anti-wrinkle	TWI774339 (B)	TW202241482 (A); TWI774339 (B)	01/11/2022	2022	LIANG CHIA-HUA [TW]; CHAN LEONG-PERNG [MY]; CHEN PIN-JU [TW]; LIU CHIA-CHI [TW]	CHIA NAN UNIV OF PHARMACY & SCIENCE [TW]	A23L33/105; A61K8/9789; A61K36/185; A61P17/00; A61P29/00; A61Q19/00	-	TW20210114930	20210426	TW2021011493 0 20210426
Annona squamosa L.	Anti-aging composition for skin external application comprising annona squamosa placenta culture extracts	KR101619709 (B1)	KR101619709 (B1); KR2015009088 4 (A)	06/08/2015	2016	MOH SANG HYUN [KR]; LEE JEONG HUN [KR]; JUNG HAE SOO [KR]; SONG MI YOUNG [KR]; KIM SOO YUN [KR]; MOH JI HONG [KR]	BIO FD & C [KR]	A61K8/97; A61K36/185; A61Q19/02; A61Q19/08	-	KR20150105532	20150727	KR2015010553 2 20150727

Annona squamosa L.	Cosmetic containing custard apple multiple fermentation liquor and preparation method thereof	CN113041200 (B)	CN113041200 (A); CN113041200 (B)	29/06/2021	2021	HE QIUXING; LUO TINGTING; KONG XIANGLING; HUANG JUNZI; YI XIAOMEI; CAO HUA	UNIV GUANGDONG PHARM	A61K8/9789; A61Q19/00; A61Q19/02; A61Q19/08; C12P1/02; C12P1/04; C12P39/00; C12R1/01; C12R1/125; C12R1/225; C12R1/865	A61K8/9789; A61Q19/00; A61Q19/02; A61Q19/08; C12P1/02; C12P1/04; C12P39/00; A61K2800/10; A61K2800/782; A61K2800/85	CN202110384568	20210409	CN202110384568 20210409
Annona squamosa L.	Use of Annona squamosa L. Peel extract in preparation of composition for inhibiting matrix metalloproteinase, hyaluronidase, lipoxygenase activity and nitric oxide generation	TWI814026 (B)	TW202302130 (A); TWI814026 (B)	16/01/2023	2023	LEU KUEN-LIN [TW]; CHANG CHUN-LIN [TW]	CHIA NAN UNIV OF PHARMACY & SCIENCE [TW]	A61K8/9789; A61K36/185; A61P43/00; A61Q19/08	-	TW20210124108	20210630	TW20210124108 20210630
Annona muricata L.	Application of Annona squamosa leavening in preparation of composition	CN111956536 (B)	CN111956536 (A); CN111956536 (B)	20/11/2020	2022	LIN YUNG HSIANG; LI SHAOYU	TCI BIO TECH SHANGHAI CO LTD	A23L19/00; A23L29/00; A61K8/9789; A61K36/185; A61P17/00; A61P29/00; A61P37/04; A61P3...	A23L19/00; A23L29/065; A61K8/9789; A61K36/185; A61P17/00; A61P29/00; A61P37/04; A61P...	CN202010429465	20200520	US201962849996P 20190520
Annona muricata L.	Cosmetic composition	KR102436816 (B1)	KR102436816 (B1); KR20220027092 (A)	07/03/2022	2022	LEE HO KYU [KR]	LEE HO KYU [KR]	A61K8/9783; A61Q19/00; A61Q19/02; A61Q19/08	A61K8/9783; A61Q19/00; A61Q19/02; A61Q19/08; A61K2800/522	KR20220002831	20220107	KR20140078896 20140626; KR20220002831 20220107
Annona muricata L.	Cosmetic composition containing fermented graviola for improving anti-wrinkle and method of preparing the same	KR101648148 (B1)	KR101648148 (B1)	12/08/2016	2016	CHOUNG EUI SU [KR]; YUN JAE UNG [KR]; PARK MIN HEE [KR]; KIM HONG BON [KR]; JOUNG...	DANJOUNGBIO CO LTD [KR]; ENBIOSCIENCE CO LTD [KR]; JOUNG YONG JAE [KR]	A61K8/97; A61K8/99; A61Q19/08	-	KR20150101359	20150717	KR20150101359 20150717

Annona muricata L.	Cosmetic composition containing the extraction mixture	KR101999885 (B1)	KR101999885 (B1); KR20180105308 (A)	28/09/2018	2019	CHA WOONG HO [KR]; JIN SUN MI [KR]; KIM MIN JI [KR]; KIM JIN HWI [KR]	HIMEDICOS INC [KR]	A61K8/97; A61K8/60; A61Q19/00	A61K8/60; A61K8/9755; A61K8/9789; A61Q19/00; A61K2800/59	KR20170032178	20170315	KR20170032178 20170315
Annona muricata L.	Cosmetic Composition for Anti-oxidation Anti-inflammatory and Anti-wrinkling Comprising Enzyme-treated Extracts of Annona uricata and Method for Manufacturing the Same	KR101868507 (B1)	KR101868507 (B1)	19/06/2018	2018	SONG CHANG SEOB; RYU MA YOUNG; JO JAE EUNG	HAPPYBIO CO LTD [KR]	A61K8/9789; A61Q19/00; A61Q19/08	A61K8/9789; A61Q19/00; A61Q19/08; A61K2800/85	KR20170135984	20171019	KR20170135984 20171019
Annona muricata L.	Cosmetic Composition for Silver Generation for Improving Skin Winkles and maskpack Sheet using the same	KR101774408 (B1)	KR101774408 (B1)	04/09/2017	2017	CHOI WAN [KR]	CHOI WAN [KR]	A61K8/97; A61K8/02; A61Q19/08	A61K8/0212; A61K8/97; A61Q19/08	KR20170058091	20170510	KR20170058091 20170510
Anacardium occidentale L.	Botanical Extract for Skin Care	US10967022 (B2)	US10967022 (B2); US2020069754 (A1)	05/03/2020	2021	RANA JATINDER [US]; MITCHELL KYLIE [US]	INNOPHOS INC [US]	A61K8/9789; A61K36/22; A61P17/18; A61P29/00	A23L33/105; A61K8/9789; A61K36/22; A61P17/00; A61P17/02; A61P17/18; A61P29/00; A61Q1...	US201916515097	20190718	US201862725441P 20180831; US201916515097 20190718
Anacardium occidentale L.	Cosmetic composition including as effective ingredient an nanoemulsion of cashew seed oil	KR102726643 (B1)	KR102726643 (B1); KR20230046487 (A)	06/04/2023	2024	MOON KWON KI; HONG JUN YOUNG; LEE CHAE EUN; IM DA BEEN	VICHE COSMETIC CO LTD [KR]	A61K8/06; A61K8/368; A61K8/92; A61K8/9789; A61Q19/08	A61K8/068; A61K8/368; A61K8/922; A61K8/9789; A61Q19/08; A61K2800/21	KR20210129538	20210930	KR20210129538 20210930
Anacardium occidentale L.	Cosmetic composition for improving skin wrinkle anti-inflammation and moisturizing	KR102693360 (B1)	KR102693360 (B1)	08/08/2024	2024	KIM EUN MI; KIM MIN SOO; SONG SI YOUNG; LEE YONG MI; LEE KWANG YOUNG; YOO AH YOUNG...	B&B KOREA CO LTD [KR]	A61K8/9789; A61Q19/00; A61Q19/08	-	KR20230161258	20231120	KR20230161258 20231120

	containing the complex extracts of Morus Bombycis Leaf, Polygonum Fagopyrum Seed, Moringa Oleifera Seed and Anacardium Occidentale											
Anacardium occidentale L.	Botanical antioxidants	EP3843557 (B1)	EP3843557 (A1); EP3843557 (B1)	07/07/2021	2023	RANA JATINDER [US]; MITCHELL KYLIE [US]	INNOPHOS INC [US]	A23L33/105; A61K36/22; A61P39/06	A23L33/105; A61K31/353; A61K36/22; A61K36/77; A61P39/06; A23V2002/00; A23V2002/00; A...	EP20190746305	20190712	US201862725435P 20180831; WO2019US41608 20190712
Annona muricata L.	2 manufacturing method of hair loss prevention shampoo containing graviola component extracted in two steps	KR102143154 (B1)	KR102143154 (B1); KR20200042783 (A)	24/04/2020	2020	HONG YUN HEE [KR]	HONG YUN HEE [KR]	A61K8/9783; A61Q5/02; A61Q7/00	A61K8/9783; A61Q5/02; A61Q7/00; A61K2800/82	KR20180123382	20181016	KR20180123382 20181016
Annona muricata L.	Fermented product of medical herbs for preventing, ameliorating, or protecting against hair loss, and use thereof	KR102585336 (B1)	WO2024112111 (A1)	30/05/2024	2023	BYUN YOUNG MI [KR]; KIM JUNG GIL [KR]; LEE JE HOON [KR]; KIM SEUNG KOOK [KR]	BYUN YOUNG MI [KR]; KIM JUNG GIL [KR]; LEE JE HOON [KR]; KIM SEUNG KOOK [KR]	A61K8/9761; A61K8/9789; A61K8/9794; A61Q7/00	A61K8/9761; A61K8/9789; A61K8/9794; A61Q7/00	WO2023KR18938	20231122	KR20220157612 20221122
Annona muricata L.	Method for producing natural extract with increased alopecia prevention effect and shampoo for preventing alopecia containing natural extract	KR101540333 (B1)	KR101540333 (B1)	29/07/2015	2015	HWANG IN SOON [KR]	HWANG IN SOON [KR]	A61K8/97; A61K8/02; A61Q5/02; A61Q7/00	-	KR20150060198	20150429	KR20150060198 20150429

	produced by same method											
Annona muricata L.	Shampoo comprising the extract of graviola for improving condition of scalp and preventing hair loss	KR102080412 (B1)	KR102080412 (B1)	21/02/2020	2020	LEE JEONG MIN [KR]	LEE JEONG MIN [KR]	A61K8/9783; A61K8/64; A61K8/92; A61Q5/02; A61Q7/00; A61Q19/00	A61K8/64; A61K8/922; A61K8/9783; A61Q5/02; A61Q7/00; A61Q19/00	KR20180145106	20181122	KR2018014510 6 20181122
Annona muricata L.	Use of bioactive compounds of Annona muricata ferment for preparing a composition of inhibiting melanin production and enhancing anti-oxidation capacity of THP-1 cells	TWI817363 (B)	TW202333760 (A); TWI817363 (B)	01/09/2023	2023	LIN YUNG-HSIANG [TW]; LIN HUAN-YOU [TW]	TCI CO LTD [TW]	A23L33/105; A61K8/9789; A61K31/05; A61K31/192; A61K31/216; A61K31/7004; A61K36/185; ...	-	TW20220106654	20220223	TW2022010665 4 20220223
Annona muricata L.	Composition comprising Graviola callus lysate extract as an active ingredient having skin whitening antioxidant skin condition improvement and hair loss inhibitory effects	KR102676679 (B1)	KR102676679 (B1); KR102676679 (B9)	25/06/2024	2024	JUNG DAI HYUN; KIM SU JUNG; KIM SEONGSIM; CHU SO JEONG; SUNG JI YEON; LIM CHANG IL...	BIO FD&C [KR]	A61K8/9789; A61K36/185; A61P17/00; A61P17/02; A61P17/14; A61Q7/00; A61Q19/00; A61Q19...	-	KR20230094673	20230720	KR2023009467 3 20230720
Anacardium occidentale L.	Whitening composition	CN118512378 (B)	CN118512378 (A); CN118512378 (B)	20/08/2024	2025	GAO LIJIE; XIE YONG; HE RUI; FENG CHEN; LIU WENLI; LI JINXIAO	ZHIWU YISHENG GUANGDONG BIOTECHNOLOG Y CO LTD; BEIJING DR PLANT BIOTECHNOLOG Y C...	A61K8/9789; A61K8/9794; A61Q19/00; A61Q19/02; A61Q19/08	-	CN202410719759	20240605	CN2024107197 59 20240605
Annona muricata L.	Cosmetic compositions	KR101844673 (B1)	KR101844673 (B1);	05/03/2018	2018	LEE YOON JIN [KR]; JANG KYOUNG HEE	COSMOCOS CO LTD [KR]	A61K8/97; A61K8/14;	A61K8/14; A61K8/922;	KR20160106151	20160822	KR2016010615 1 20160822

	containing niosomes of graviola extract		KR2018002150 3 (A)			[KR]; JEON AE JI [KR]; LEE HWAN JIN [KR]; KIM ...		A61K8/92; A61Q19/00	A61K8/9789; A61Q19/00; A61K2800/41 3			
Annona muricata L.	Mask cosmetic composition comprising Graviola antibiotic extract for skin anti-wrinkle and method of preparing the same	KR102475897 (B1)	KR102475897 (B1); KR2017004751 4 (A)	08/05/2017	2022	이호규,; 정철희	이호규	A61K8/02; A61K8/30; A61K8/97; A61Q19/00; A61Q19/08	-	KR20150147725	20151023	KR2015014772 5 20151023
Annona muricata L.	Skin vital rising cosmetic composition comprising Graviola antibiotic extract and method of preparing the same	KR102475898 (B1)	KR102475898 (B1); KR2017004751 5 (A)	08/05/2017	2022	이호규,; 정철희	이호규	A61K8/02; A61K8/30; A61K8/97; A61Q19/00	-	KR20150147727	20151023	KR2015014772 7 20151023
Anacardium occidentale L.	Moisturizing, anti-wrinkle, nourishing and skin-care gel and preparation method thereof	CN113368026 (B)	CN113368026 (A); CN113368026 (B)	10/09/2021	2024	SUN QI; LI ANQI; LI SHENGLI	LI ANQI	A61K8/31; A61K8/34; A61K8/37; A61K8/72; A61K8/73; A61K8/92; A61K8/9789; A61K8/9794; A...	A61K8/31; A61K8/345; A61K8/37; A61K8/72; A61K8/73; A61K8/922; A61K8/925; A61K8/9789; ...	CN202110881156	20210802	CN2021108811 56 20210802
Annona muricata L.	Cosmetic composition for preventing and improving skin damage caused by ultraviolet rays and method for manufacturing the same	KR102679087 (B1)	KR102679087 (B1)	27/06/2024	2024	KIM CHAN O	AB PARTNERS CO LTD [KR]	A61K8/365; A61K8/67; A61K8/73; A61K8/9789; A61Q17/04; A61Q19/00	-	KR20230186091	20231219	KR2023018609 1 20231219
Anacardium occidentale L.	Compounds having excited state intramolecular proton transfer	AU2020234097 (B2)	WO202018342 2 (A1)	17/09/2020	2023	OPATZ TILL [DE]; KÜHLBORN JONAS [DE]; DE KONING CHARLES B [ZA];	UNIV JOHANNESBURG WITWATERSRAN D [ZA]; JOHANNES	A61K31/351; A61P17/00; C07D251/14; C07D309/12	A61K8/35; A61K8/49; A61K8/4966; A61K8/498; A61Q17/04;	WO2020IB52254	20200312	ZA2019000151 4 20190312

	(esipt) character for use in treating and/or preventing sunburn and/or preventing u.v. damage					NGWIRA KENNEDY J...	GUTENBERG UNIV [DE]		C07C65/40; C07D251/14; C07D271...			
Annona squamosa L.	Male conditioning essence and preparation method thereof	CN114515261 (B)	CN114515261 (A); CN114515261 (B)	20/05/2022	2023	XUE BIN'E; ZHANG HUI; LI YONGQUAN; GUO WEI; LIAO BAIYONG; WANG YI	GUTTA ARMOURED AGRICULTURAL ENG COLLEGE	A61K8/34; A61K8/35; A61K8/49; A61K8/63; A61K8/64; A61K8/67; A61K8/73; A61K8/92; A61K8/9789; A61Q19/00	A61K8/345; A61K8/35; A61K8/4973; A61K8/63; A61K8/645; A61K8/675; A61K8/73; A61K8/735; A61K8/922; A61K8/9789; A61Q19/00; Y02A50/30	CN20221026959	20220111	CN2022102695 9 20220111
Annona muricata L.	A composition comprising graviola having inhibitory effect on induction of grey hair and positive effect on induction of black hair	KR102030419 (B1)	KR102030419 (B1); KR2019006364 9 (A)	10/06/2019	2019	KIM MOON MOO; YUNGHEE OH; LEE KYUWON; JANG JIWON; YOON HYESOO; HA YEJIN; JEON SOJUN...	UNIV DONG EUI IND ACAD COOP FOUND [KR]	A23L33/105; A61K8/9783; A61K8/9789; A61K8/9794; A61Q5/00; A61Q7/00	A23L33/105; A61K8/9783; A61K8/9789; A61K8/9794; A61Q5/00; A61Q7/00; A23V2002/00; A23...	KR20170162586	20171130	KR2017016258 6 20171130
Annona muricata L.	Preparation d'une levure acetobacter et sa mise en evidence	FR3051363 (B3)	FR3051363 (A3); FR3051363 (B3)	24/11/2017	2018	COMBET JEAN FELIX [FR]	COMBET JEAN FELIX [FR]	A61K8/97; A61Q5/00	A61K8/9789; A61Q5/00	FR20160000801	20160519	FR2016000080 1 20160519
Anacardium occidentale L.	Hair dye and hair dyeing method	JP6049830 (B2)	JP2015232031 (A); JP6049830 (B2)	24/12/2015	2016	YAMADA MINORU; MIKOSHIBA KAZUFUMI; MANJI RYOSUKE	SHISEI CHEMICAL KK	A61K8/97; A61K8/34; A61K8/40; A61Q5/10	-	JP20150168558	20150828	JP20150168558 20150828
Anacardium occidentale L.	Natural cosmetic composition using aronia	KR101957351 (B1)	KR101957351 (B1)	12/03/2019	2019	JANG HAE DONG; LEE DAE KUN; KIM JI HYE; KIM CHEOL WOO; PARK JI WON	UNIV HANNAM INST IND ACAD COOP [KR]	A61K8/97; A61K8/06; A61Q19/00; A61Q19/02	A61K8/06; A61K8/9789; A61Q19/00	KR20170143139	20171031	KR2017014313 9 20171031

Symbol hyphen(-) : absence of retrieved information. For CPC, many patent offices have used only IPCs for a period of time and changed their policies over time.

Table S4. Phytochemicals present in the 5 plants described in the patents, along with the main bioactive compounds associated with their biological activity, according to the literature.

Plant	Part	Secondary metabolites	Bioactive compounds	Biological activity	Ref
A. montana	Leaf	Alkaloids, sterols, and acetogenins	Annonacin	Antitumor, cytotoxic	[1]
	Seeds	Acetogenins	Annonacin	Antitumor	
	Stem	Phenolic compounds	Gallic acid, catechin	Antitumor, antiparasitic	
A. muricata	Leaf	Acetogenins	Annonacin	Antitumor	[2]
	Leaf & seed	Acetogenins	Annonacin	Antitumor	
	Fruits	Acetogenins, alkaloids	Annonacin	Antitumor	
	Root	Acetogenins	Annonacin	Antitumor	
A. squamosa	Leaf	Alkaloids	Anonaine	Antimicrobial	[1][3]
	Bark	Diterpenes, acetogenins	Squamocin, bullatacin	Antitumor	
	Pulp fruit	Monoterpenes	Limonene, α-pinene	Antioxidant, anti-inflammatory, antimicrobial	
	Stem	Diterpenes, alkaloids	Anonaine	Antimicrobial	
	Seed	Acetogenins	Squamocin, annonacin	Antitumor, cytotoxic	
C. procera	Leaves	Flavonoids, fatty acid ethyl esters, palmitic acid esters, linoleic acids, and amino acids	Rutin, kaempferol, Isorhamnetin, Tridecyl ester	Antioxidant, anti-inflammatory	[4-7]
	Fruits	Terpenoids, cardenolides, flavonoids	Proceraside, calotropenol, calotroprocerol A, uscharidin	Antitumor, HIF-1 Inhibitory Activity	
	Seeds	Cardenolides, terpenoids, flavonoids	Reevesioside J, boistroside, patuletin	Antitumor	
	Latex	Cardenolides	Calotropin, calotropenol	Antitumor	
A. occidentale	Leaves	Flavonoids, monoterpenes	Limonene, sabinene,	Antioxidant, anti-inflammatory, antimicrobial	[8]
	Cashew nut shell liquid	Phenolic compounds	Anacardic acid, cardanol, cardol	Antimicrobial, anti-inflammatory, anti-tumor	

Legend: *Plant parts, secondary metabolites, and bioactive compounds were restricted to their involvement in the extracts or formulations described in the included patents. This summary does not represent the full range of secondary metabolite groups, bioactive compounds, and plant parts for each species, as there is no further detail on the phytochemistry of the specimens in the included patents.* ***Reference list for Table S2:*** *[1] Al Kazman, B.S.M.; Harnett, J.E.; Hanrahan, J.R. Traditional Uses, Phytochemistry and Pharmacological Activities of Annonacae. Molecules 2022, 27, 3462, doi:https://doi.org/10.3390/molecules27113462. [2] Badrie, N.; Schauss, A.G. Chapter 39 - Soursop (Annona muricata L.): Composition, Nutritional Value, Medicinal Uses, and Toxicology. In Bioactive Foods in Promoting Health, Watson, R.R., Preedy, V.R., Eds.; Academic Press: San Diego, 2010; pp. 621-643. [3] Yassin, S.; Elsohafy, S.M.; El-Hawiet, A.; Abdel-Kader, M.S.; Ghareeb, D.A.; Darwish, F.A.; Amer, M.E. Comparative phytochemical and pharmacological analysis of two cultivars of Annona squamosa L. cultivated in Egypt. npj Sci. Food. 2025, 9, 8, doi:https://doi.org/10.1038/s41538-*

024-00368-6. [4] El-Banna, A.A.; Ibrahim, R.S.; Ghareeb, D.A.; Nassief, S.M. Unveiling the metabolic profile and anti-inflammatory potential of apple of Sodom (Calotropis procera (Aiton) W.T.) by UPLC–MS/MS chemometric analysis. Sci. Rep. 2026, 16, 5402, doi:https://doi.org/10.1038/s41598-025-33542-1. [5] Kaur, A.; Batish, D.R.; Kaur, S.; Chauhan, B.S. An Overview of the Characteristics and Potential of Calotropis procera From Botanical, Ecological, and Economic Perspectives. Front. Plant Sci. 2021, 12, 690806, doi:https://doi.org/10.3389/fpls.2021.690806. [6] Wadhwani, B.D.; Mali, D.; Vyas, P.; Nair, R.; Khandelwal, P. A review on phytochemical constituents and pharmacological potential of Calotropis procera. RSC Adv. 2021, 11, 35854-35878, doi:https://doi.org/10.1039/D1RA06703F. [7] Alvarado-Sansininea, J.J.; Sánchez-Sánchez, L.; López-Muñoz, H.; Escobar, M.L.; Flores-Guzmán, F.; Tavera-Hernández, R.; Jiménez-Estrada, M. Quercetagetin and Patuletin: Antiproliferative, Necrotic and Apoptotic Activity in Tumor Cell Lines. Molecules 2018, 23, 2579, doi:https://doi.org/10.3390/molecules23102579. [8] Salehi, B.; Gültekin-Özgüven, M.; Kırkın, C.; Özçelik, B.; Morais-Braga, M.F.B.; Carneiro, J.N.P.; Bezerra, C.F.; Silva, T.G.d.; Coutinho, H.D.M.; Amina, B.; et al. Anacardium Plants: Chemical,Nutritional Composition and Biotechnological Applications. Biomolecules 2019, 9, 465, doi:https://doi.org/10.3390/biom9090465.

Table S5. Level of evidence and active-ingredient composition reported for the patents included in the technological prospection. Evidence level refers to the type of experimental evidence reported in the patent document, not to independent validation of efficacy. Active-ingredient composition indicates whether the patent describes one or multiple active ingredients. When the available information did not allow reliable classification, the entry was recorded as 'Not reported' or 'Not clearly reported' rather than inferred.

Plant	Publication number	Reported evidence	Active-ingredient composition
Calotropis procera (Aiton) W.T.Aiton	US11400126 (B2)	Not reported	Multiple active ingredients
Calotropis procera (Aiton) W.T.Aiton	US9533019 (B1)	In vivo	Single active ingredient
Anacardium occidentale L.	EP3843762 (B1)	In vitro	Single active ingredient
Anacardium occidentale L.	CN112057387 (B)	Clinical: Human volunteers	Not clearly reported
Annona squamosa L.	IN5274/CHE/2013	In vitro	Multiple active ingredients
Annona muricata L.	KR102559349 (B1)	In vitro	Single active ingredient
Annona squamosa L.	CN104293562 (B)	Not reported	Multiple active ingredients
Annona squamosa L.	CN109793732 (B)	In vitro	Single active ingredient
Annona squamosa L.	CN113801000 (B)	In vitro	Single active ingredient
Annona squamosa L.	CN114671759 (B)	In vitro	Single active ingredient
Annona squamosa L.	CN106543159 (B)	In vitro	Single active ingredient
Annona squamosa L.	CN106543117 (B)	In vitro	Single active ingredient
Annona squamosa L.	CN106749124 (B)	In vitro	Single active ingredient
Annona squamosa L.	CN106309516 (B)	In vitro	Single active ingredient
Annona squamosa L.	CN114558053 (B)	In vitro	Multiple active ingredients
Annona squamosa L.	CN113244372 (B)	In vivo	Multiple active ingredients
Annona muricata L.	KR101951854 (B1)	In vitro	Single active ingredient
Anacardium occidentale L.	CN118593682 (B)	In vitro	Multiple active ingredients
Annona squamosa L.	CN114504615 (B)	In vivo	Multiple active ingredients
Annona muricata L.	US9775871 (B2)	Clinical: Human volunteers	Multiple active ingredients
Annona squamosa L.	CN110075274 (B)	In vivo	Multiple active ingredients
Annona muricata L.	JP7209207 (B2)	In vitro	Not clearly reported
Anacardium occidentale L.	BR102013012924 (B1)	Not reported	Single active ingredient
Anacardium occidentale L.	CN105733899 (B)	In vivo	Multiple active ingredients
Anacardium occidentale L.	PH1/2013/000108 (B1)	Not reported	Multiple active ingredients

Anacardium occidentale L.	CN117257891 (B)	In vivo	Multiple active ingredients
Annona montana Macfad.	TWI774339 (B)	Not clearly reported	Single active ingredient
Annona squamosa L.	KR101619709 (B1)	In vitro	Single active ingredient
Annona squamosa L.	CN113041200 (B)	Clinical: Human volunteers	Single active ingredient
Annona squamosa L.	TWI814026 (B)	Not clearly reported	Single active ingredient
Annona muricata L.	CN111956536 (B)	Clinical: Human volunteers	Single active ingredient
Annona muricata L.	KR102436816 (B1)	In vitro	Multiple active ingredients
Annona muricata L.	KR101648148 (B1)	In vitro	Single active ingredient
Annona muricata L.	KR101999885 (B1)	Clinical: Human volunteers	Multiple active ingredients
Annona muricata L.	KR101868507 (B1)	Clinical: Human volunteers	Single active ingredient
Annona muricata L.	KR101774408 (B1)	Clinical: Human volunteers	Multiple active ingredients
Anacardium occidentale L.	US10967022 (B2)	In vitro	Single active ingredient
Anacardium occidentale L.	KR102726643 (B1)	Clinical: Human volunteers	Single active ingredient
Anacardium occidentale L.	KR102693360 (B1)	In vitro	Multiple active ingredients
Anacardium occidentale L.	EP3843557 (B1)	In vitro	Single active ingredient
Annona muricata L.	KR102143154 (B1)	Clinical: Human volunteers	Multiple active ingredients
Annona muricata L.	KR102585336 (B1)	Clinical: Human volunteers	Multiple active ingredients
Annona muricata L.	KR101540333 (B1)	Clinical: Human volunteers	Multiple active ingredients
Annona muricata L.	KR102080412 (B1)	Clinical: Human volunteers	Multiple active ingredients
Annona muricata L.	TWI817363 (B)	In vitro	Single active ingredient
Annona muricata L.	KR102676679 (B1)	In vitro	Single active ingredient
Anacardium occidentale L.	CN118512378 (B)	Not clearly reported	Multiple active ingredients
Annona muricata L.	KR101844673 (B1)	In vitro	Single active ingredient
Annona muricata L.	KR102475897 (B1)	In vitro	Single active ingredient
Annona muricata L.	KR102475898 (B1)	In vitro	Single active ingredient
Anacardium occidentale L.	CN113368026 (B)	Clinical: Human volunteers	Single active ingredient
Annona muricata L.	KR102679087 (B1)	In vitro	Single active ingredient
Anacardium occidentale L.	AU2020234097 (B2)	Not reported	Multiple active ingredients
Annona squamosa L.	CN114515261 (B)	Clinical: Human volunteers	Multiple active ingredients
Annona muricata L.	KR102030419 (B1)	In vitro	Multiple active ingredients
Annona muricata L.	FR3051363 (B3)	Not reported	Not clearly reported
Anacardium occidentale L.	JP6049830 (B2)	Not reported	Not clearly reported
Anacardium occidentale L.	KR101957351 (B1)	Not reported	Multiple active ingredients

List S1: List of all International Patent Classification (IPC) codes found in the patents

A61K35/08: Medicinal preparations containing materials or reaction products thereof with undetermined constitution: Mineral waters; Sea water.

A61K36/24: Medicinal preparations of undetermined constitution containing material from algae, lichens, fungi or plants, or derivatives thereof, e.g. traditional herbal medicines Apocynaceae (Dogbane family), e.g. plumeria or periwinkle.

A61K8/96: Cosmetics or similar toiletry preparations containing materials, or derivatives thereof of undetermined constitution.

A61K8/9789: Cosmetics or similar toiletry preparations: Magnoliopsida [dicotyledons].

A61P17/02: Drugs for dermatological disorders for treating wounds, ulcers, burns, scars, keloids, or the like.

A61Q19/02: Specific use of cosmetics or similar toiletry preparations: Preparations for care of the skin for chemically bleaching or whitening the skin.

A61Q19/08: Specific use of cosmetics or similar toiletry preparations: Preparations for care of the skin Anti-ageing preparations.

A61K36/185: Medicinal preparations of undetermined constitution containing material from algae, lichens, fungi or plants, or derivatives thereof, e.g. traditional herbal medicines: Magnoliopsida [dicotyledons].

A61K36/27: Medicinal preparations of undetermined constitution containing material from algae, lichens, fungi or plants, or derivatives thereof, e.g. traditional herbal medicines: Asclepiadaceae (Milkweed family), e.g. hoya.

A23L33/105: Modifying nutritive qualities of foods; Dietetic products; Preparation or treatment thereof using additives (addition of substantially indigestible substances, Plant extracts, their artificial duplicates or their derivatives.

A61K36/22: Medicinal preparations of undetermined constitution containing material from algae, lichens, fungi or plants, or derivatives thereof, e.g. traditional herbal medicines: Anacardiaceae (Sumac family), e.g. smoketree, sumac or poison oak.

A61P29/02: Specific therapeutic activity of chemical compounds or medicinal preparations: Non-central analgesic, antipyretic or antiinflammatory agents, e.g. antirheumatic agents; Non-steroidal antiinflammatory drugs [NSAID] without antiinflammatory effect.

A61K8/65: Cosmetics or similar toiletry preparations characterised by the composition containing organic compounds, Proteins; Peptides; Derivatives or degradation products thereof: Collagen; Gelatin; Keratin; Derivatives or degradation products thereof.

A61K8/81: Cosmetics or similar toiletry preparations characterised by the composition containing organic macromolecular compounds obtained by reactions involving only carbon-to-carbon unsaturated bonds.

A61P1/02: Drugs for disorders of the alimentary tract or the digestive system Stomatological preparations, e.g. drugs for caries, aphtae, periodontitis.

A61P31/02: Antiinfectives, i.e. antibiotics, antiseptics, chemotherapeutics Local antiseptics.

A61P31/04: Antiinfectives, i.e. antibiotics, antiseptics, chemotherapeutics: Antibacterial agents.

A61Q11/00: Preparations for care of the teeth, of the oral cavity or of dentures; Dentifrices, e.g. toothpastes; Mouth rinses.

A61Q17/00: Specific use of cosmetics or similar toiletry preparations: Barrier preparations; Preparations brought into direct contact with the skin for affording protection against external influences, e.g. sunlight, X-rays or other harmful rays, corrosive materials, bacteria or insect stings.

A61K 9/00: Medicinal preparations characterised by special physical form.

A61K36/18: Medicinal preparations of undetermined constitution containing material from algae, lichens, fungi or plants, or derivatives thereof, e.g. traditional herbal medicines antigens from pollen: Magnoliophyta (angiosperms).

A61P21/00: Drugs for disorders of the muscular or neuromuscular system.

A61P3/10: Drugs for disorders of the metabolism (of the blood or the extracellular fluid for glucose homeostasis (pancreatic hormones) for hyperglycaemia, e.g. antidiabetics.

A61K31/7034: Medicinal preparations containing organic active ingredients.

Carbohydrates; Sugars; Derivatives thereof (sorbitol) Compounds having saccharide radicals attached to non-saccharide compounds by glycosidic linkages attached to a carbocyclic compound, e.g. phloridzin.

A61K31/7048: Medicinal preparations containing organic active ingredients Carbohydrates; Sugars; Derivatives thereof (sorbitol) Compounds having saccharide radicals and heterocyclic rings having oxygen as a ring hetero atom, e.g. leucoglucosan, hesperidin, erythromycin, nystatin, digitoxin or digoxin.

A61P1/16: Drugs for disorders of the alimentary tract or the digestive system for liver or gallbladder disorders, e.g. hepatoprotective agents, cholagogues, litholytics.

A61P3/00: Drugs for disorders of the metabolism.

A61P3/04: Drugs for disorders of the metabolism: Anorexiants; Antiobesity agents.

A61P3/06: Drugs for disorders of the metabolism (of the blood or the extracellular fluid A61P7/00): Antihyperlipidemics.

C11B11/00: Animal or vegetable oils, fats, fatty substances or waxes; fatty acids therefrom; detergents; candles producing, e.g. by pressing raw materials or by extraction from waste materials, refining or preserving fats, fatty substances, e.g. lanolin, fatty oils or waxes; essential oils; perfumes (drying-oils C09F): Recovery or refining of other fatty substances, e.g. lanolin or waxes: Lanolin; Woolfat.

A61K35/28: Medicinal preparations containing materials or reaction products thereof with undetermined constitution. Materials from mammals; Compositions comprising non-specified tissues or cells; Compositions comprising non-embryonic stem cells; Genetically modified cells (vaccines or medicinal preparations containing antigens or antibodies A61K39/00): Bone marrow; Haematopoietic stem cells; Mesenchymal stem cells of any origin, e.g. adipose-derived stem cells.

A61K35/32: Medicinal preparations containing materials or reaction products thereof with undetermined constitution. Materials from mammals; Compositions comprising non-specified tissues or cells; Compositions comprising non-embryonic stem cells; Genetically modified cells (vaccines or medicinal preparations containing antigens or antibodies); Bones; Osteocytes; Osteoblasts; Tendons; Tenocytes; Teeth; Odontoblasts; Cartilage; Chondrocytes; Synovial membrane.

A61K35/34: Medicinal preparations containing materials or reaction products thereof with undetermined constitution. Materials from mammals; Compositions comprising non-specified tissues or cells; Compositions comprising non-embryonic stem cells; Genetically modified cells (vaccines or medicinal preparations containing antigens or antibodies); Muscles; Smooth muscle cells; Heart; Cardiac stem cells; Myoblasts; Myocytes; Cardiomyocytes (vascular smooth muscle).

A61K35/60: Medicinal preparations containing materials or reaction products thereof with undetermined constitution. Materials from animals other than mammals. Fish, e.g. seahorses; Fish eggs

A61K36/899: Medicinal preparations of undetermined constitution containing material from algae, lichens, fungi or plants, or derivatives thereof, e.g. traditional herbal medicines (antigens from pollen); Poaceae or Gramineae (Grass family), e.g. bamboo, corn or sugar cane.

A61P1/14: Drugs for disorders of the alimentary tract or the digestive system Prodigestives, e.g. acids, enzymes, appetite stimulants, antidyspeptics, tonics, antiflatulents.

C12G3/02: Biochemistry; beer; spirits; wine; vinegar; microbiology; enzymology; mutation or genetic engineering; Wine; preparation thereof; alcoholic beverages (beer c12c); preparation of alcoholic beverages not provided for in subclasses C12C OR C12H. Preparation of other alcoholic beverages by fermentation.

C12R1/865: Biochemistry; beer; spirits; wine; vinegar; microbiology; enzymology; mutation or genetic engineering indexing scheme associated with subclasses c12c - c12q, relating to microorganisms: Saccharomyces cerevisiae.

A61K31/365: Medicinal preparations containing organic active ingredients Heterocyclic compounds having oxygen as the only ring hetero atom, e.g. fungichromin: Lactones.

A61P35/00: Specific therapeutic activity of chemical compounds or medicinal preparations: Antineoplastic agents.

C07D307/58: Heterocyclic compounds containing five-membered rings having one oxygen atom as the only ring hetero atom not condensed with other rings having two or three double bonds between ring members or

between ring members and non-ring members with hetero atoms or with carbon atoms having three bonds to hetero atoms with at the most one bond to halogen, e.g. ester or nitrile radicals, directly attached to ring carbon atoms One oxygen atom, e.g. butenolide.

A61K31/047: Medicinal preparations containing organic active ingredients Hydroxy compounds, e.g. alcohols; Salts thereof, e.g. alcoholates having two or more hydroxy groups, e.g. sorbitol.

C07C29/76: Preparation of compounds having hydroxy or O-metal groups bound to a carbon atom not belonging to a six-membered aromatic ring. Separation; Purification; Use of additives, e.g. for stabilization by physical treatment.

C07C29/86: Preparation of compounds having hydroxy or O-metal groups bound to a carbon atom not belonging to a six-membered aromatic ring. Separation; Purification; Use of additives, e.g., for stabilization, by physical treatment. by liquid-liquid treatment.

C07C35/44: Compounds having at least one hydroxy or O-metal group bound to a carbon atom of a ring other than a six-membered aromatic ring polycyclic, at least one hydroxy group bound to a condensed ring system, with a hydroxy group on a condensed ring system having more than three rings.

A61K31/22: Medicinal preparations containing organic active ingredients; Esters, e.g., nitroglycerine, selenocyanates of carboxylic acids of acyclic acids, e.g. pravastatin.

C07C67/56: Preparation of carboxylic acid esters. Separation; Purification; Stabilisation; Use of additives by solid-liquid treatment; by chemisorption.

C07C69/16: Esters of carboxylic acids; Esters of carbonic or haloformic acids (ortho esters, see the relevant groups, e.g., C07C43/32). Esters of acyclic saturated monocarboxylic acids having the carboxyl group bound to an acyclic carbon atom or to hydrogen. Acetic acid esters of dihydroxylic compounds.

C07D407/14: Heterocyclic compounds containing two or more hetero rings, at least one ring having oxygen atoms as the only ring hetero atoms, not provided for by group.

C07D405/00 containing three or more hetero rings.

C11B1/10: Production of fats or fatty oils from raw materials by extracting.

A61K36/61: Medicinal preparations of undetermined constitution containing material from algae, lichens, fungi or plants, or derivatives thereof, e.g. traditional herbal medicines (antigens from pollen A61K39/36). Myrtaceae (Myrtle family), e.g. teatree or eucalyptus.

A61P35/04: Antineoplastic agents specific for metastasis.

A61K31/519: Medicinal preparations containing organic active ingredients. Heterocyclic compounds having nitrogen as a ring hetero atom, e.g. guanethidine or rifamycins having six-membered rings with two or more nitrogen atoms as the only ring heteroatoms, e.g. piperazine or tetrazines takes precedence; with three nitrogen atoms; Pyrimidines; Hydrogenated pyrimidines, e.g. trimethoprim. ortho- or peri-condensed with heterocyclic rings.

A61K36/815: Medicinal preparations of undetermined constitution containing material from algae, lichens, fungi or plants, or derivatives thereof, e.g. traditional herbal medicines (antigens from pollen A61K39/36). Lycium (desert-thorn).

A61K38/16: Medicinal preparations containing peptides (peptides containing beta-lactam rings. A61K31/00; cyclic dipeptides not having in their molecule any other peptide link than those which form their ring, e.g. piperazine-2,5-diones, ergot alkaloids of the cyclic peptide type containing macromolecular compounds having statistically distributed amino acid units.

A61K31/74: Medicinal preparations containing antigens or antibodies characterised by the non-active ingredients, e.g. peptides as drug carriers). Peptides having more than 20 amino acids; Gastrins; Somatostatins; Melanotropins; Derivatives thereof (enzyme inhibitors).

A61P37/04: Drugs for immunological or allergic disorders. Immunomodulators. Immunostimulants.

A23L33/185: Modifying nutritive qualities of foods; Dietetic products; Preparation or treatment thereof using additives (addition of substantially indigestible substances) Amino acids, peptides or proteins. Vegetable proteins.

A61K36/64: Medicinal preparations of undetermined constitution containing material from algae, lichens, fungi or plants, or derivatives thereof, e.g. traditional herbal medicines (antigens from pollen). Orobanchaceae (Broom-rape family).

A61K38/02: Medicinal preparations containing peptides (peptides containing beta-lactam rings. A61K31/00; cyclic dipeptides not having in their molecule any other peptide link than those which form their ring, e.g. piperazine-2,5-diones, ergot alkaloids of the cyclic peptide type containing macromolecular compounds having statistically distributed amino acid units.

A61K31/74: medicinal preparations containing antigens or antibodies characterized by the non-active ingredients, e.g. peptides as drug carriers). Peptides of undefined number of amino acids; Derivatives thereof.

A61K36/77: Medicinal preparations of undetermined constitution containing material from algae, lichens, fungi or plants, or derivatives thereof, e.g. traditional herbal medicines (antigens from pollen). Sapindaceae (Soapberry family), e.g. lychee or soapberry.

A61K36/30: Medicinal preparations of undetermined constitution containing material from algae, lichens, fungi or plants, or derivatives thereof, e.g. traditional herbal medicines (antigens from pollen). Boraginaceae (Borage family), e.g. comfrey, lungwort or forget-me-not.

A61K36/57: Medicinal preparations of undetermined constitution containing material from algae, lichens, fungi or plants, or derivatives thereof, e.g. traditional herbal medicines (antigens from pollen). Magnoliaceae (Magnolia family).

A61K36/9066: Medicinal preparations of undetermined constitution containing material from algae, lichens, fungi or plants, or derivatives thereof, e.g. traditional herbal medicines (antigens from pollen). Curcuma, e.g. common turmeric, East Indian arrowroot or mango ginger.

A61P31/12: Antiinfectives, i.e. antibiotics, antiseptics, chemotherapeutics. Antivirals.

A61P39/06: Specific therapeutic activity of chemical compounds or medicinal preparations. General protective or antinoxious agents. Free radical scavengers or antioxidants.

A61K31/7024: Medicinal preparations containing organic active ingredients. Carbohydrates; Sugars; Derivatives thereof (sorbitol). Esters of saccharides.

A61K36/886: Medicinal preparations of undetermined constitution containing material from algae, lichens, fungi or plants, or derivatives thereof, e.g. traditional herbal medicines (antigens from pollen). Aloeaceae (Aloe family), e.g. aloe vera.

A61K36/896: Medicinal preparations of undetermined constitution containing material from algae, lichens, fungi or plants, or derivatives thereof, e.g. traditional herbal medicines (antigens from pollen). Liliaceae (Lily family), e.g. daylily, plantain lily, Hyacinth or narcissus.

A61K38/18: Medicinal preparations containing peptides (peptides containing beta-lactam rings; cyclic dipeptides not having in their molecule any other peptide link than those which form their ring, e.g. piperazine-2,5-diones, ergot alkaloids of the cyclic peptide type containing macromolecular compounds having statistically distributed amino acid units .

A61K31/74: Medicinal preparations containing antigens or antibodies.

A61K39/00; medicinal preparations characterised by the non-active ingredients, e.g. peptides as drug carriers. Peptides having more than 20 amino acids; Gastrins; Somatostatins; Melanotropins; Derivatives thereof (enzyme inhibitors) from animals; from humans (enzyme inhibitors). Growth factors; Growth regulators.

A61P25/00: Drugs for disorders of the nervous system.

A61K36/428: Medicinal preparations of undetermined constitution containing material from algae, lichens, fungi or plants, or derivatives thereof, e.g. traditional herbal medicines (antigens from pollen) – Trichosanthes.

A61P17/00: Drugs for dermatological disorders.

A61P21/06: Drugs for disorders of the muscular or neuromuscular system. Anabolic agents (androgens).

A61K31/137: Medicinal preparations containing organic active ingredients. Amines having aromatic rings, e.g. ketamine, nortriptyline (methadone A61K31/137). Arylalkylamines, e.g. amphetamine, epinephrine, salbutamol, ephedrine, or methadone.

A61P25/16: Drugs for disorders of the nervous system for treating abnormal movements, e.g. chorea, dyskinesia. Anti-Parkinson drugs.

C07D207/08: Heterocyclic compounds containing five-membered rings not condensed with other rings, with one nitrogen atom as the only ring hetero atom with only hydrogen or carbon atoms directly attached to the

ring nitrogen atom, having no double bonds between ring members or between ring members and non-ring members with hydrocarbon radicals, substituted by hetero atoms, attached to ring carbon atoms.

C07D211/32: Heterocyclic compounds containing hydrogenated pyridine rings, not condensed with other rings with only hydrogen or carbon atoms directly attached to the ring nitrogen atom having no double bonds between ring members or between ring members and non-ring members with hydrocarbon or substituted hydrocarbon radicals directly attached to ring carbon atoms with substituted hydrocarbon radicals attached to ring carbon atoms with hydrocarbon radicals, substituted by doubly bound oxygen or sulfur atoms or by two oxygen or sulfur atoms singly bound to the same carbon atom by oxygen atoms.

C07D211/40: Heterocyclic compounds containing hydrogenated pyridine rings, not condensed with other rings with only hydrogen or carbon atoms directly attached to the ring nitrogen atom having no double bonds between ring members or between ring members and non-ring members with hetero atoms or with carbon atoms having three bonds to hetero atoms with at the most one bond to halogen, e.g. ester or nitrile radicals, directly attached to ring carbon atoms. Oxygen atoms.

C07D241/04: Heterocyclic compounds containing 1,4-diazine or hydrogenated 1,4-diazine rings not condensed with other rings having no double bonds between ring members or between ring members and non-ring members.

C07D269/00: Heterocyclic compounds having nitrogen and oxygen as the only ring hetero atoms. Heterocyclic compounds containing rings having one nitrogen atom and one oxygen atom as the only ring hetero atoms according to more than one of groups.

C07D261/00 - C07D267/00: Heterocyclic compounds containing rings of more than six members having one nitrogen atom and one oxygen atom as the only ring hetero atoms.

C07D283/00: Heterocyclic compounds containing rings having one nitrogen atom and one sulfur atom as the only ring hetero atoms, according to more than one of groups.

C07D275/00 - C07D281/00: Heterocyclic compounds containing rings of more than six members having one nitrogen atom and one sulfur atom as the only ring hetero atoms.

A61K35/644: Medicinal preparations containing materials or reaction products thereof with undetermined constitution. Materials from animals other than mammals. Arthropods (aquatic crustaceans A61K35/612). Insects, e.g. bees, wasps or fleas. Beeswax; Propolis; Royal jelly; Honey.

A61K36/8969: Medicinal preparations of undetermined constitution containing material from algae, lichens, fungi or plants, or derivatives thereof, e.g. traditional herbal medicines (antigens from pollen A61K39/36). Polygonatum (Solomon's seal).

A61P13/12: Drugs for disorders of the urinary system of the kidneys.

C12G3/04: Preparation of other alcoholic beverages by mixing, e.g. for preparation of liqueurs.

A61K36/31: Medicinal preparations of undetermined constitution containing material from algae, lichens, fungi or plants, or derivatives thereof, e.g. traditional herbal medicines (antigens from pollen A61K39/36). Brassicaceae or Cruciferae (Mustard family), e.g. broccoli, cabbage or kohlrabi.

A61K36/752: Medicinal preparations of undetermined constitution containing material from algae, lichens, fungi or plants, or derivatives thereof, e.g. traditional herbal medicines (antigens from pollen). Citrus, e.g. lime, orange or lemon.

A61K9/06: Medicinal preparations characterised by special physical form (nuclear magnetic resonance contrast preparations or magnetic resonance imaging contrast preparations; preparations containing radioactive substances). Ointments; Bases therefor; Other semi-solid forms, e.g. creams, sticks, gels (composition of ointments, creams or gels).

A61P17/12: Drugs for dermatological disorders. Keratolytics, e.g. wart or anti-corn preparations.

A61P31/00: Antiinfectives, i.e. antibiotics, antiseptics, chemotherapeutics.

A61P31/20: Antiinfectives, i.e. antibiotics, antiseptics, chemotherapeutics for DNA viruses.

A23L19/00: Products from fruits or vegetables; Preparation or treatment thereof (marmalades, jams, jellies or the like A23L21/10; treating harvested fruit or vegetables in bulk A23N).

A23L25/00: Food consisting mainly of nutmeat or seeds; Preparation or treatment thereof.

A23L31/00: Edible extracts or preparations of fungi; Preparation or treatment thereof.

A23L33/10: Modifying nutritive qualities of foods; Dietetic products; Preparation or treatment thereof using additives (addition of substantially indigestible substances).

A23L33/125: Modifying nutritive qualities of foods; Dietetic products; Preparation or treatment thereof using additives (addition of substantially indigestible substances) containing carbohydrate syrups; containing sugars; containing sugar alcohols; containing starch hydrolysates (indigestible substances).

A61K31/7016: Medicinal preparations containing organic active ingredients. Carbohydrates; Sugars; Derivatives thereof (sorbitol). Disaccharides, e.g. lactose, lactulose (lactobionic acid).

A61P1/00: Drugs for disorders of the alimentary tract or the digestive system.

A61P19/10: Drugs for skeletal disorders for bone diseases, e.g. rachitism, Paget's disease for osteoporosis.

A61P25/28: Drugs for disorders of the nervous system for treating neurodegenerative disorders of the central nervous system, e.g. nootropic agents, cognition enhancers, drugs for treating Alzheimer's disease or other forms of dementia.

A61P29/00: Non-central analgesic, antipyretic or antiinflammatory agents, e.g. antirheumatic agents; Non-steroidal antiinflammatory drugs [NSAID].

A61P39/00: General protective or antinoxious agents.

A61P9/10: Drugs for disorders of the cardiovascular system for treating ischaemic or atherosclerotic diseases, e.g. antianginal drugs, coronary vasodilators, drugs for myocardial infarction, retinopathy, cerebrovascular insufficiency, renal arteriosclerosis..

A61Q19/00: Preparations for care of the skin.

A61K8/97: Cosmetics or similar toiletry preparations characterised by the composition containing materials, or derivatives thereof of undetermined constitution from algae, fungi, lichens or plants; from derivatives thereof .

C12P1/02: Preparation of compounds or compositions, not provided for in groups by using microorganisms or enzymes by using fungi.

C12P1/04: Preparation of compounds or compositions, not provided for in groups by using microorganisms or enzymes by using bacteria.

C12P39/00: Processes involving microorganisms of different genera in the same process, simultaneously .

C12R1/01: Microorganisms; Processes using microorganisms.

C12R1/125: Microorganisms; Processes using microorganisms: Bacillus subtilis ; Hay bacillus; Grass bacillus.

C12R1/225: Microorganisms; Processes using microorganisms: Lactobacillus.

A61P43/00: Drugs for specific purposes, not provided for in groups A61P1/00-A61P41/00.

A23L29/00: Foods or foodstuffs containing additives containing additives for modifying the nutritive qualities; containing substantially indigestive additives, e.g. dietary fibres, A23L33/21; containing additives for preservation; Preparation or treatment thereof.

A61K8/9783; Cosmetics or similar toiletry preparations characterised by the composition containing materials, or derivatives thereof of undetermined constitution from algae, fungi, lichens or plants; from derivatives thereof: Angiosperms [Magnoliophyta].

A61K8/99: Cosmetics or similar toiletry preparations characterised by the composition containing materials, or derivatives thereof of undetermined constitution from microorganisms other than algae or fungi, e.g. protozoa or bacteria.

A61K8/60: Cosmetics or similar toiletry preparations characterised by the composition containing organic compounds: Sugars; Derivatives thereof.

A61K8/02: Cosmetics or similar toiletry preparations characterised by special physical form.

A61P17/18: Drugs for dermatological disorders: Antioxidants, e.g. antiradicals (preparations for protection against sunlight).

A61K8/06: Cosmetics or similar toiletry preparations characterised by special physical form Dispersions: Emulsions.

A61K8/368: Cosmetics or similar toiletry preparations characterised by the composition.

containing organic compounds containing oxygen Carboxylic acids; Salts or anhydrides thereof with carboxyl groups directly bound to carbon atoms of aromatic rings.

A61K8/92: Cosmetics or similar toiletry preparations characterised by the composition; Oils, fats or waxes; Derivatives thereof, e.g. hydrogenation products thereof.

A61Q5/02: Preparations for care of the hair. Preparations for repairing the hair, e.g. hair cure.

A61Q7/00: Preparations for affecting hair growth.

A61K8/9761: Cosmetics or similar toiletry preparations characterised by the composition containing materials, or derivatives thereof of undetermined constitution from algae, fungi, lichens or plants; from derivatives thereof Gymnosperms [Coniferophyta] Cupressaceae [Cypress family], e.g. juniper or cypress.

A61K8/9794: Cosmetics or similar toiletry preparations characterised by the composition containing materials, or derivatives thereof of undetermined constitution from algae, fungi, lichens or plants; from derivatives thereof Angiosperms [Magnoliophyta] Liliopsida [monocotyledons].

A61K8/64: Cosmetics or similar toiletry preparations characterised by the composition containing organic compounds Proteins; Peptides; Derivatives or degradation products thereof.

A61K31/05: Medicinal preparations containing organic active ingredients Hydroxy compounds, e.g. alcohols; Salts thereof, e.g. alcoholates Phenols (cannabinoids A61K31/658).

A61K31/192: Medicinal preparations containing organic active ingredients Acids; Anhydrides, halides or salts thereof, e.g. sulfur acids, imidic, hydrazonic or hydroximic acids (hydroxamic acids A61K31/16; peroxy acids A61K31/327) Carboxylic acids, e.g. valproic acid (salicylic acid A61K31/60) having aromatic groups, e.g. sulindac, 2-aryl-propionic acids, ethacrynic acid (cannabinoids A61K31/658).

A61K31/216: Medicinal preparations containing organic active ingredients Esters, e.g. nitroglycerine, selenocyanates of carboxylic acids of acids having aromatic rings, e.g. benactizyne, clofibrate.

A61K31/7004: Medicinal preparations containing organic active ingredients Carbohydrates; Sugars; Derivatives thereof (sorbitol A61K31/047) Monosaccharides having only carbon, hydrogen and oxygen atoms.

A61P17/14: Drugs for dermatological disorders for baldness or alopecia.

A61K8/14: Cosmetics or similar toiletry preparations characterised by special physical form Liposomes; Vesicles.

A61K8/30: Cosmetics or similar toiletry preparations characterised by the composition containing organic compounds.

A61K8/31: Cosmetics or similar toiletry preparations characterised by the composition containing organic compounds Hydrocarbons.

A61K8/34: Cosmetics or similar toiletry preparations characterised by the composition containing organic compounds containing oxygen Alcohols.

A61K8/37: Cosmetics or similar toiletry preparations characterised by the composition containing organic compounds containing oxygen Esters of carboxylic acids.

A61K8/72: Cosmetics or similar toiletry preparations characterised by the composition containing organic macromolecular compounds.

A61K8/73: Cosmetics or similar toiletry preparations characterised by the composition containing organic macromolecular compounds Polysaccharides.

A61K8/365: Cosmetics or similar toiletry preparations characterised by the composition containing organic compounds containing oxygen Carboxylic acids; Salts or anhydrides thereof Hydroxycarboxylic acids; Ketocarboxylic acids.

A61K8/67: Cosmetics or similar toiletry preparations characterised by the composition containing organic compounds Vitamins.

A61Q17/04: Barrier preparations; Preparations brought into direct contact with the skin for affording protection against external influences, e.g. sunlight, X-rays or other harmful rays, corrosive materials, bacteria or insect stings Topical preparations for affording protection against sunlight or other radiation; Topical sun tanning preparations.

A61K31/351: Medicinal preparations containing organic active ingredients Heterocyclic compounds having oxygen as the only ring hetero atom, e.g. fungichromin having six-membered rings with one oxygen as the only ring hetero atom not condensed with another ring.

C07D251/14: Heterocyclic compounds containing 1,3,5-triazine rings not condensed with other rings having three double bonds between ring members or between ring members and non-ring members with hydrogen or carbon atoms directly attached to at least one ring carbon atom.

A61K8/35: Cosmetics or similar toiletry preparations characterised by the composition containing organic compounds containing oxygen Ketones, e.g. benzophenone.

A61K8/49: Cosmetics or similar toiletry preparations characterised by the composition containing organic compounds containing heterocyclic compounds.

A61K8/63: Cosmetics or similar toiletry preparations characterised by the composition containing organic compounds Steroids; Derivatives thereof.

A61Q5/00: Preparations for care of the hair.

A61K8/19: Cosmetics or similar toiletry preparations characterised by the composition containing inorganic ingredients.

A61K8/27: Cosmetics or similar toiletry preparations characterised by the composition containing inorganic ingredients Zinc; Compounds thereof .

A61K8/41: Cosmetics or similar toiletry preparations characterised by the composition containing organic compounds containing nitrogen (quinones containing nitrogen A61K8/355) Amines.

A61K8/42: Cosmetics or similar toiletry preparations characterised by the composition containing organic compounds containing nitrogen (quinones containing nitrogen A61K8/355) Amides.

A61K8/44: Cosmetics or similar toiletry preparations characterised by the composition containing organic compounds containing nitrogen (quinones containing nitrogen A61K8/355) Aminocarboxylic acids or derivatives thereof, e.g. aminocarboxylic acids containing sulfur; Salts; Esters or N-acylated derivatives thereof.

A61K8/46: Cosmetics or similar toiletry preparations characterised by the composition containing organic compounds containing sulfur (A61K8/44 takes precedence).

A61Q5/10: Preparations for care of the hair. Preparations for permanently dyeing the hair.

References S1: Additional Supporting References for Chemical Structures in Figures 8, 9 and 10.

Figure 8:

1. Aguilar-Hernández, G.; Zepeda-Vallejo, L.G.; García-Magaña, M.L.; Vivar-Vera, M.A.; Pérez-Larios, A.; Girón-Pérez, M.I.; Coria-Téllez, A.V.; Rodríguez-Aguayo, C.; Montalvo-González, E. Extraction of alkaloids using ultrasound from pulp and by-products of soursop fruit (*Annona muricata* L.). *Appl. Sci.* **2020**, *10*, 4869. https://doi.org/10.3390/app10144869.
2. Chang, F.R.; Chen, J.L.; Chiu, H.F.; Wu, M.J.; Wu, Y.C. Acetogenins from seeds of *Annona reticulata*. *Phytochemistry* **1998**, *47*, 1057–1061. https://doi.org/10.1016/S0031-9422(98)80072-1.
3. Cárdenas, C.; Torres-Vargas, J.A.; Cárdenas-Valdivia, A.; Jurado, N.; Quesada, A.R.; García-Caballero, M.; Martínez-Poveda, B.; Medina, M.Á. Non-targeted metabolomics characterization of *Annona muricata* leaf extracts with anti-angiogenic activity. *Biomed. Pharmacother.* **2021**, *144*, 112263. https://doi.org/10.1016/j.biopha.2021.112263.

Figure 9:

1. Philip, J.Y.N.; Da Cruz Francisco, J.; Dey, E.S.; Buchweishaija, J.; Mkayula, L.L.; Ye, L. Isolation of anacardic acid from natural cashew nut shell liquid (CNSL) using supercritical carbon dioxide. *J. Agric. Food Chem.* **2008**, *56*, 9350–9354. https://doi.org/10.1021/jf801532a.
2. Kumar, P.P.; Paramashivappa, R.; Vithayathil, P.J.; Subba Rao, P.V.; Srinivasa Rao, A. Process for isolation of cardanol from technical cashew (*Anacardium occidentale* L.) nut shell liquid. *J. Agric. Food Chem.* **2002**, *50*, 4705–4708. https://doi.org/10.1021/jf020224w.
3. De Brito, E.S.; De Araújo, M.C.P.; Lin, L.Z.; Harnly, J. Determination of the flavonoid components of cashew apple (*Anacardium occidentale*) by LC-DAD-ESI/MS. *Food Chem.* **2007**, *105*, 1112–1118. https://doi.org/10.1016/j.foodchem.2007.02.009.

Figure 10:

1. Kakkar, A.; Verma, D.R.; Suryavanshi, S.; Dubey, P. Characterization of chemical constituents of *Calotropis procera*. *Chem. Nat. Compd.* **2012**, *48*, 155–157. https://doi.org/10.1007/s10600-012-0189-1.
2. Geiger, W.; Hesse, G.; Lettenbauer, G.; Schildknecht, H. Calotoxin aus Calotropin; zur Konstitution des Calotropagenins. *Naturwissenschaften* **1957**, *44*, 328. https://doi.org/10.1007/BF00630937.
3. Crout, D.H.G.; Hassall, C.H.; Jones, T.L. Cardenolides. Part VI. Uscharidin, calotropin, and calotoxin. *J. Chem. Soc.* **1964**, 2187–2194. https://doi.org/10.1039/JR9640002187.
